# Synthesis, Bioevaluation, Structure-Activity Relationship and Docking Studies of Natural Product Inspired (*Z*)-3-benzylideneisobenzofuran-1(*3H*)-ones as Highly Potent antioxidants and Antiplatelet agents

**DOI:** 10.1038/s41598-020-59218-6

**Published:** 2020-02-11

**Authors:** Bharti Rajesh Kumar Shyamlal, Lalit Yadav, Mohit K. Tiwari, Manas Mathur, Jaroslav I. Prikhodko, Irina V. Mashevskaya, Dharmendra K. Yadav, Sandeep Chaudhary

**Affiliations:** 1 Laboratory of Organic & Medicinal Chemistry, Department of Chemistry, Malaviya National Institute of Technology Jaipur, 302017 India; 2 School of Agriculture, Suresh Gyan Vihar University, Mahal Road, Jagatpura, Jaipur, 302017 India; 3 Department of Organic Chemistry, Perm State University, Bukireva Street, Perm, 614990 Russian Federation; 4 Gachon Institute of Pharmaceutical Sciences and Department of Pharmacy, College of Pharmacy, Gachon University of Medicine and Science, Incheon, 21936 South Korea

**Keywords:** Structure-based drug design, Structure-based drug design

## Abstract

For the first time, a series of highly potent natural product inspired substituted (*Z*)-3-benzylideneisobenzofuran-1(*3H*)-ones **28a-t**, embraced with electron-withdrawing groups (EWG) and electron-donating groups (EDG) at site I and site II, were prepared and assessed for their *in vitro* antioxidant activities (DPPH free radical scavenging assay) and arachidonic acid (AA)-induced antiplatelet activities using ascorbic acid (IC_50_ = 4.57 µg/mL) and aspirin (IC_50_ = 21.34 µg/mL), as standard references, respectively. In this study, compounds **28f-g**, **28k-l** and **28q** have shown high order of *in vitro* antioxidant activity. Infact, **28f** and **28k** were found to show ***10-folds*** and ***8-folds*** more antioxidant activity than ascorbic acid, respectively and was found to be the most active analogues of the series. Similarly, Compounds **28c-g**, **28k-l**, **28o** and **28q-t** were recognized as highly potent antiplatelet agents (upto ***6-folds***) than aspirin. Furthermore, *in silico* studies of the most active antioxidants **28f**, **28k** and **28l** and very active antiplatelet molecules **28f**, **28k**, **28l** and **28s** were carrying out for the validation of the biological results. This is the first detailed study of the discovery of several (*Z*)-3-benzylideneisobenzofuran-1(*3H*)-ones as highly potent antioxidants and antiplatelet agents.

## Introduction

**Figure 1:**
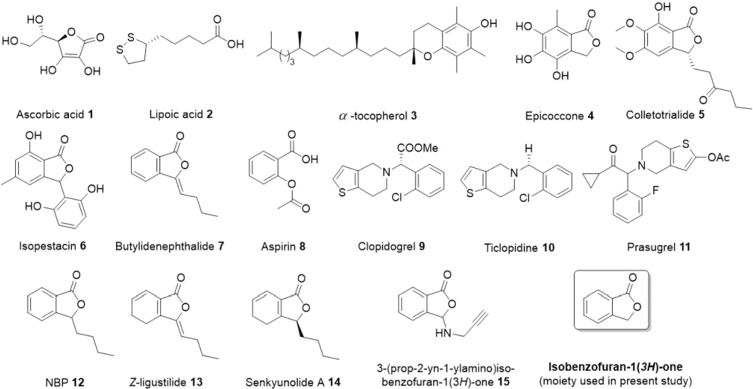
Structure of some naturally occurring/synthetic antioxidants **1–7** and antiplatelet agents **7–15.**

Isobenzofuran-1(*3 H*)-one, commonly known as “phthalide” is found in many naturally-occurring and pharmaceuticals important molecules^1^. This benzo-fused heterocyclic class of compounds are blended with several pharmacological properties such as anti-tumor^2^, anti-HIV^3^, anti-allergic^4^, antifungal^5^, antidiabetic^6^, antispasmodic^7^, anti-inflammatory^8^, pesticidal^9^, COX-2 inhibitor^10^, insecticidal^11^, anti-microbial^12^, herbicidal^13^, and anti-cancer^14^ etc. Several naturally occurring as well as synthetic molecules having isobenzofuran-1(*3H*)-ones have also been classified as promising antioxidants **1**–**7** (Fig. 1)^15–18^. Similarly, several natural and synthetic molecules **7–15** show promising platelet aggregation inhibitors^15^ (Fig. 1). Relevant to the present study, natural product inspired isobenzofuran-1(*3 H*)-ones are known to be potential antioxidant (**7**) and antiplatelet agents (**7**, **12–15**)^15,19–21^.

Earlier, Teng and his co-workers carried out extensive platelet aggregation inhibitory studies on butylidenephthalide **7** triggered by various inducers such as Arachidonic acid (AA), Adenosine diphosphate (ADP), platelet activation factor (PAF), etc. as well as the inhibition of thromboxane B2 (TXB2) formation caused by AA, collagen, ionophore A23187 and thrombin^20^. In this study, **7** showed significantly higher inhibitory efficacy in AA-induced platelet aggregation (IC_50_ = 70 µM) in comparison to collagen-induced aggregation (IC_50_ = 120 µM) in washed rabbit platelets. Teng *et al*. also suggested that butylidenephthalide **7** inhibits platelet aggregation mainly by inhibiting cyclooxygenase-1 (COX-1) enzyme leading to the reduction of thromboxane A2 (TBXA2) formation. Similar studies further confirms the above facts^15,21^. Overall, these studies revealed that (*Z*)-3-substituted-isobenzofuran-1(*3H*)-ones class of compounds exhibits AA-induced antiplatelet activities *via* inhibition of COX-1^20^. Nevertheless, there has been no detailed study on the AA-induced platelet aggregation inhibitory activities of (*Z*)-3-benzylidineisobenzofuran-1(*3H*)-ones. Therefore, there is a scope to develop a more potent isobenzofuran-1(*3H*)-ones having potential antioxidant as well as antiplatelet agents.

Synthetic/naturally isolated isobenzofuran-1(*3H*)-ones and its derivatives have attracted medicinal chemists and pharmacologists due to their pronounced biological activities and their potential applications as antioxidant as well as antiplatelet agents^15–21^. For example, ascorbic acid **1**^15^, Pestacin **17**^17^, Micromeriol **18**^22^, Pulvinate analogue **19**^23^, 5-(bis(3,4-dimethoxyphenyl)-methylene)furan-2(*5H*)-one **20**^24^, ailanthoidol **21**^25^, etc. were reported as antioxidants. It has been well documented that isobenzofuran-1(*3H*)-ones displayed good AA-induced platelet aggregation inhibitory activity as compared to ADP and collagen-induced factors. However, the AA-induced platelet aggregation inhibiting activities of (*Z*)-3-benzlidine-isobenzofuran-1(*3H*)-ones have never been explored. Nevertheless, so far, there is no detailed study done proving its inhibition by cyclooxygenase-1 (COX-1) enzyme.

**Figure 2:**
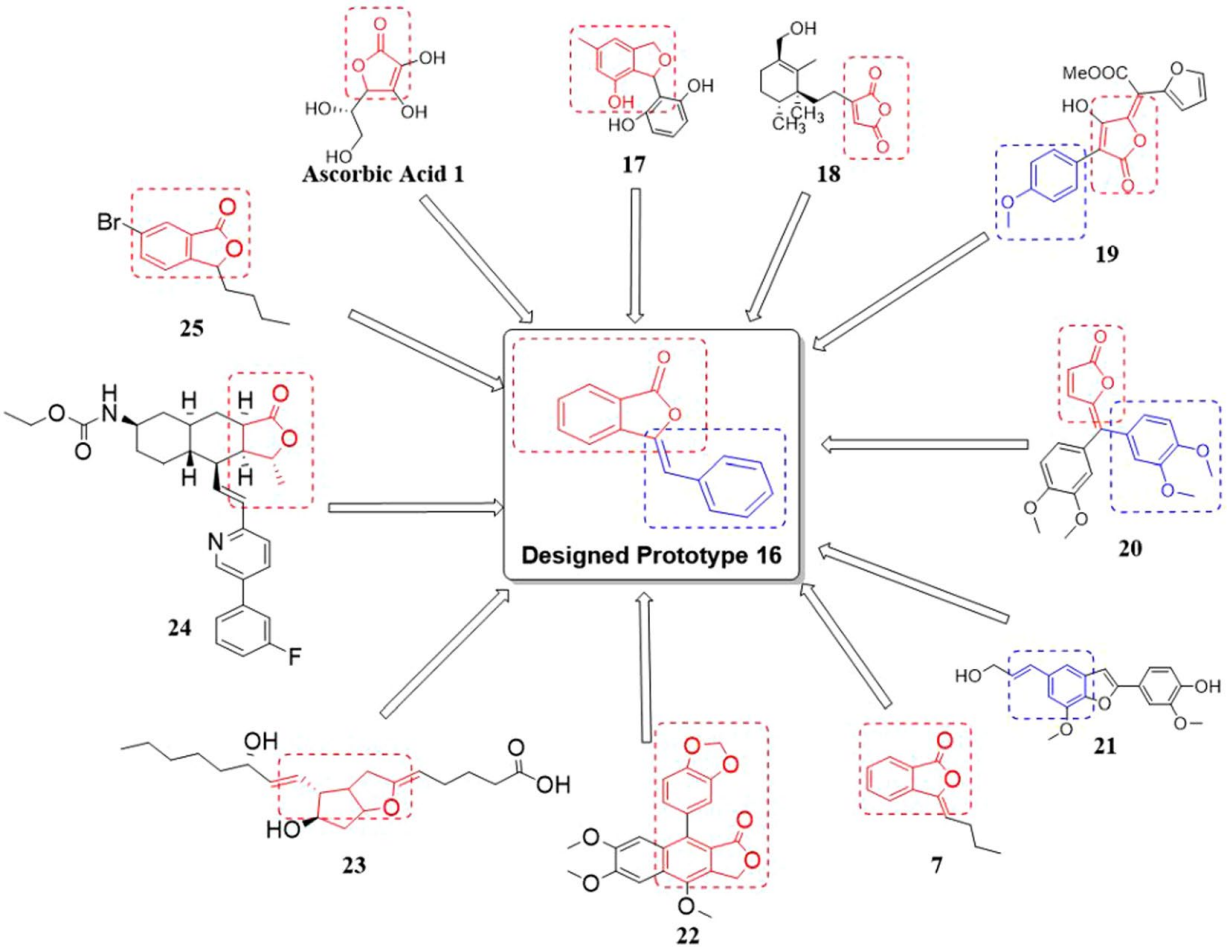
Design strategy for the target compound (*Z*)-3-benzylideneisobenzofuran-1(*3 H*)-one **16** as an antioxidant and as an antiplatelet agent.

Therefore, in our endeavor in search for novel bioactive heterocycles and also based on the above facts, we have designed **prototype 16** i.e. C-3 (*Z*)-benylidine-isobenzofuran-1(*3H*)-ones, incorporating similar sub-structural units of **1**, **7** and **17**–**25** (Fig. 2) and assessed their antioxidant and AA-induced platelet aggregation inhibiting activities with the anticipation that the (*Z*)-3-benylidine-isobenzofuran-1(*3H*)-ones would also show promising antioxidant as well as antiplatelet activity. Hence, the designed prototype **16** is derived from sub-structure in which the molecule, as a whole or in part of, is responsible for antioxidant activity. Similarly, butylidenephthalide **7** (dual potential as antioxidant and antiplatelet agent)^15^, Justicidin A **22**^26^, prostacyclin (Trade name: Epoprostenol, prostaglandin I_2_) **23**^27^, Zontivity (Trade name: Vorapaxar) **24**^28^, 6-bromo-3-butylisobenzofuran-1(*3H*)-one (bromo derivative of NBP) **25**^29^, etc. were reported to show promising platelet aggregation inhibitory activity (Fig. 2).

In the present study, we report the synthesis a series of functionalized (Z)-3-benzylidineisobenzofuran-1(*3H*)-ones **28a-t**, their antioxidant and AA-induced antiplatelet activities, and structure-activity relationship (SAR) studies. Although compounds **28a-j**, **28m-r** and **28t** have been prepared earlier by other routes;^30^ including our route;^31^ however, for the first time, substrate-controlled silver oxide nanoparticle (Ag_2_ONPs)-catalyzed synthesis of compound **28a-t** have been prepared. Various substituted (Z)-3-benzylidineisobenzofuran-1(*3H*)-ones **28a-t** have shown high order of antioxidant and AA-induced antiplatelet activities using ascorbic acid and aspirin taken as standard reference, respectively. We also perform the *in silico* studies of most active compounds **28f**, **28k, 28l** and **28s** for the validation of biological results.

## Results and Discussion

**Figure 3:**
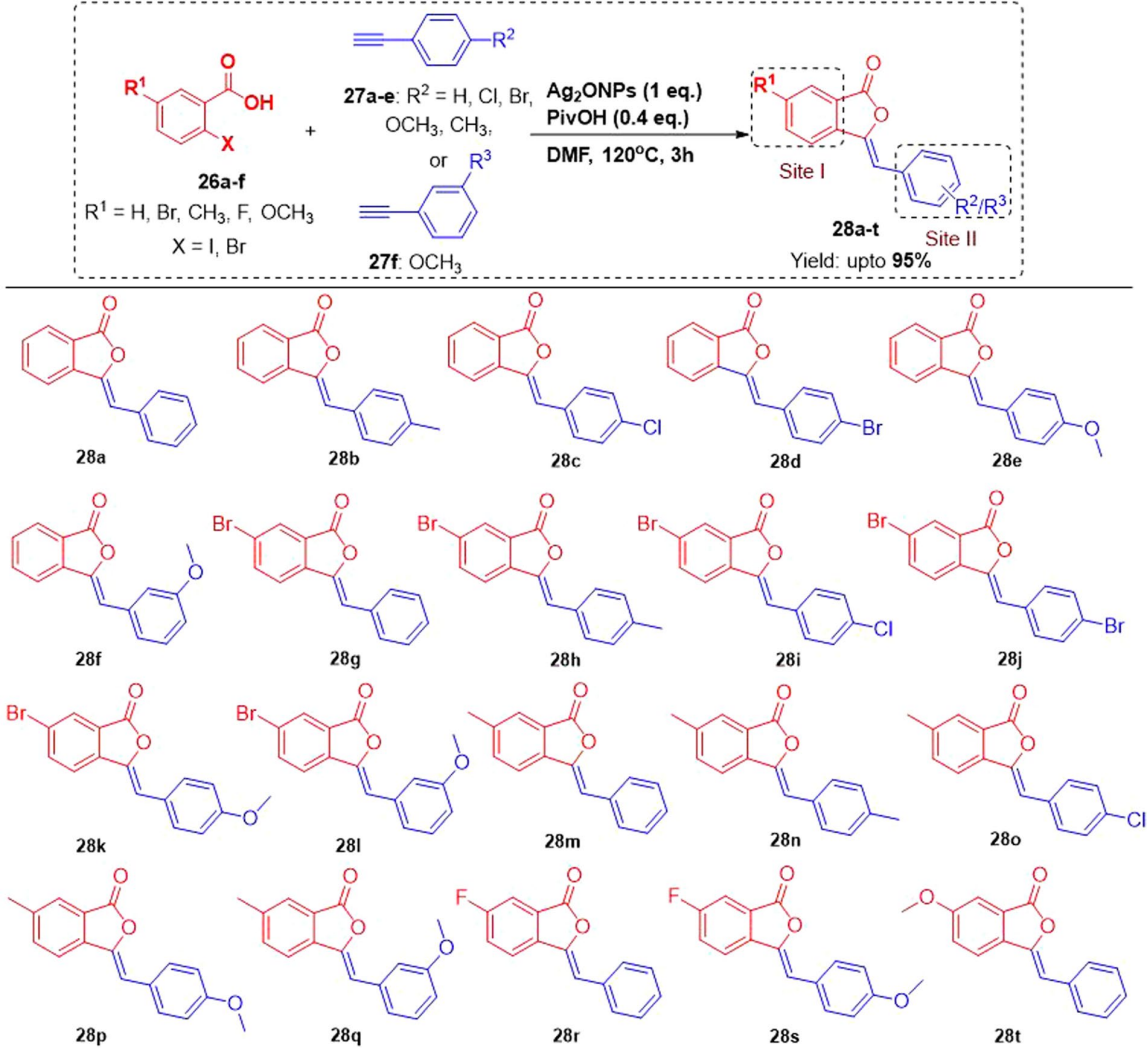
Synthesis of functionalized (*Z*)-3-benzylideneisobenzofuran-1(*3H*)-ones **28a-t** using our procedure.

Recently, an efficient synthesis of (*Z*)-3-benzylideneisobenzofuran-1(*3H*)-ones in a highly regioselective manner in excellent yields has been reported by our group^31^. Ag_2_ONPs-mediated reaction of several substituted 2-iodobenzoic acids **26a-f** (R^1^ = H, Br, CH_3_, F, OCH_3_; X = Br, I) with various *p*-/*m-*substituted terminal alkynes **27a-f** in the presence of pivalic acid as additive in DMF as solvent at 120 °C for 3 h furnished (*Z*)-3-benzylideneisobenzofuran-1(*3H*)-ones **28a-t** upto 95% yields via 5*-exo-dig* cyclization strategy (Fig. 3). The protocol is operationally simple and show functional group tolerance. The structures of compounds **28a-t** were confirmed by their spectroscopic analytical data (^1^H and ^13^C NMR, FT-IR and HRMS) (Fig. 3).

Then, the functionalized (*Z*)-3-benzylideneisobenzofuran-1(*3H*)-ones **28a-t** were assessed for their *in vitro* antioxidant activity using literature procedure (Fig. 3)^31,32^. The results are shown in Table 1. Primarily, (*Z*)-3-benzylideneisobenzofuran-1(*3H*)-one **28a**, unsubstituted at site I and II, was assessed for its *in vitro* antioxidant activity. It was observed that **28a (**IC_50_ = 14.38 ± 0.09 μg/mL), demonstrated reduced potency than the standard reference, ascorbic acid (IC_50_ = 4.57 μg/mL) (Table 1, Entry 1). On the other hand, **28b** (IC_50_ = 8.88 ± 0.12 μg/mL) and **28c** (IC_50_ = 6.33 ± 0.08 μg/mL) having *p*-methyl (*p*-CH_3_) and *p*-chloro (*p*-Cl) substitutents considerably augments the antioxidant activity in comparison to **28a** (Table 1, entry 2–3). Introduction of the substitutent having larger size i.e., *p*-Br group in the case of compound **28d** (IC_50_ = 52.34 ± 0.29 μg/mL), drastically diminishes the antioxidant activity (Table 1, entry 4). Furthermore, incorporation of methoxy (OMe) substitutent at site II exhibited incremental effect in the antioxidant activity. Likewise, Compound **28e** (IC_50_ = 34.41 ± 0.94 μg/mL), having *p*-OMe substitution at site II exhibited good activity as compared to **28d** (Table 1, entry 5); surprisingly, compound **28f** (IC_50_ = 0.41 ± 0.12 μg/mL), having *m*-methoxy (*m*-OMe) group at site II demonstrated high order of antioxidant activity in comparison with reference standard (Table 1, entry 6). Infact, **28f** displayed 10-folds more activity than ascorbic acid.

**Table 1:** *In vitro* antioxidant activity (DPPH free radical scavenging assay) and *In vitro* AA-induced antiplatelet activity of functionalized (*Z*)-3-benzylideneisobenzofuran-1(*3H*)-ones **28a-t**.

Entry. No.	Comp. No.	R^1^	R^2^	R^3^	Antioxidant Activity^[a,b]^ (IC_50_ in µg/mL)	AA-induced antiplatelet activity^[c,d]^ (IC_50_ in µg/mL)
1	**28a**	H	H	H	14.38 ± 0.09	64.57 ± 0.58
2	**28b**	H	CH_3_	H	8.88 ± 0.12	44.66 ± 0.41
3	**28c**	H	Cl	H	6.33 ± 0.08	**19.57 ± 0.28**
4	**28d**	H	Br	H	52.34 ± 0.29	**12.86 ± 0.11**
5	**28e**	H	OCH_3_	H	34.41 ± 0.94	**14.00 ± 0.17**
6^[e]^	**28f**	H	H	*m*-OCH_3_	**0.41 ± 0.12**	**4.20 ± 0.28**
7	**28g**	Br	H	H	**1.59 ± 0.55**	**16.28 ± 0.25**
8	**28h**	Br	CH_3_	H	65.91 ± 0.75	29.32 ± 0.38
9	**28i**	Br	Cl	H	28.61 ± 3.11	31.34 ± 0.36
10	**28j**	Br	Br	H	20.36 ± 0.27	79.57 ± 0.64
11	**28k**	Br	OCH_3_	H	**0.55 ± 0.15**	**7.28 ± 0.48**
12^[e]^	**28l**	Br	H	*m*-OCH_3_	**0.73 ± 0.44**	**4.20 ± 0.28**
13	**28m**	CH_3_	H	H	7.23 ± 0.04	47.93 ± 0.44
14	**28n**	CH_3_	CH_3_	H	45.60 ± 0.91	25.60 ± 0.31
15	**28o**	CH_3_	Cl	H	7.60 ± 0.38	**19.64 ± 0.28**
16	**28p**	CH_3_	OCH_3_	H	16.34 ± 0.27	24.64 ± 0.42
17^[e]^	**28q**	CH_3_	H	*m*-OCH_3_	**3.83 ± 0.88**	**9.60 ± 0.62**
18	**28r**	F	H	H	13.20 ± 0.21	**11.37 ± 0.67**
19	**28s**	F	OCH_3_	H	25.52 ± 0.30	**3.25 ± 0.18**
20	**28t**	OCH_3_	H	H	21.21 ± 0.12	**5.16 ± 0.32**
21	Ascorbic acid **1**	—	—	—	**4.57**	—
22	Acetyl salicylic acid **8**	—	—	—	—	**21.34 ± 1.09**

^[a]^The results are articulated as a mean ± standard deviation (n = 3). ^[b]^For details, see ref. ^37^. ^[c]^For details, see refs. ^20,34^. ^[d]^A mean of 03 experimental data are determined. ^[e]^The OMe group at R^3^-position of the molecule is present at the *meta*-position; however, all the group at R^2^ are at para-position.

Also, parallel trends were observed when **28g-l** having 6-bromo substitutent at site I along with H/CH_3_/Cl/Br/*p*-OMe/*m*-OMe groups at *p*-/*m*-position of site II were analyzed (Table 1, entry 7–12). Sequentially, **28g** (IC_50_ = 1.59 ± 0.55 μg/mL), 6-Br substitution at site I and unsubstituted at site II, displayed approximately 3-folds greater potency (Table 1, Entry 7). Nonetheless, **28h-j** having bromo substitution at C-6 position of site I and *p*-CH_3_, *p*-Cl and *p*-Br substitution at site II do not have valuable effect on the antioxidant activity (Table 1, entry 8–10). But, compound **28k** (IC_50_ = 0.55 ± 0.15 μg/mL) and **28l** (IC_50_ = 0.732 ± 0.44 μg/mL), having bromo substitution at C-6 position of site I and *p*-OMe/*m*-OMe group at site II showed highly potent activity in comparison to ascorbic acid (Table 1, Entry 11–12). Infact, **28k** and **28l**, showed greater than ***8-folds*** and ***7-folds*** more activity than the standard reference (Table 1, Entry 11–12).

In parallel, further developments were noticed when compounds **28m-q** having electron-donating group (EDG) i.e., methyl groups at C-6 position of site I along with H/CH_3_/Cl/OMe groups at *p*-/*m*-position of site II were analyzed (Table 1, entry 13–17). Compound **28m** (IC_50_ = 7.23 ± 0.04 μg/mL), electron-donating group (CH_3_ group) at C-6 position of site I and no substitution at site II, displayed potential activity than ascorbic acid and showed greater activity than that of **28a** (Table 1, Entry13). However, **28n-p** having CH_3_ group at C-6 position of site I and *p*-CH_3_, *p*-Cl and *p*-OMe substitutions at site II also showed potency except **28n** (Table 1, entry 14–16). Nonetheless, compound **28q** (IC_50_ = 3.83 ± 0.88 μg/mL), having CH_3_ group at C-6 position of site I and *m*-OMe group at site II showed more antioxidant potency than ascorbic acid (Table 1, Entry17). Chronologically, EWG (F) group at C-6 position of site I were also analyzed (**28r-s**) and it was observed that both the compounds exhibited decreased potency than the standard reference, thereby, decreasing the significance of EWG group (entry 18–19, Table 1).

The SAR analysis revelaed that the five compounds i.e. **28f-g**, **28k-l** and **28q**, exhibited promising antioxidant activity with the IC_50_ values in the range 0.41–3.83 µg/mL. Further, no substitution at C-6 position of site I and *m*-OMe group at site II (i.e. compound **28f**), showed best antioxidant potency than ascorbic acid. However, EDG substitution at site I (Br, CH_3_) and H/*p*-OMe/*m*-OMe substitution on site II also accounts for promising activity (i.e. compound **28g**, **28k-l** and **28q**). EWG group (fluoro group) at site I do not show favourable effects on the antioxidant activity [strong EWG-containing phenyl acetylenes (F, NO_2_, CN etc.) do not undergoes reaction under our optimized reaction conditions]. In addition, *m*-OMe group at site II augment antioxidant activity. Therefore, to examine the effect of OMe group at site I, we prepared **28t**. It was found that compound **28t** (IC_50_ = 21.21 ± 0.12 μg/mL), having OMe group at C-6 position of site I and no substitution at site II showed diminished activity in comparison with the standard reference (Table 1, entry 20). Thus, OMe group showed advantageous effect on site II rather than site I. Finally, the structure-activity relationship studies illustrates that the two compounds, **28f** and **28k**, showed ***10-folds*** and ***8-folds*** higher antioxidant potency than commercially used antioxidant refered in the present study.

Since this scaffold have shown promising inhibition of platelet aggregation; compounds **28a-t** were also tested for their AA-induced inhibition of platelet aggregation using aspirin as the standard reference (Table 1)^33,34^. As it has been observed from Table 1, compounds **28a-f** were prepared having no substitutions at C-6 position of site I and H/CH_3_/Cl/Br/*p*-OMe/*m*-OMe substitutions at site II, respectively; **28a** (IC_50_ = 64.57 ± 0.58 μg/mL) demonstrated significant AA-induced antiplatelet activity in comparison with the standard reference aspirin (IC_50_ = 21.34 ± 1.09 μg/mL; Table 1, entry 1). Shifting to CH_3_ group at *p*-position of site II (**28b**) improves IC_50_ value to 44.66 ± 0.41 μg/mL (Table 1, entry 2). However, the antiplatelet activity was improved tremendously when Cl/Br/OMe groups at site II were introduced. Compounds **28c-e** showed IC_50_ value of 19.57 ± 0.28 μg/mL, 12.86 ± 0.11 μg/mL and 14.00 ± 0.17 μg/mL, respectively which were found to be more active than aspirin (Table 1, entries 3–5). As also been noticed in the case of antioxidant activities of these compounds, **28f** having *m*-OMe group at site II exhibited five-folds more efficacious (IC_50_ = 4.20 ± 0.28 μg/mL) than the standard reference (Table 1, entry 6). Furthermore, **28g-l** were analyzed having bromo substitution at C-6 position of site I along with H/CH_3_/Cl/Br/*p*-OMe/*m*-OMe groups at *p*-/*m*-position of site II (Table 1, entry 7–12). It has been interpreted that introduction of bromine group at site I improves antiplatelet activity (IC_50_ = 16.28 ± 0.25 μg/mL) upto four-folds than **28a** and more active than aspirin (Table 1, entry 7). In contrast, compounds **28h-j** having bromo substitution at C-6 position of site I along with CH_3_/Cl/Br groups at *p*-position of site II showed decreased activity i.e., IC_50_ values of 29.32 ± 0.38 μg/mL, 31.34 ± 0.36 μg/mL and 79.57 ± 0.64 μg/mL, respectively, than the standard reference (Table 1, entry 8–10). The activity has been increased effectively if OMe groups at *p*-/*m*-position of site II is introduced. Compounds **28k (**IC_50_ = 7.28 ± 0.48 μg/mL) and **28l** (IC_50_ = 4.20 ± 0.28 μg/mL) having bromo substitution at C-6 position of site I along with *p*-/*m*-OMe substitutions on site II showed 3–5 folds greater antiplatelet potency than the standard reference, respectively (Table 1, entry 11–12).

Similarly, we analyzed the effect of EDG group at site I and prepared **28m-q** having H/CH_3_/Cl/OMe groups at *p*-/*m*-position of site II (Table 1, entry 13–17). Introduction of CH_3_ group at site I (**28m**) showed more aggregation inhibitory activity (IC_50_ = 47.93 ± 0.44 μg/mL) than **28a** and less activity than **28g** (Table 1, entry 13). The activity has been found comparable to aspirin if CH_3_/Cl substitutions on site II were also introduced (Table 1, entry 14–15). While **28p (**IC_50_ = 24.64 ± 0.42 μg/mL) with methyl (CH_3_) group at C-6 position of site I and *p*-OMe substitutions on site II exhibited comparable activity; Compound **28p (**IC_50_ = 24.64 ± 0.42 μg/mL) with methyl (CH_3_) group at C-6 position of site I and *m*-OMe substitutions on site II showed two-folds more potency than the standard reference (Table 1, entry 16–17). Sequentially, (EWG) group i.e., fluoro groups at C-6 position of site I were also analyzed (**28r-s**) and it was observed that both the compounds, **28r** (IC_50_ = 11.37 ± 0.67 μg/mL) and **28s** (IC_50_ = 3.25 ± 0.18 μg/mL), exhibited ~two- to seven-folds more potency than aspirin, respectively (Table 1, entry 18–19). Infact, Compound **28s** showed highest potency among the series. Compound **28t** (IC_50_ = 5.16 ± 0.32 μg/mL), in contract to the antioxidant activity, showed four-folds more antiplatelet potency than the standard reference (Table 1, entry 20). Overall, it interprets that introduction of EWG or EDG at site I improves antiplatelet activity than aspirin. Similarly, introduction of *p*-/*m*-OMe on site II leads to either more activity or comparable to the standard reference.

###  Molecular docking studies

All the most active compounds (**28f**, **28k** and **28l**) along with one inactive compound **28d** were further analyzed for their *in silico* studies using the reported protocol where the antioxidant target (PDB ID: 3MNG) were taken to explore the orientations and binding affinities of the target compounds in order the observe the difference in the docking score of active and inactive compounds^35,36^. Wild type human antioxidant enzyme Peroxiredoxins (Prdxs) was chosen, containing essential cysteine residues as catalyst and thioredoxin as an electron donor, which help in scavenging peroxide and are involved in the metabolic cellular response to reactive oxygen species^36^. It has been confirmed that the ascorbate-mediated reduction of protein sulfenic acids represents a modification of the peroxiredoxin-thiol-specific antioxidant paradigm, which directly confirms the interlinking of peroxiredoxins with standard drug ascorbic acid (vitamin C)^36^. Therefore, the interlinking of standard reference ascorbic acid with peroxiredoxins direct us to perform molecular docking studies on this enzyme.

Similarly, to study the binding modes of the five active molecules (**28k, 28s, 28f, 28l** and **28j**) in the cyclooxygenase-1 (COX-1) enzyme against platelet aggregation inhibitory activity, we performed molecular docking study with aspirin (reference compound) on COX-1 domain antiplatelet target (PDB ID: 2OYE) using Surflex–Dock using the reported procedure (see details in supporting information)^37^.

#### Antioxidant molecular docking studies

**Figure 4:**
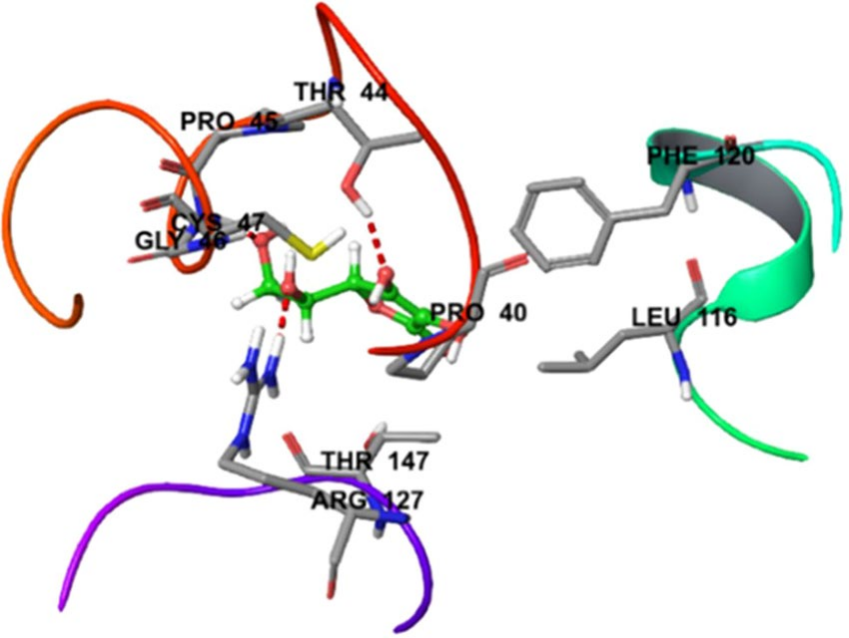
The binding interaction of standard drug **ascorbic acid** (docking score = 3.1764).

**Figure 5:**
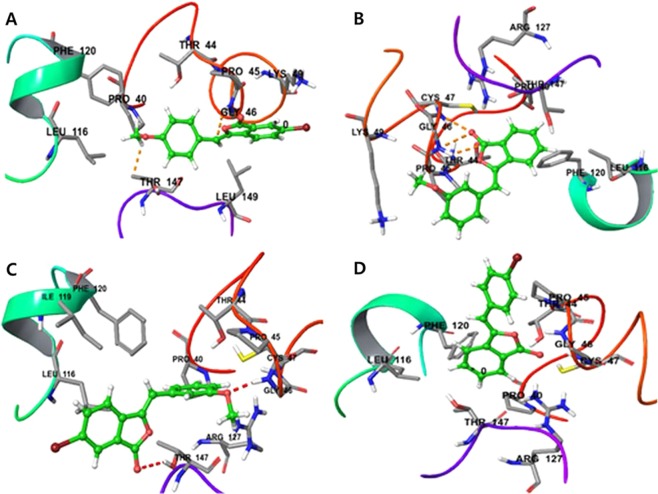
The docking outcomes for compound **28k** (**A**), **28f** (**B**) **28l** (**C**) and **28d** (**D**) having docking score of 3.9321, 4.6899, 3.4080 and 2.9813 respectively.

The docking outcomes for the **ascorbic acid** against antioxidant target reflected a high binding affinity (docking score = **3.1764)** as shown in Fig. 4. The active compound **28k**, **28f** and **28l** displayed docking results against antioxidant target (PDB ID: 3MNG) exhibited a docking score of **3.9321**, **4.6899** and **3.4080**, respectively, therby reflecting their high binding affinity. These values were found to be more than that of ascorbic acid. Therefore, **28k**, **28f** and **28l** showed elevated binding affinity and hydrophobic interaction which are responsible for more stability and activity (Fig. 5A–C). Likewise, the inactive compound **28d** (Fig. 5D) was found to show less binding affinity than the ascorbic acid as indicated by its docking score of **2.9813**. This showed low binding affinity and weak hydrophobic interaction which may be responsible for less stability and activity (see details in supporting information).

#### Antiplatelet molecular docking studies

**Figure 6:**
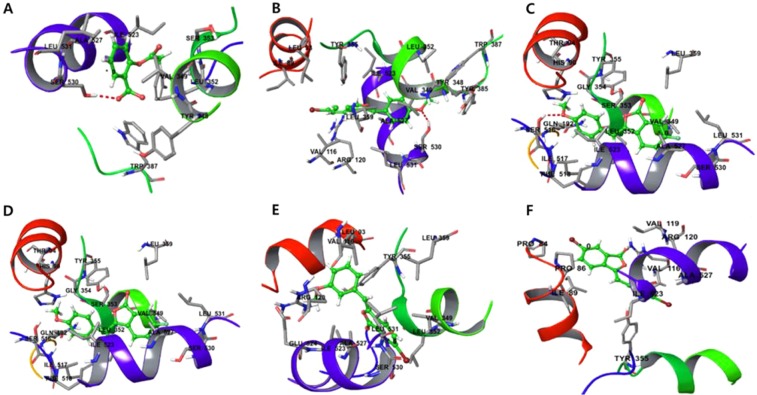
The docking score of **28k** (**A**), **28s** (**B**), **28f** (**C**), **28l** (**D**), **28j** (**E**) and **aspirin** (**F**) showed elevated binding affinity in terms of docking score of 5.1953, 5.7131, 5.1010, 5.1184, 4.1714 and 4.4803 respectively.

Likewise, the antiplatelet docking outcomes for aspirin against target (PDB ID: 2OYE) revealed low docking score (total score of **4.4803)** thereby reflecting its lower binding affinity (Fig. 6A). The docking outcomes for **28k, 28s 28f**, and **28l** against PDB ID: 2OYE reflected docking score designated by a total score of **5.1953, 5.7131**, **5.1010** and **5.1184**, respectively thereby indicating a high binding affinities which were found to be more than standard reference aspirin. Therefore, **28k**, **28s**, **28f** and **28l** elevated binding affinity and hydrophobic interaction which are responsible for more stability and activity (Fig. 6B–E). In contrast, the antiplatelet docking outcomes for the inactive compound **28j** against target (PDB ID: 2OYE) showed lower docking score (total score = **4.1714**) thereby reflecting lower binding affinity which, in turn, is found to be lesser than the standard reference. Thus, the bound compound **28j** showed weak hydrophobic interaction which leads to low binding affinity thereby responsible for less stability and activity of the molecule (Fig. 6F).

## Conclusions

We disclose the first detailed study for the identification of (*Z*)-3-benzylideneisobenzofuran-1(*3H*)-one analogues **28a-t** as highly potent antioxidant and AA-induced antiplatelet agents. Five isobenzofuran-1(*3H*)-one analogues **28f-g**, **28k-l** and **28q** were found to be the active compounds of the series in DPPH assay. Infact, two compounds, **28f** and **28k**, showed ***10-folds*** and ***8-folds*** more antioxidant activity than ascorbic acid, respectively. Similarly, twelve isobenzofuran-1(*3H*)-one analogues **28c-g, 28k-l, 28o**, and **28q-t** exhibited highly potent (upto ***6-folds***) platelet aggregation inhibitors as compared to aspirin in AA-induced antiplatelet biological assay. Furthermore, compounds **28f**, **28k**, **28l** and **28s** were analyzed through docking studies for the justification of the obtained results. These highly active potent molecules were found commendable of additional structural optimization and advancement as potential antioxidant and/or antiplatelet representatives.

## Materials and Methods

### Chemistry

#### General

All the glass apparatus were oven dried prior to use. Melting points were taken in open capillaries on Sisco melting point apparatus and are presented uncorrected. All the AR grade chemicals were used as supplied from commercial source (Sigma Aldrich, TCI, Alpha Aesar, Spectrochem etc.) and used without further purification. Laboratory grade commercial reagents and solvents were purified by standard procedures prior to use. The silica gel (100–200 Mesh) used for column chromatography were supplied either from QualigensTM (India) or Rankem (India), unless otherwise noted. UV fluorescence and Iodine vapor served as the visualizing agent for thin layer chromatography (Merck silica gel 60 F_254_ precoated plates (0.25 mm). ^1^H NMR and ^13^C NMR spectral data were recorded on a JEOL ECS-400 (2-channel support with an adaptable broadband RF execution) spectrometer working at 400 MHz for ^1^H and 100 MHz for ^13^C) utilizing CDCl_3_ as a solvent. The ^1^H-NMR (400 MHz) chemical shifts were measured relative to CDCl_3_ as the internal reference (CDCl_3_: δ = 7.249 ppm). Tetramethylsilane (δ 0.00 ppm) served as an internal standard in ^1^H NMR and CDCl_3_ (δ 77.0 ppm) in ^13^C NMR. Chemical shifts are reported in parts per million. Splitting patterns are described as singlet (s), doublet (d), double doublet (dd), triplet (t), multiplet (m), and broad (br). Infrared spectra were recorded on a FT-IR Spectrum 2 (Perkin-Elmer) spectrophotometer. Electron Impact Mass Spectroscopy (HR-EIMS) data were obtained from Xevo G2-S Q-Tof (Waters, USA) compatible with ACQUITY UPLC® and nano ACQUITY UPLC® systems. The BUCHI Rotavapor R-210 was used for drying and concentration of the solvents. All animal experiments were performed in compliance with the relevant laws and guidelines of Suresh Gyan Vihar University, and approved by the institutional animal ethical committee(s).

#### General procedure (GP) for the synthesis of (*Z*)-3-benzylideneisobenzofuran-1(*3H*)-ones 28a-t

Substituted halo-aromatic carboxylic acid **26a**-**f** (1.21 mmol, 1.0 eq.), substituted terminal alkynes **27a-e/27 f** (1.21 mmol, 1.0 eq.) were dissolved in dry DMF (2 mL) taken in a round-bottom flask; added Ag_2_ONPs (1.21 mmol, 1.0 eq.) as well as the PivOH (0.484 mmol, 0.4 eq.) as additive. The reaction mixture was stirred at 120 °C for 3 h. After completion of the reaction, the reaction mixture was cooled to room temperature and dilute it with EtOAc (10 mL) and filtered through a celite bed and then, washed further with EtOAc (15 mL). The combined organic solvents were extracted with EtOAc (3 × 10 mL), washed with water (2 × 20 mL) and then with saturated NaCl solution (20 mL). The organic layer was dried over anhyd. Na_2_SO_4_ and evaporated under decreased pressure. The crude product was purified by column chromatography over silica gel (100–200 mesh size) using 2% EtOAc: Hexane as an eluant to furnish **28a-t**.

The detailed spectral data of compounds **28a-j**, **28m-r** and **28t** are given in the supporting information.

#### (*Z*)-6-bromo-3-(4-methoxybenzylidene) isobenzofuran-1(3*H*)-one (28k)

Light bluish solid, m.p. 180–184 °C, 61% yield. ^1^H NMR (400 MHz, CDCl_3_) δ 8.04–8.03 (m, 1 H), 7.80–7.76 (m, 3 H), 7.59 (d, *J* = 8.3 Hz, 1 H), 6.94 (d, *J* = 8.8 Hz, 2 H), 6.37 (s, 1 H), 3.84 (s, 3 H); ^13^C NMR (100 MHz, CDCl_3_) δ 165.81, 160.13, 142.38, 139.50, 137.56, 131.93, 128.52, 125.63, 124.86, 123.15, 121.01, 114.45, 108.00, 55.45; HRMS (ESI) Calculated for C_16_H_11_BrO_3_ [M + H]^+^: 330.9965, found 330.9967; FT-IR (Neat, cm^-1^): 1752, 1596, 1505, 1450, 1246, 1022, 970, 822, 525.

#### (*Z*)-6-bromo-3-(3-methoxybenzylidene) isobenzofuran-1(3*H*)-one (28 l)

Light yellow solid, m.p. 158–160 °C, 70% yield. ^1^H NMR (400 MHz, CDCl_3_) δ 8.05 (s, 1 H), 7.82–7.80 (m, 1 H), 7.63 (d, *J = *8.3 Hz, 1 H), 7.39–7.38 (m, 2 H), 7.31 (t, *J = *8.1, 1 H), 6.88 (d, *J = *9.3 Hz, 1 H), 6.38 (s, 1 H), 3.85 (s, 3 H); ^13^C NMR (100 MHz, CDCl_3_) δ 165.50, 159.90, 144.04, 139.28, 137.71, 134.08, 129.87, 128.62, 125.23, 123.83, 123.01, 121.33, 115.16, 114.90, 107.97, 55.44; HRMS (ESI) Calculated for C_16_H_11_BrO_3_ [M + H]^+^: 330.9965, found 330.9964; FT-IR (Neat, cm^-1^): 2922, 2852, 1765, 1667, 1584, 1453, 1241, 1171, 1041, 978, 772, 687, 490.

#### (*Z*)-6-fluoro-3-(4-methoxybenzylidene) isobenzofuran-1(3*H*)-one (28 s)

greenish white solid, m.p. 164–168 °C, 71% yield. ^1^H NMR (400 MHz, CDCl_3_) δ 7.80–7.78 (d, *J = *12.8 Hz, 2 H), 7.77–7.71 (m, 1 H), 7.58–7.56 (dd, *J = *7.1 Hz, 2.1 Hz, 1 H), 7.45–7.40 (m, 1 H), 6.96–6.93 (d, *J = *8.8 Hz, 2 H), 6.34 (s, 1 H), 3.85 (s, 3 H); ^13^C NMR (100 MHz, CDCl_3_) δ = 166.19, 164.47, (*J*_C-F_ = 250 Hz), 159.98, 142.38, 136.92, 131.74, 125.72, 123.04 (*J*_C-F _= 25 Hz), 121.56, (*J*_C-F _= 9 Hz), 114.41, 111.84, (*J*_C-F _= 24 Hz), 107.18, 55.44; HRMS (ESI) Calculated for C_15_H_10_FO_3_ [M + H]^+^: 271.0765, found 271.0767; FT-IR (Neat, cm^-1^): 2921, 2851, 1761, 1602, 1493, 1254, 1164, 982, 807, 538.

### Biological methods

***In vitro*** **antioxidant DPPH radical scavenging activity:**^38^ See SI.

**Platelet aggregation inhibitory activity evaluation:**^20,34^ See SI.

**Molecular docking studies:**^39^ see SI.

## Supplementary information

Supporting information .

## Data Availability

All data generated or analyzed during this study are included in this published article.
