# Supplementary material for: Synthesis, Bioevaluation, Structure-Activity Relationship and Docking Studies of Natural Product Inspired (Z)-3-benzylideneisobenzofuran-1(3H)-ones as Highly Potent antioxidants and Antiplatelet agents
Source: Sci Rep. 2020 Feb 11;10:2307. doi: 10.1038/s41598-020-59218-6 (PMC7012914; doi:10.1038/s41598-020-59218-6)
Supplement: Supplementary file 1 — Supporting information . [file 41598_2020_59218_MOESM1_ESM.docx]

Supporting Information

Synthesis, Bioevaluation, Structure-Activity Relationship and Docking Studies of Natural Product Inspired (*Z*)-3-benzylideneisobenzofuran-1(*3H*)-ones as Highly Potent antioxidants and Antiplatelet agents

Bharti Rajesh Kumar Shyamlal,^1^ Lalit Yadav,^1^ Mohit K. Tiwari,^1^ Manas Mathur,^2^ Jaroslav I. Prikhodko,^3^ Irina V. Mashevskaya,^3^ Dharmendra K. Yadav,^4,*^ and Sandeep Chaudhary^1,*^

**Mr. Bharti Rajesh K. Shyamlal, Mr. Lalit Yadav, Mr. Mohit K. Tiwari**
^1^Laboratory of Organic & Medicinal Chemistry, Department of Chemistry, Malaviya National Institute of Technology, Jawaharlal Nehru Marg, Jaipur-302017, India.
**Dr. Manas Mathur**

^2^School of Agriculture, Suresh Gyan Vihar University, Mahal Road, Jagatpura, Jaipur-302017, India.

**Mr. Jaroslav I. Prikhodko, Prof. Irina V. Mashevskaya**

^3^Department of Organic Chemistry, Perm State University, Bukireva Street, Perm 614990, Russian Federation.

**Dr. Dharmendra K. Yadav** (*Corresponding author)
^4^Gachon Institute of Pharmaceutical Sciences and Department of Pharmacy, College of Pharmacy, Gachon University of Medicine and Science, Incheon, 21936, South Korea.

Tel: +82-32-820-4947

Email: <dharmendra30oct@gmail.com>

**Prof. Sandeep Chaudhary** (*Corresponding author)

^1^Laboratory of Organic & Medicinal Chemistry, Department of Chemistry, Malaviya National Institute of Technology, Jawaharlal Nehru Marg, Jaipur-302017, India.
Tel: 91-141-2713319

Fax: 91-141-2529029

E-mail: [schaudhary.chy@mnit.ac.in](file:///D:\wipro12\Downloads\schaudhary.chy@mnit.ac.in)

| **S. No.** | **Contents** | **Page No.** |
| --- | --- | --- |
| **1.** | Characterization data of **(28a-t)**. | 2-4 |
| **2.** | ^1^H & ^13^C NMR spectral data of all the synthesized compounds **28a**-**t**. | 5-24 |
| **3.** | Biological methods | 25-26 |
| **4.** | Detailed Description for Antioxidant molecular docking for binding studies | 27-28 |
| **5.** | Detailed Description for Antiplatelet molecular docking for binding studies | 28-30 |
| **6.** | References | 31 |

**1. Characterization data of (28a-t).**

**(Z)-3-benzylideneisobenzofuran-1(3H)-one (28a)**^1^: White solid, m.p. 92-94˚C, 95% yield. ^1^H NMR (400 MHz, CDCl_3_) δ 7.94 (d, *J* = 7.7 Hz, 1H), 7.85 (d, *J* = 7.2 Hz, 2H), 7.79–7.70 (m, 2H), 7.55 (t, *J* = 7.4 Hz, 1H), 7.41 (t, *J* = 7.3 Hz, 2H), 7.33–7.29 (m, 1H), 6.43 (s,1H**)**; ^13^C NMR (100 MHz, CDCl_3_) δ 167.16, 144.67, 140.71, 134.57, 133.17, 130.21, 129.87, 128.87, 128.52, 125.69, 123.53, 119.90, 107.15; HRMS (ESI) Calculated for C_15_H_10_O_2_ [M+H]^+^: 223.0754, found: 223.0755; FT-IR (Neat, cm^-1^):1760, 1660, 1607, 1469, 1447, 1270, 754, 682.

**(Z)-3-(4-methylbenzylidene) isobenzofuran-1(3H)-one** (**28b**)^1^: White solid, m.p. 140-144˚C, 59% yield. ^1^H NMR (400 MHz, CDCl_3_) δ 7.82 (d, *J* = 7.32 Hz, 2H), 7.70–7.50 (m, 3H), 7.41–7.37 (m, 2H), 7.31–7.27 (m, 1H), 6.34 (s, 1H), 2.48 (s, 3H); ^13^C NMR (100 MHz, CDCl_3_) δ 167.27, 144.05, 140.82, 138.76, 134.48, 130.37, 130.19, 129.63, 125.64, 125.28, 123.41, 119.76, 107.29, 21.51; HRMS (ESI) Calculated for C_16_H_12_O_2_[M+H]^+^: 237.0910, found 237.0913; FT-IR (Neat, cm^-1^): 2922, 1765, 1660, 1604, 1510, 1471, 1269, 856, 814.

**(Z)-3-(4-chlorobenzylidene)isobenzofuran-1(3H)-one (28c)^1^**: White solid, m.p. 142-146˚C, 70% yield. ^1^H NMR (400 MHz, CDCl_3_) δ 7.93 (d, *J* = 7.6 Hz, 1H), 7.78–7.70 (m, 4H), 7.57–7.53 (m, 1H), 7.38–7.34 (m, 2H), 6.36 (s, 1H); ^13^C NMR (100 MHz, CDCl_3_) δ 166.93, 145.00, 140.46, 134.70, 134.30, 131.69, 131.33, 130.08, 129.05, 125.79, 123.49, 119.93, 105.83 ppm; HRMS (ESI) Calculated for C_15_H_9_ClO_2_ [M+H]^+^: 257.0364, found 257.0366; FT-IR (Neat, cm^-1^): 1790, 1655, 1488, 1470, 1268, 686.

**(Z)-3-(4-bromobenzylidene)-isobenzofuran-1(3H)-one (28d)^1^**: White solid, m.p. 174-176˚C, 67% yield. ^1^H NMR (400 MHz, CDCl_3_) δ 7.94 (d, *J* = 7.6 Hz, 1H), 7.78–7.69 (m, 4H), 7.58–7.51 (m, 3H), 6.35 (s, 1H); ^13^C NMR (100 MHz, CDCl_3_) δ 166.87, 145.13, 140.46, 134.69, 132.12, 132.04, 131.56, 130.13, 125.80, 123.53, 122.63, 119.95, 105.85; HRMS (ESI) Calculated for C_15_H_9_BrO_2_ [M+H]^+^: 300.9859, found 300.9860; FT-IR (Neat, cm^-1^) 1791, 1631, 1487, 1470, 1269, 515.

**(Z)-3-(4-methoxybenzylidene)-isobenzofuran-1(3H)-one** (**28e**)^1^: Greenish blue solid, m.p. 136-140˚C, 70% yield. ^1^H NMR (400 MHz, CDCl_3_) δ 7.92 (d, *J* = 7.0 Hz, 1H), 7.81–7.67 (m, 4H), 7.52–7.48 m, 1H), 6.95-6.92 (m, 2H), 6.38 (s, 1H), 3.84 (s, 3H); ^13^C NMR (100 MHz, CDCl_3_) δ 167.36, 159.89, 143.17, 140.89, 134.44, 131.79, 129.38, 125.97, 125.64, 123.21, 119.60, 114.38, 107.04, 55.43; HRMS (ESI) Calculated for C_16_H_12_O_3_ [M+H]^+^: 253.0859, found 253.0860; FT-IR (Neat, cm^-1^): 2927, 2844, 1780, 1650, 1601, 1511, 1471, 1258.

**(Z)-3-(3-methoxybenzylidene) isobenzofuran-1(3H)-one** (**28f**)^1^: White solid, m.p. 118-120˚C, 71% yield. ^1^H NMR (400 MHz, CDCl3) δ 7.93 (d, *J* = 8.4 Hz, 1H), 7.77–7.69 (m, 2H), 7.55–7.52 (m, 1H), 7.41–7.40 (m, 2H), 7.33–7.29 (m, 1H), 6.88-6.86 (m, 1H), 6.38 (s, 1H), 3.38 (s, 3H); ^13^C NMR (100 MHz, CDCl3) δ 167.04, 159.86, 144.82, 140.66, 134.57, 134.42, 129.92, 129.79, 125.70, 123.53, 122.91, 119.92, 115.09, 114.58, 107.58, 55.43; HRMS (ESI) Calculated for C_16_H_12_O_3_ [M+H]^+^: 253.0859, found 253.0862; FT-IR (Neat, cm^-1^): 2923, 2861, 1765, 1665, 1600, 1570, 1472, 1453, 1434, 1276, 1047, 970, 756, 685, 634, 476.

**(Z)-3-benzylidene-6-bromoisobenzofuran-1(3H)-one (28g**)^1^: White solid, m.p. 176-178˚C, 63% yield. ^1^H NMR (400 MHz, CDCl_3_) δ 8.06–8.05 (m, 1H), 7.83–7.81 (m, 3H), 7.64 (d, *J* = 8.2 Hz, 1H), 7.43–7.39 (m, 2H), 7.35–7.31(m, 1H), 6.42 (s, 1H); ^13^C NMR (100 MHz, CDCl_3_) δ 165.62, 145.96, 143.90, 139.31, 137.71, 132.83, 130.30, 128.95, 128.60, 125.19, 123.78, 121.32, 108.08; HRMS (ESI) Calculated for C_15_H_9_BrO_2_ [M+H]^+^: 300.9859, found 300.9861; FT-IR (Neat, cm^-1^): 1773, 1665, 1491, 1461, 1250, 522.

**(Z)-6-bromo-3-(4-methylbenzylidene) isobenzofuran-1(3H)-one** (**28h**)^1^: Greenish white solid, m.p. 162-164˚C, 46% yield. ^1^H NMR (400 MHz, CDCl_3_) δ 8.05–8.04 (m, 1H), 7.80 (dd, *J* = 8.2, 1.7 Hz, 1H), 7.72 (d, *J* = 8.1 Hz, 2H), 7.62 (d, *J* = 8.3 Hz, 1H), 7.21 (d, *J* = 8.0 Hz, 2H), 6.40 (s, 1H), 2.37 (s, 3H); ^13^C NMR (100 MHz, CDCl_3_) δ 165.75, 143.28, 139.43, 139.19, 137.62, 130.29, 130.05, 129.72, 128.55, 125.08, 123.47, 121.19, 108.23, 21.57; HRMS (ESI) Calculated for C_16_H_11_BrO_2_ [M+H]^+^: 315.0015, found 315.0013; FT-IR (Neat, cm^-1^): 2917, 2830, 1764, 1668, 1606, 1509, 1459, 1286, 525.

**(Z)-6-bromo-3-(4-chlorobenzylidene)-isobenzofuran-1(3H)-one** (**28i**)^1^: White solid, m.p. 194-198˚C, 61% yield. ^1^H NMR (400 MHz, CDCl_3_) δ 8.06–8.05 (m, 1H), 7.84–7.81 (m, 1H), 7.77–7.74 (m, 2H), 7.64–7.61 (m, 1H), 7.38–7.35 (m, 2H), 6.36 (s, 1H); ^13^C NMR (100 MHz, CDCl_3_) δ 165.37, 144.23, 139.06, 137.83, 134.68, 131.42, 131.36, 129.18, 128.70, 125.18, 124.05, 121.34, 106.71; HRMS (ESI) Calculated for C_15_H_8_BrClO_2_ [M+H]^+^: 334.9469, found 334.9470; FT-IR (Neat, cm^-1^): 1779, 1684, 1491, 1463, 1249, 622, 520.

**(Z)-6-bromo-3-(4-bromobenzylidene) isobenzofuran-1(3H)-one** (**28j**)^1^: Greenish white, m.p. 210-212˚C, 62% yield. ^1^H NMR (400 MHz, CDCl_3_) δ 8.07–8.06 (m, 1H), 7.85–7.82 (m, 1H), 7.70–7.68 (m, 2H), 7.63 (d, *J* = 8.2 Hz, 1H), 7.53 (d, *J* = 8.6 Hz, 2H), 6.35 (s, 1H); ^13^C NMR (100 MHz, CDCl_3_) δ 165.35, 144.46, 139.06, 137.85, 132.15, 131.78, 131.64, 128.73, 125.21, 124.09, 123.04, 121.35, 106.77; HRMS (ESI) Calculated for C_15_H_8_Br_2_O_2_ [M+H]^+^: 378.8964, found 378.8962; FT-IR (Neat, cm^-1^): 1767, 1667, 1581, 1459, 1408, 1246, 1065, 972, 823, 518.

**(Z)-3-benzylidene-6-methylisobenzofuran-1(3H)-one** (**28m**)^1^: White solid, m.p. 140-144˚C, 63% yield. ^1^H NMR (400 MHz, CDCl_3_) δ 7.82 (d, *J* = 7.3 Hz, 2H), 7.70 (s, 1H), 7.64 (d, *J* = 8.0 Hz, 1H), 7.52 (d, *J* = 8 Hz, 1H), 7.41–7.37 (m, 2H), 7.30–7.27 (m, 1H), 6.37 (s,1H), 2.47 (s, 3H); ^13^C NMR (100 MHz, CDCl_3_) δ 167.32, 144.82, 140.56, 138.33, 135.90, 133.35, 130.09, 128.83, 128.29, 125.32, 123.75, 119.71, 106.33, 21.65; HRMS (ESI) Calculated for C_16_H_12_O_2_ [M+H]^+^: 237.0910, found 237.0911; FT-IR (Neat, cm^-1^): 2920, 2852, 1776, 1600, 1492, 1447, 1272.

**(Z)-6-methyl-3-(4-methylbenzylidene) isobenzofuran-1(3H)-one** (**28n**)^1^: White solid, m.p. 170-172˚C, 60% yield. ^1^H NMR (400 MHz, CDCl_3_) δ 7.72–7.69 (m, 3H), 7.62 (d, *J* = 8.0 Hz, 1H), 7.51 ( d, *J* = 8.0 Hz, 1H), 7.20 (d, *J* = 8.0 Hz, 2H), 6.31(s, 1H), 2.47 (s, 3H), 2.36 (s, 3H); ^13^C NMR (100 MHz, CDCl_3_) δ 167.46, 144.49, 140.27, 138.49, 138.45, 135.82, 130.54, 130.06, 129.63, 125.47, 123.64, 119.54, 106.47, 21.61, 21.49; HRMS (ESI) Calculated for C_17_H_14_O_2_ [M+H]^+^: 251.1067, found 251.1068; FT-IR (Neat, cm^-1^):2921, 1762, 1663, 1487, 1270.

**(Z)-3-(4-chlorobenzylidene)-6-methylisobenzofuran-1(3H)-one** (**28o**)^1^: White solid, m.p.182-184˚C, 60% yield. ^1^H NMR (400 MHz, CDCl_3_) δ 7.74–7.69 (m, 3H), 7.63 (d, *J* = 7.9 Hz, 1H), 7.52 (d, *J* = 8.0 Hz, 1H), 7.35–7.32 (m, 2H), 6.27 (s, 1H), 2.48 (s, 3H); ^13^C NMR (100 MHz, CDCl_3_) δ 167.04, 145.15, 140.84, 138.08, 135.97, 134.02, 131.88, 131.19, 128.99, 125.62, 123.75, 119.72, 104.98, 21.63; HRMS (ESI) Calculated for C_16_H_11_ClO_2_ [M+H]^+^: 271.0521, found 271.0520; FT-IR (Neat, cm^-1^): 2922, 2840, 1756, 1666, 1490, 1250, 675.

**(Z)-3-(4-methoxybenzylidene)-6-methylisobenzofuran-1(3H)-one** (**28p**)^1^: Greenish white solid, m.p. 152-156˚C, 67% yield. ^1^H NMR (400 MHz, CDCl_3_) δ 7.78 (d, *J* = 8.8 Hz, 2H), 7.69 (s, 1H), 7.62 (d, *J* = 8.0 Hz, 1H), 7.50 (d, *J* = 8.0 Hz, 1H), 6.93 (d, *J* = 8.8 Hz, 2H), 6.30 (s, 1H), 3.83 (s, 3H), 2.47 (s, 3H); ^13^C NMR (100 MHz, CDCl_3_) δ 167.53, 159.68, 143.34, 140.00, 138.52, 131.62, 126.75, 126.14, 125.54, 123.46, 119.41, 118.33, 106.20, 55.42, 21.60; HRMS (ESI) Calculated for C_17_H_13_O_3_ [M+H]^+^: 267.1016, found 267.1015; FT-IR (Neat, cm^-1^): 2927, 2840, 1761, 1663, 1603, 1509, 1255.

**(Z)-3-(3-methoxybenzylidene)-6-methylisobenzofuran-1(3H)-one** (**28q**)^1^: White solid, m.p. 128-130˚C, 80% yield. ^1^H NMR (400 MHz, CDCl3) δ 7.70 (s, 1H), 7.64 (d, *J* = 8.0 Hz, 1H), 7.52 ( d, *J* = 8.3 Hz, 1H), 7.40–7.39 (m, 2H), 7.32–7.28 (m, 1H), 6.87–6.84 (m, 1H), 6.31 (s, 1H), 3.85 (s, 3H), 2.48 (s, 3H); ^13^C NMR (100 MHz, CDCl3) δ 167.18, 159.84, 144.98, 140.61, 138.29, 135.88, 134.61, 129.74, 125.54, 123.80, 122.79, 119.72, 114.94, 114.37, 106.21, 55.42, 21.64; HRMS (ESI) Calculated for C_17_H_14_O_3_ [M+H]^+^: 267.1016, found 267.1017. FT-IR (Neat, cm^-1^): 3073, 3001, 2913, 2830, 1758, 1661, 1567, 1465, 1277, 969, 890, 779, 690, 494.

**(Z)-3-benzylidene-6-fluoroisobenzofuran-1(3H)-one** (**28r**)^1^: White solid, m.p. 172-174˚C, 61% yield. ^1^H NMR (400 MHz, CDCl_3_) δ 7.83 (d, *J* = 7.4 Hz, 2H), 7.77–7.74 (m, 1H), 7.58 (dd, *J* = 7.0 Hz, 2.2 Hz, 1H), 7.47–7.39 (m, 3H), 7.32 (t, *J* = 7.3 Hz, 1H), 6.38 (s, 1H); ^13^C NMR (100 MHz, CDCl_3_) δ 165.98, 164.78, (*J*_C-F_ = 251 Hz), 143.89, 136.71, 132.94, 130.16, 128.92, 128.68, 125.36, (*J*_C-F_ = 9 Hz), 123.00, (*J*_C-F_ = 25 Hz), 121.85, (*J*_C-F_ = 9 Hz), 111.87, (*J*_C-F_ = 24 Hz), 107.29; HRMS (ESI) Calculated for C_15_H_9_FO_2_ [M+H]^+^: 241.0659, found 241.0662; FT-IR (Neat, cm^-1^ ): 1763, 1668, 1610, 1478, 1256, 1063, 982, 919, 681, 529.

**(Z)-3-benzylidene-6-methoxyisobenzofuran-1(3H)-one** (**28t**)^1^: White solid, m.p. 146-150˚C, 72% yield. ^1^H NMR (400 MHz, CDCl_3_) δ 7.82–7.79 (m, 2H), 7.66 (d, *J* = 8.4 Hz, 1H), 7.41–7.37 (m, 2H), 7.33 (d, *J* = 2.2 Hz, 1H), 7.30–7.26 (m, 2H), 6.28 (s, 1H), 3.89 (s, 3H); ^13^C NMR (100 MHz, CDCl_3_) δ 167.25, 161.45, 144.68, 133.72, 133.44, 129.95, 128.83, 128.16, 125.06, 124.12, 121.18, 106.86, 105.73, 55.99; HRMS (ESI) Calculated for C_16_H_12_O_3_ [M+H]^+^: 253.0859, found 253.0861; FT-IR (Neat, cm^-1^): 2935, 2841, 1759, 1658, 1605, 1448, 1486, 1283, 1071, 975, 781, 687, 544.

**2. ^1^H NMR and ^13^C NMR Spectral Data of 28a-t**.


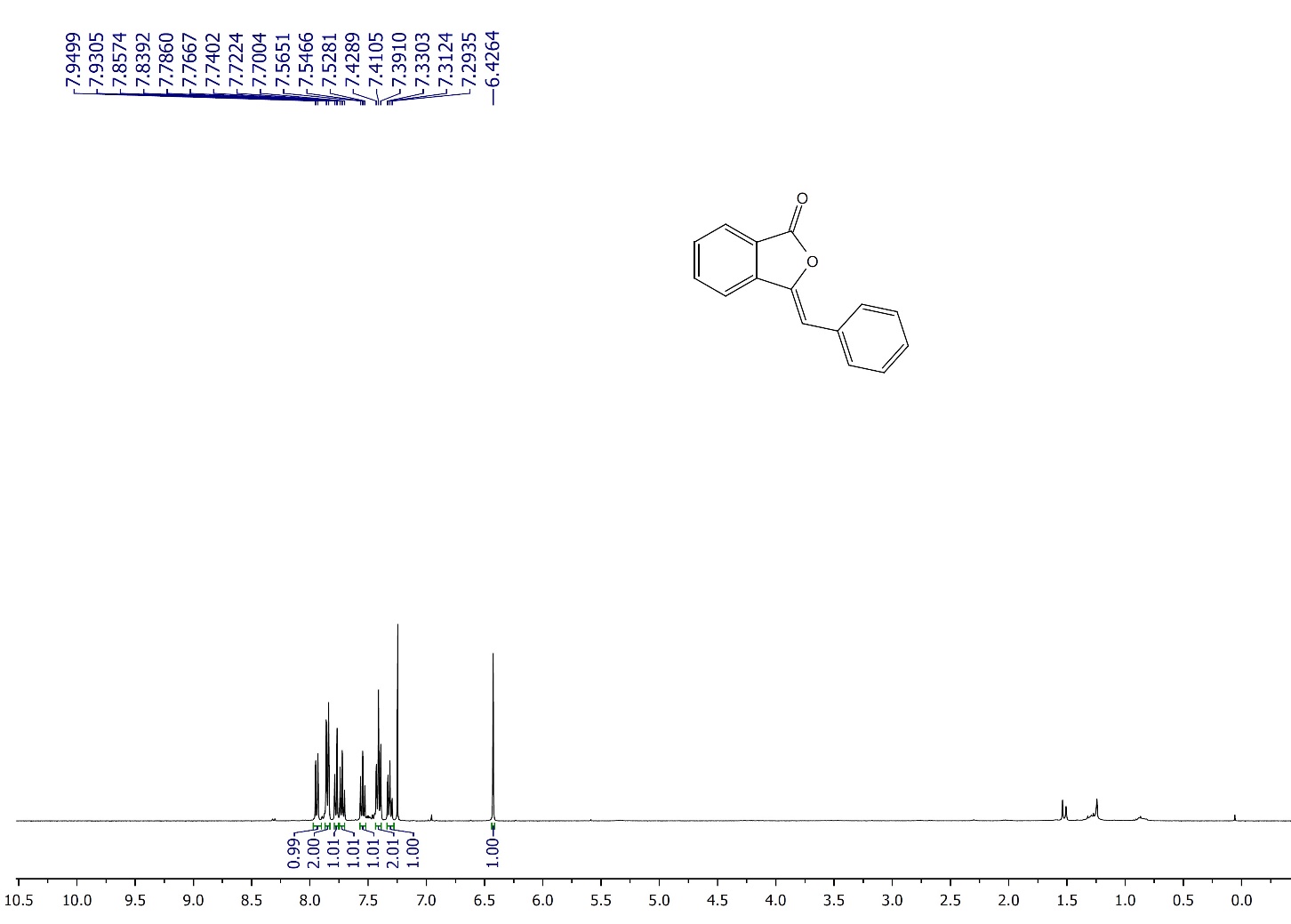


^1^H-NMR of **28a**.

**^
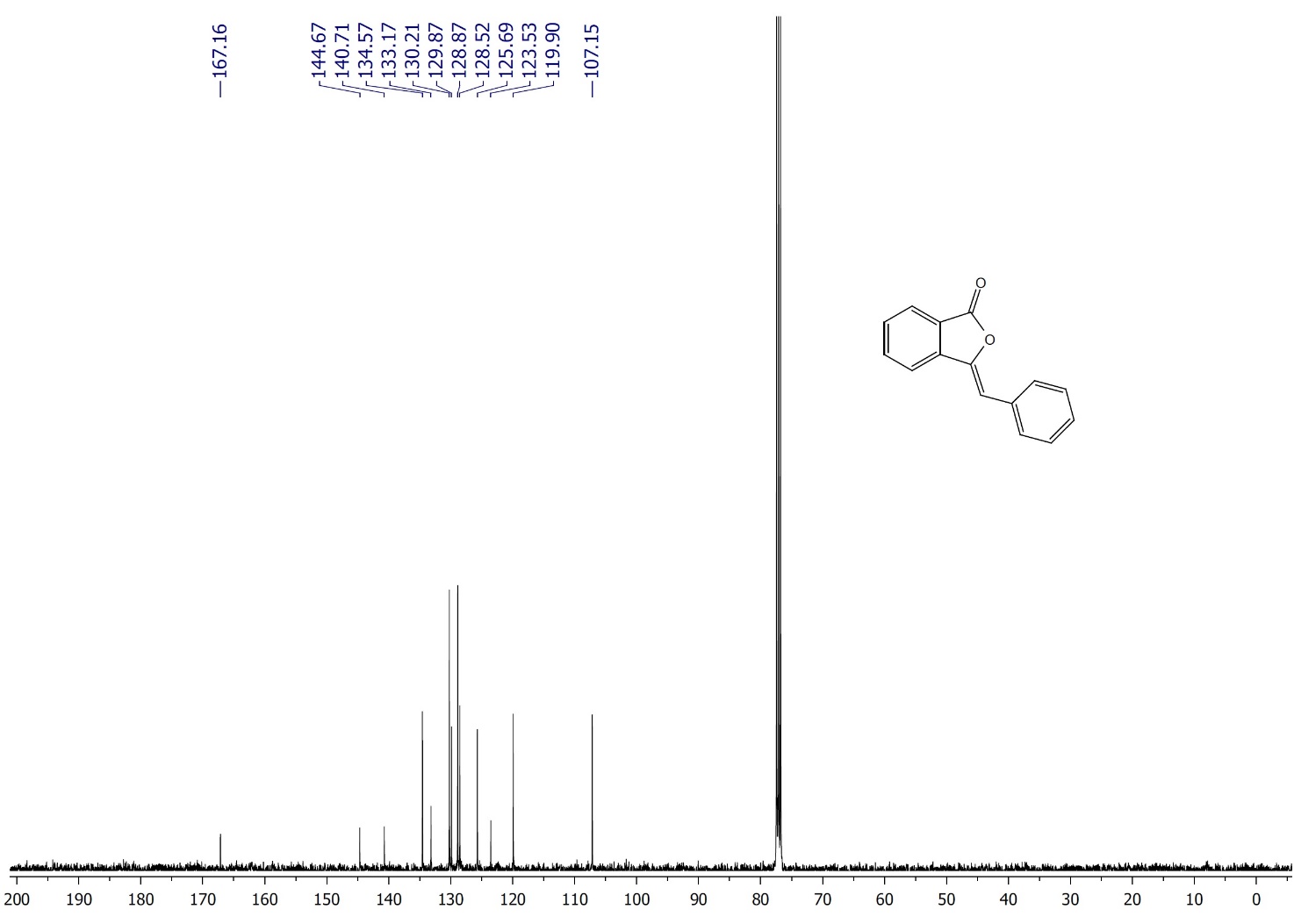
^**

^13^C-NMR of **28a**.


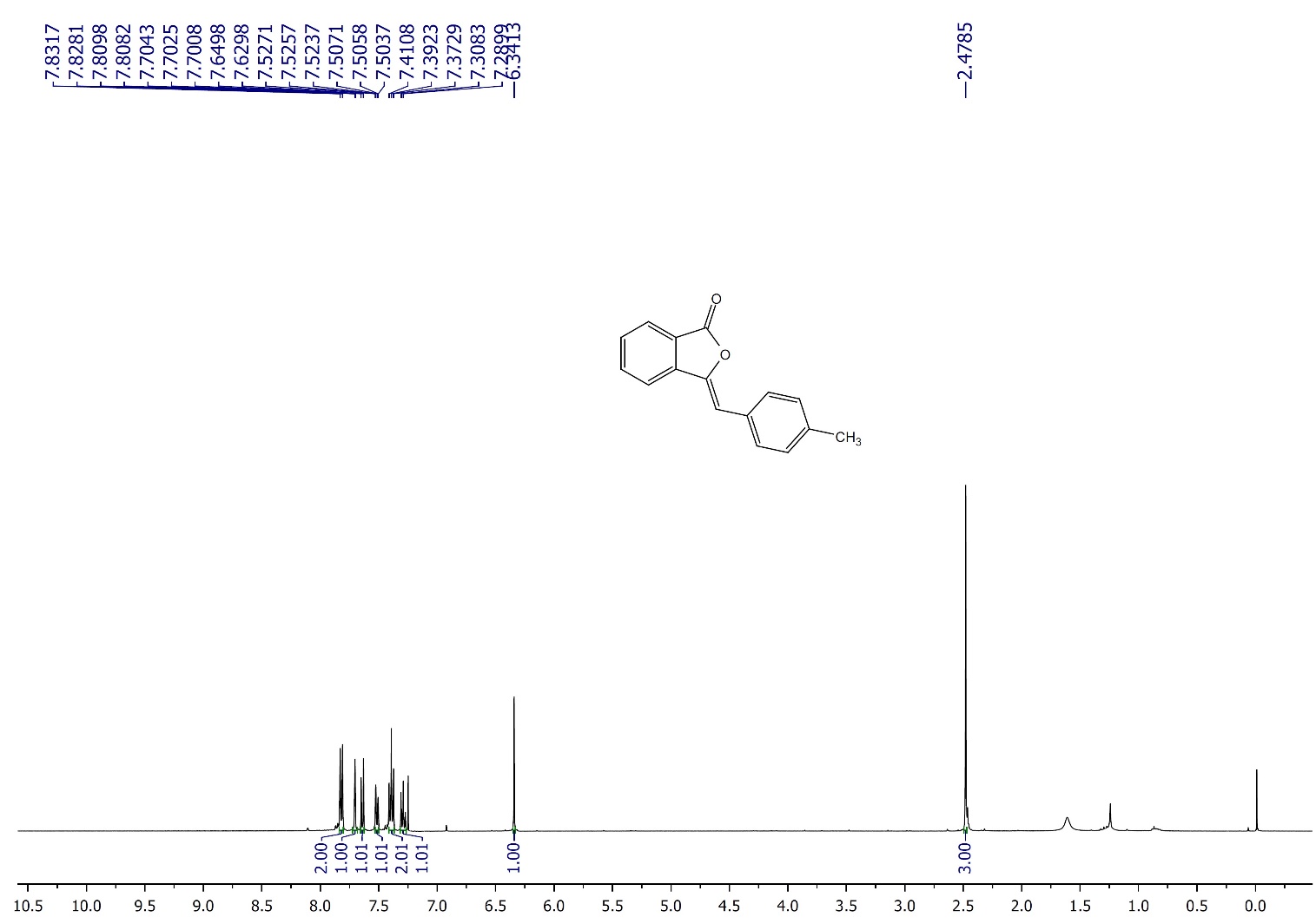


^1^H-NMR of **28b**.


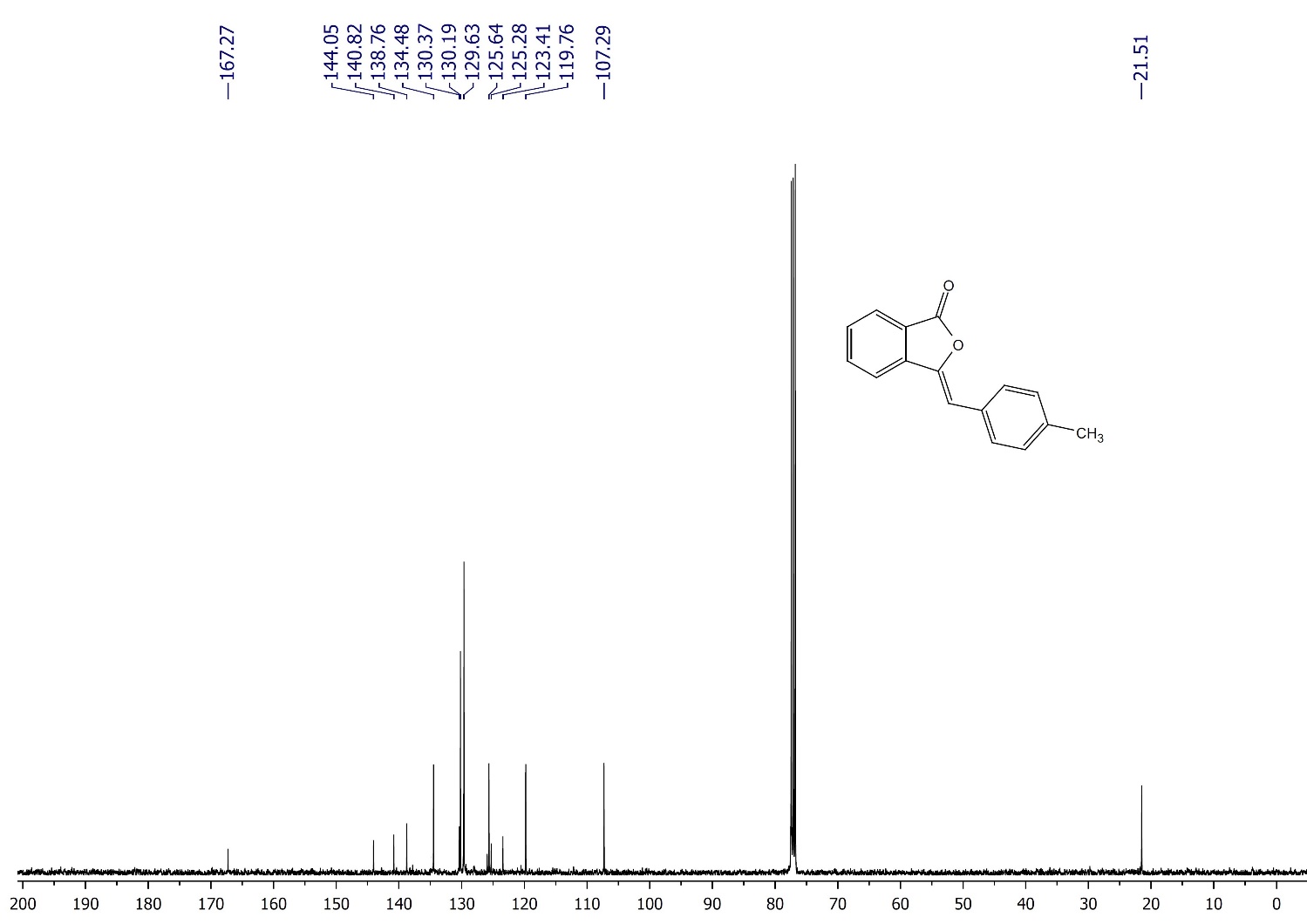


^13^C-NMR of **28b**.


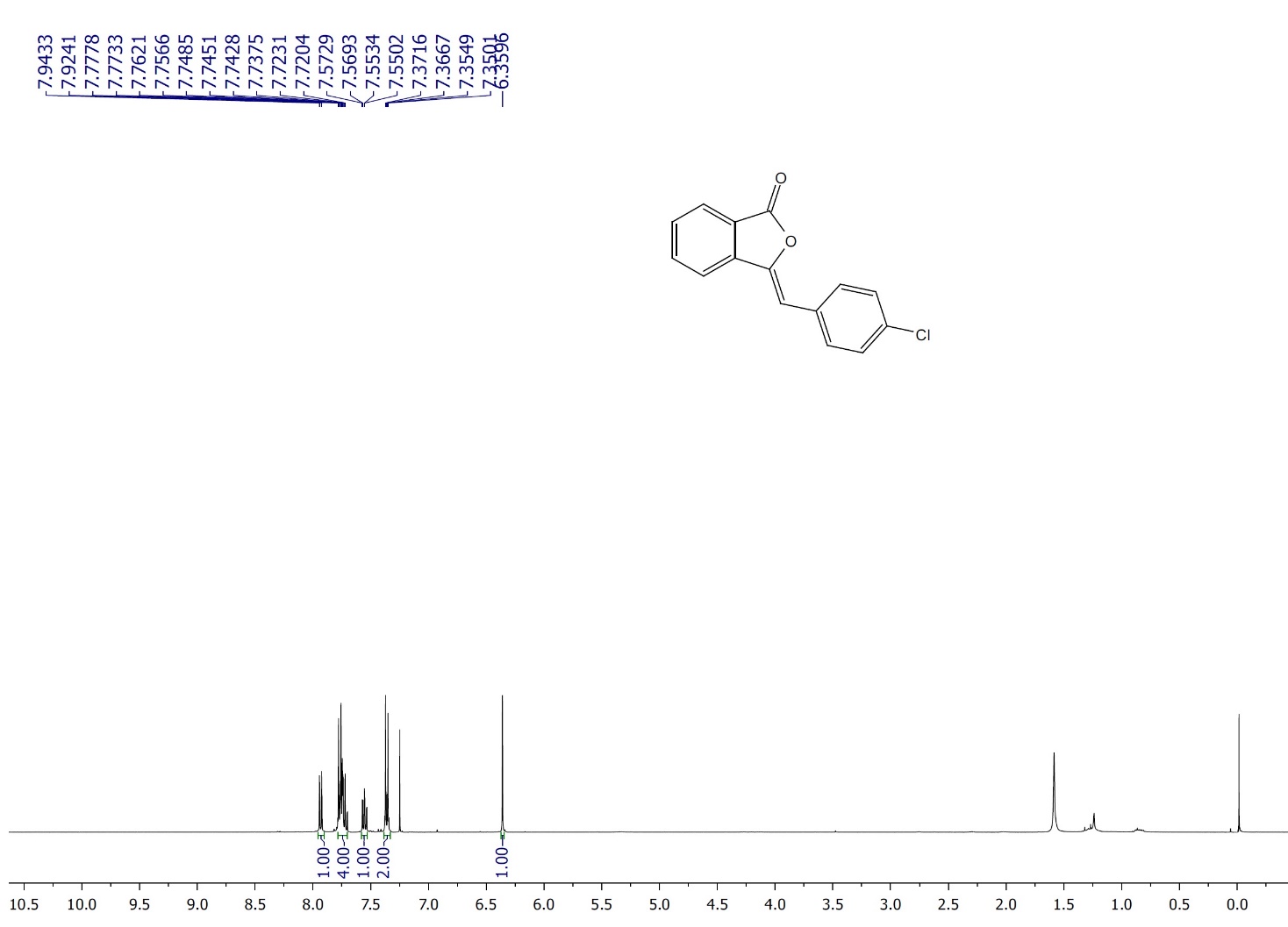


^1^H-NMR of **28c**.


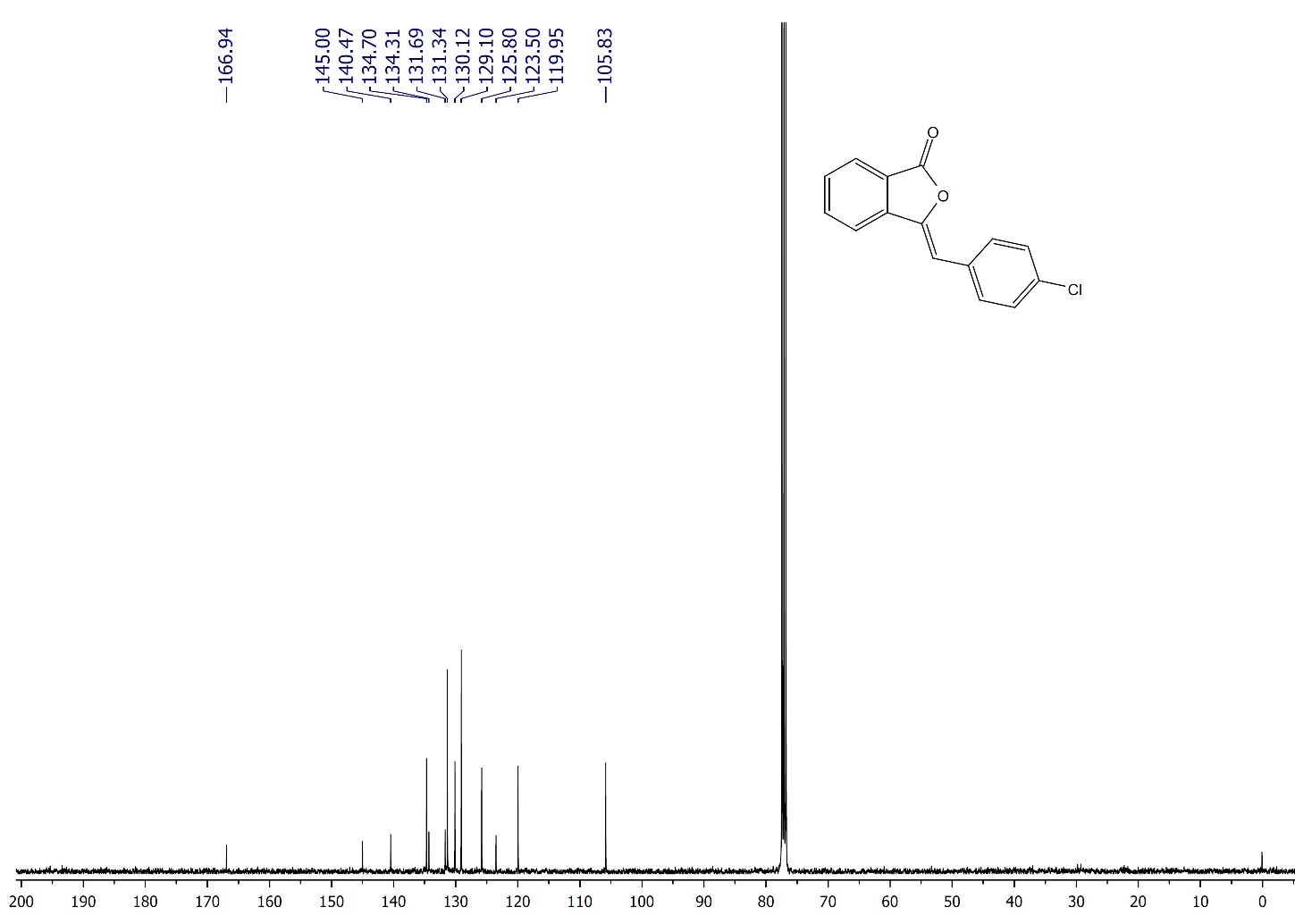


^13^C-NMR of **28c**.


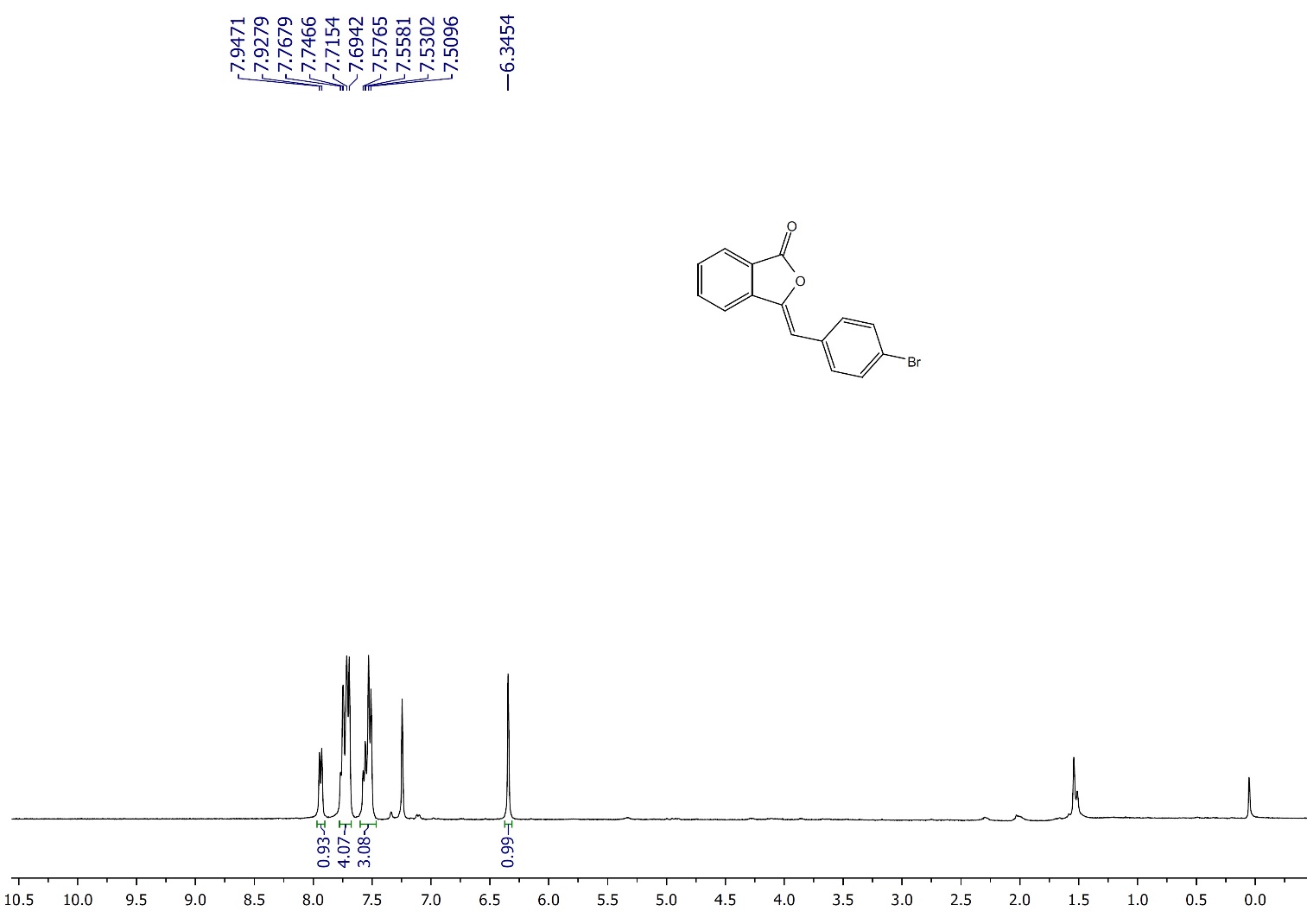


^1^H-NMR of **28d**.


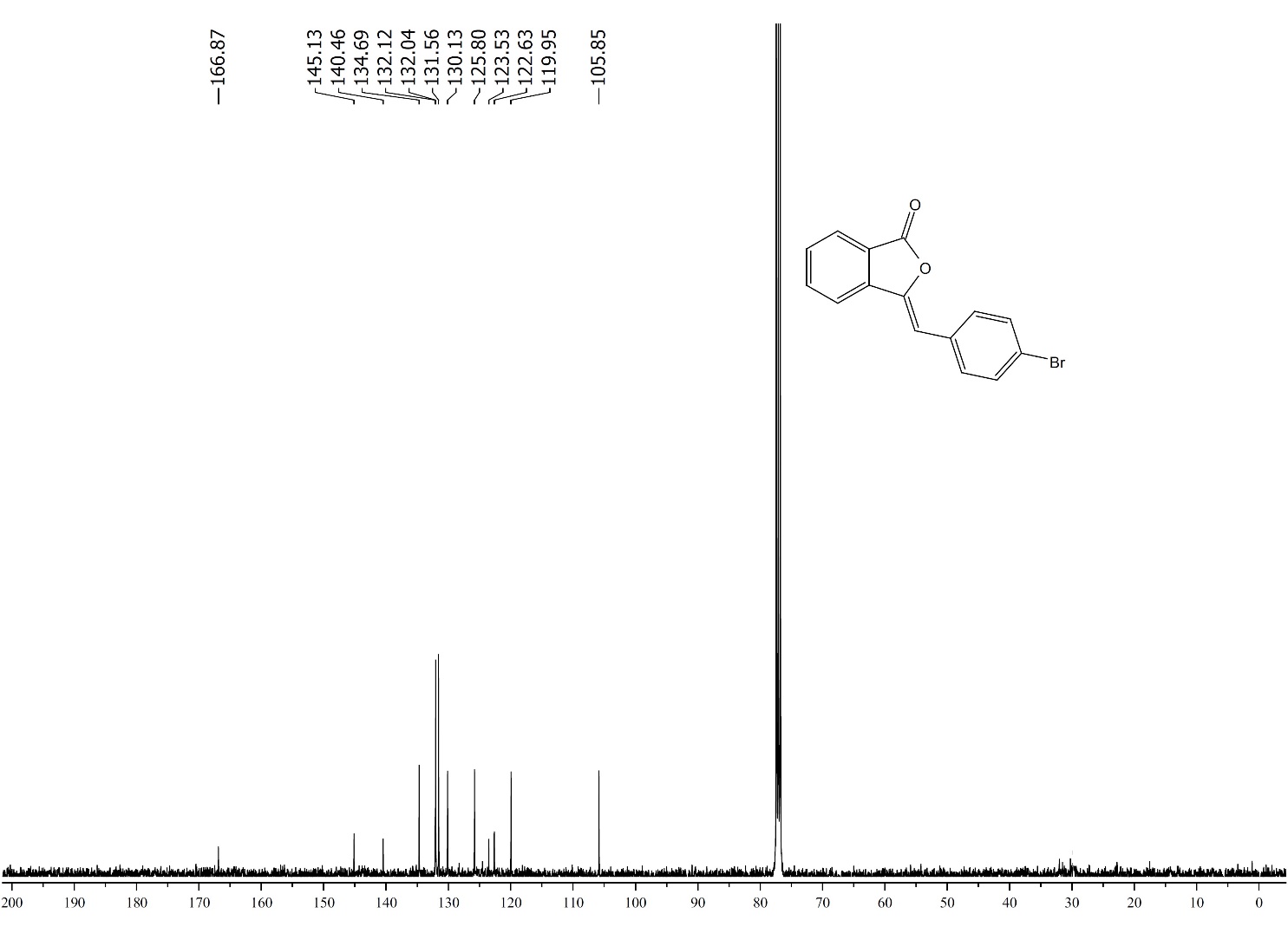


^13^C-NMR of **28d**.


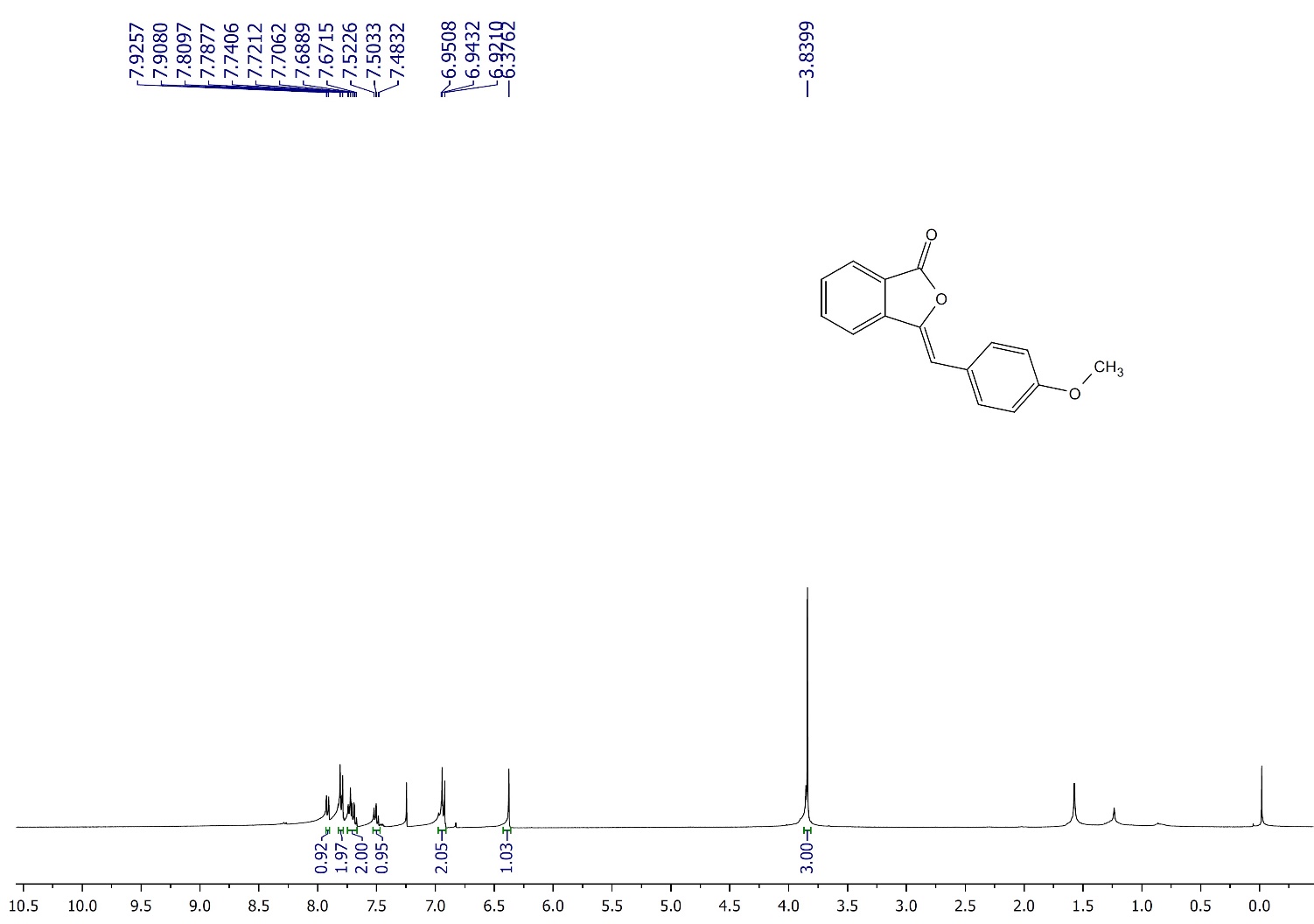


^1^H-NMR of **28e**.


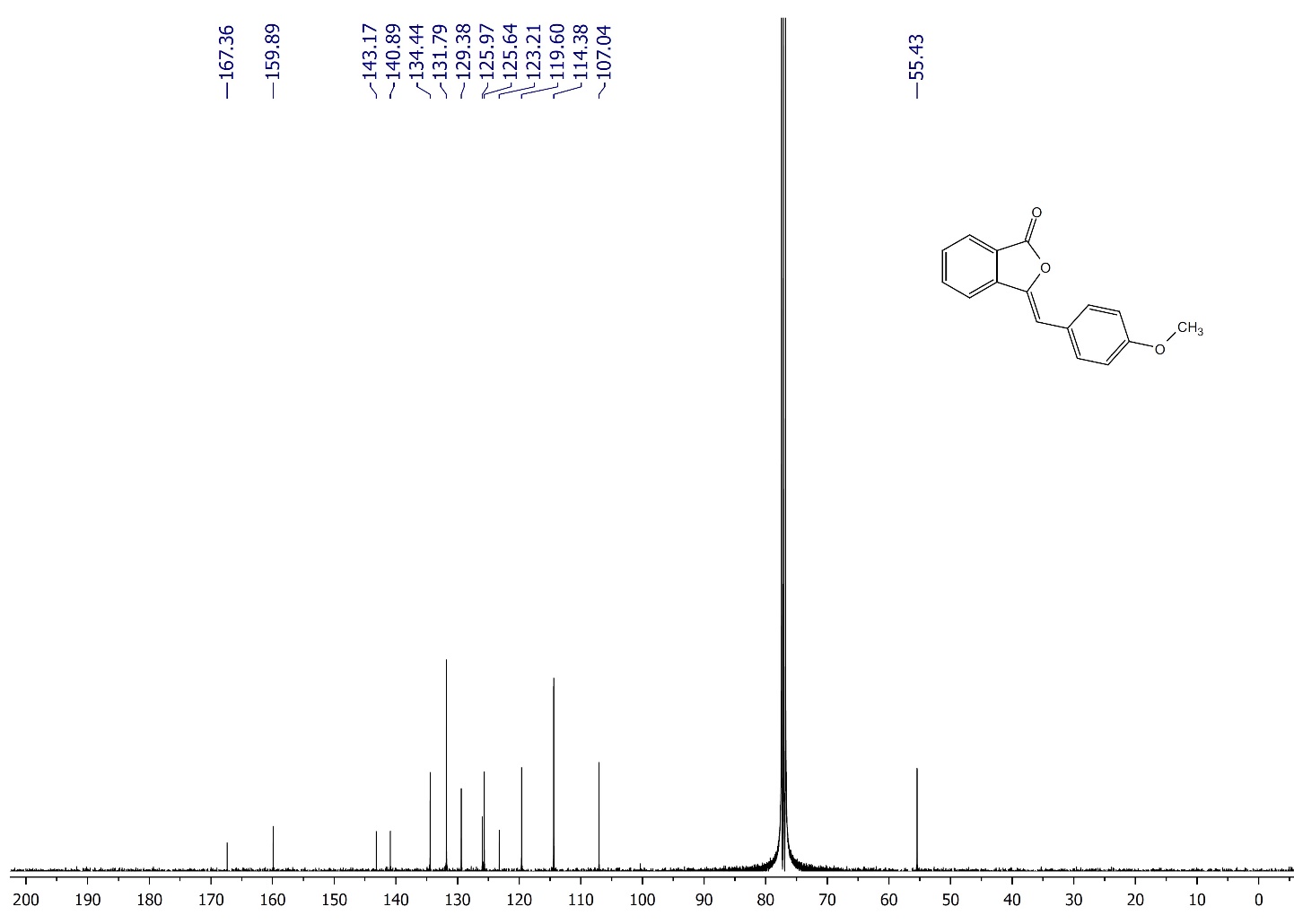


^13^C-NMR spectral of **28e**.


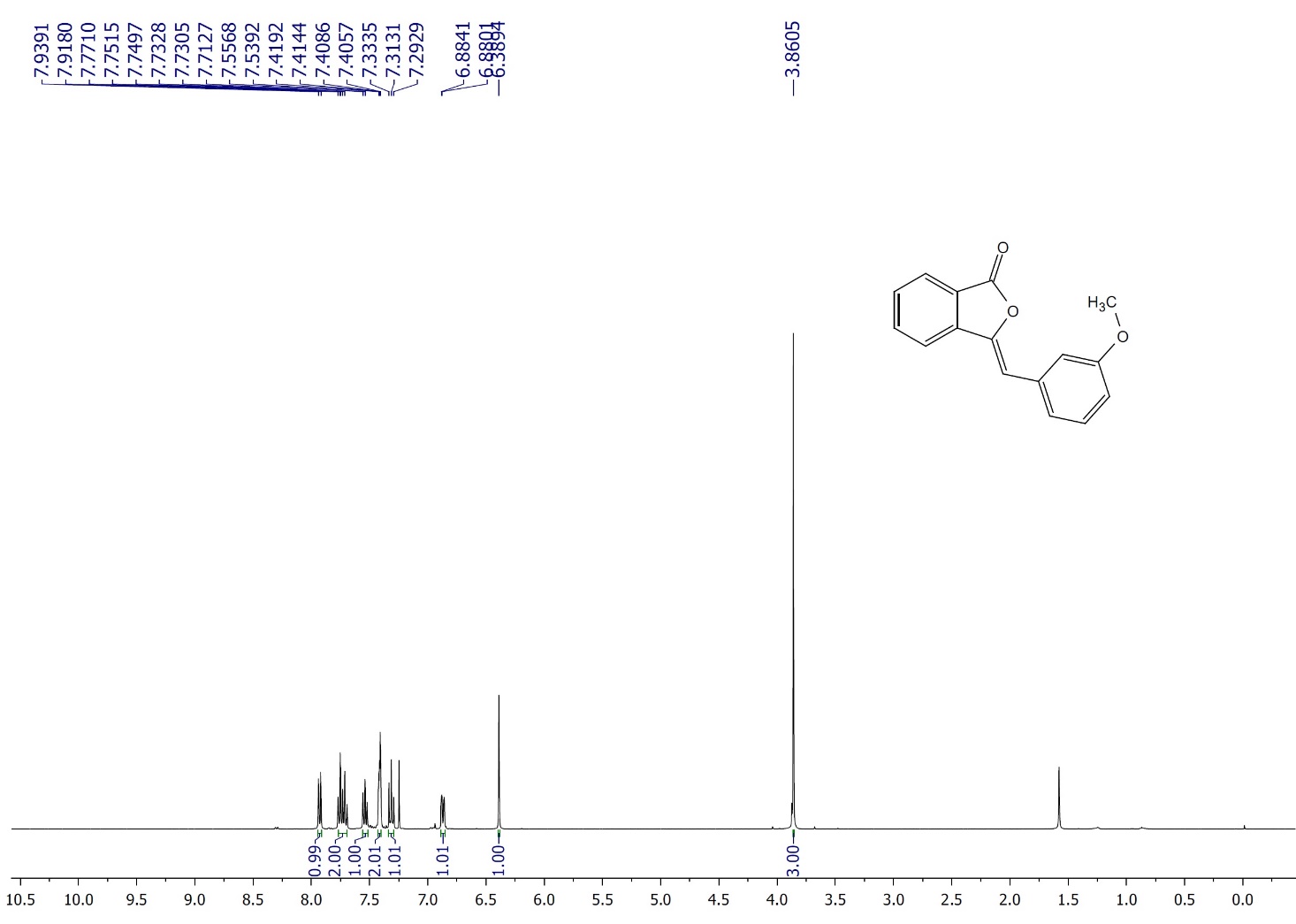


^1^H-NMR of **28f**.


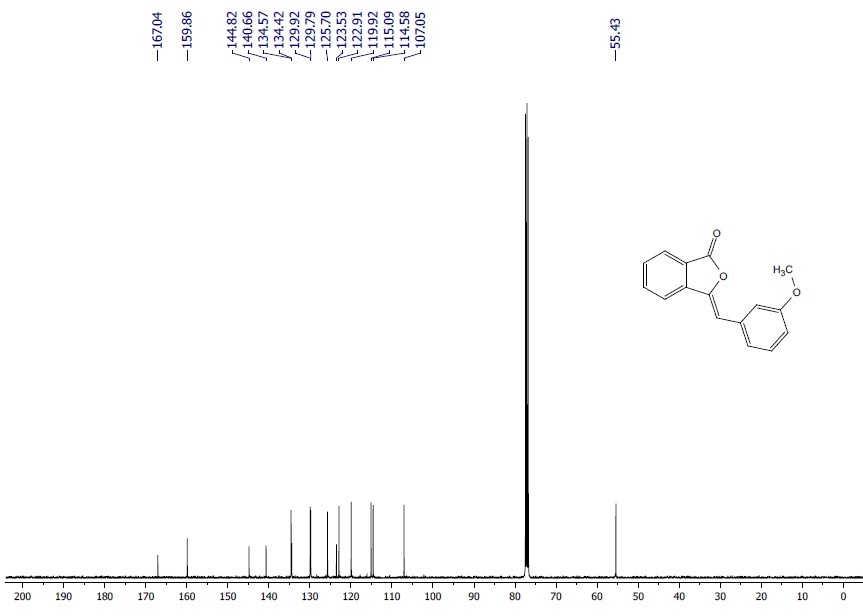


^13^C-NMR of **28f**.

**^
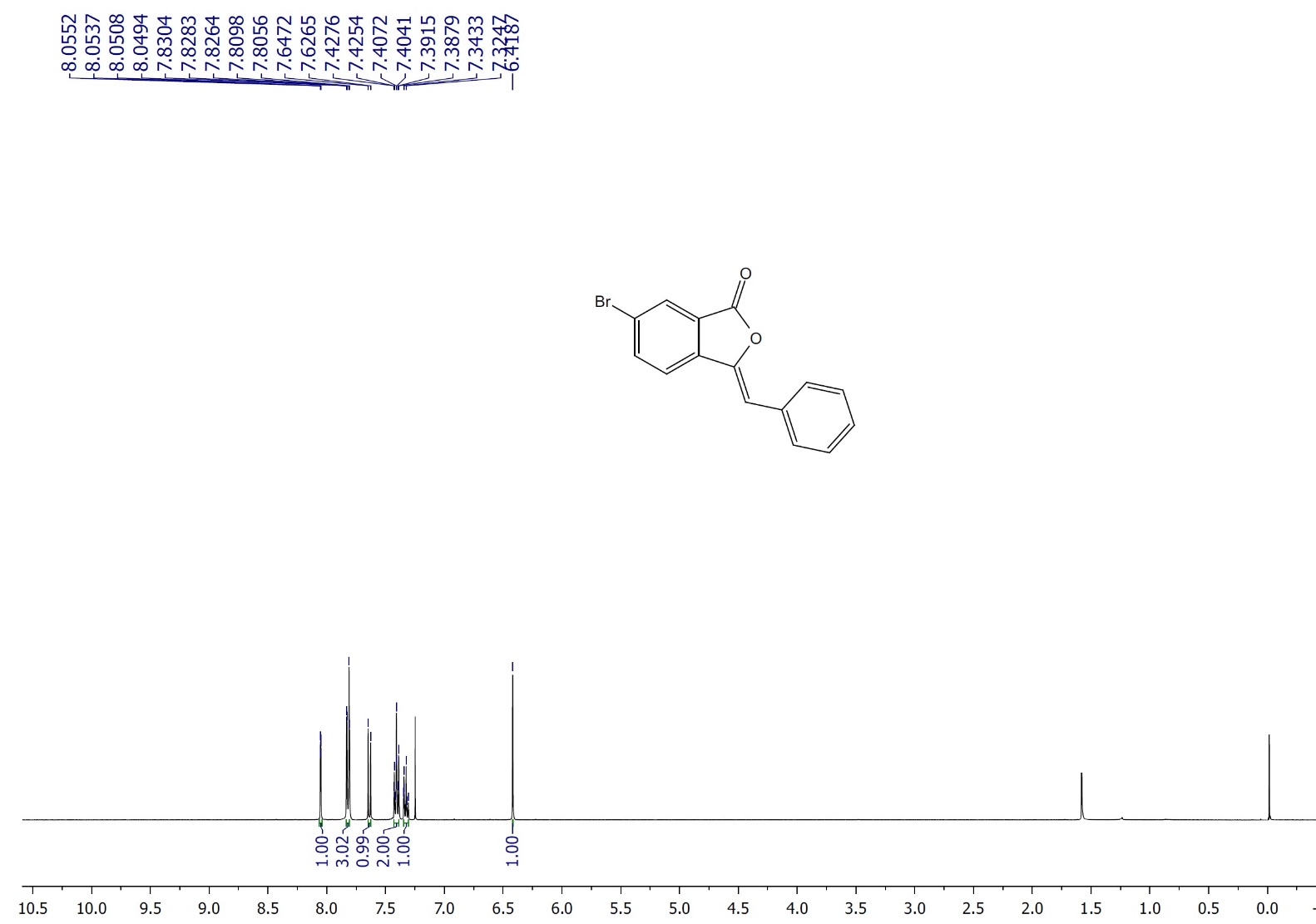
^**

^1^H-NMR of **28g**.


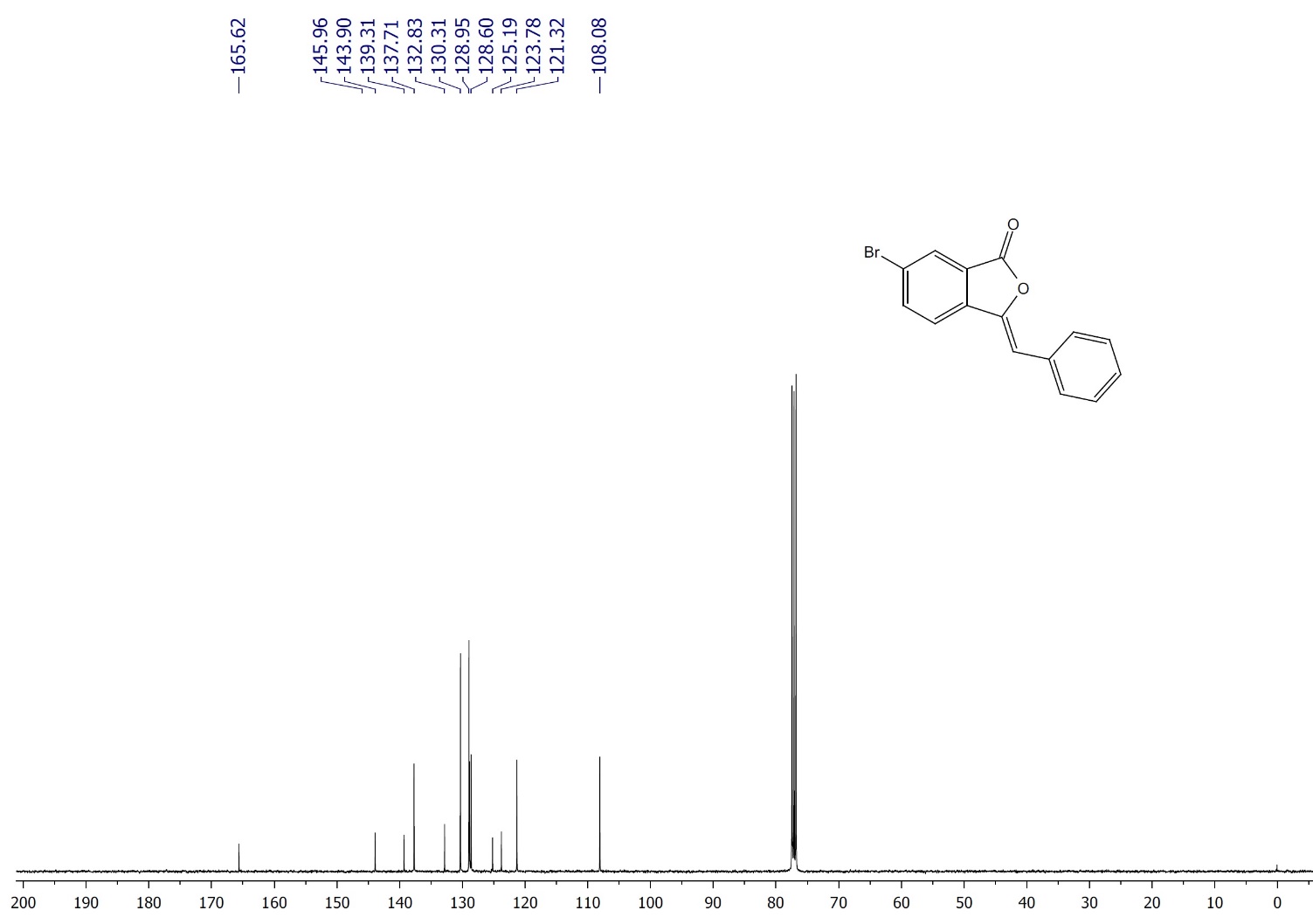


^13^C-NMR of **28g**.


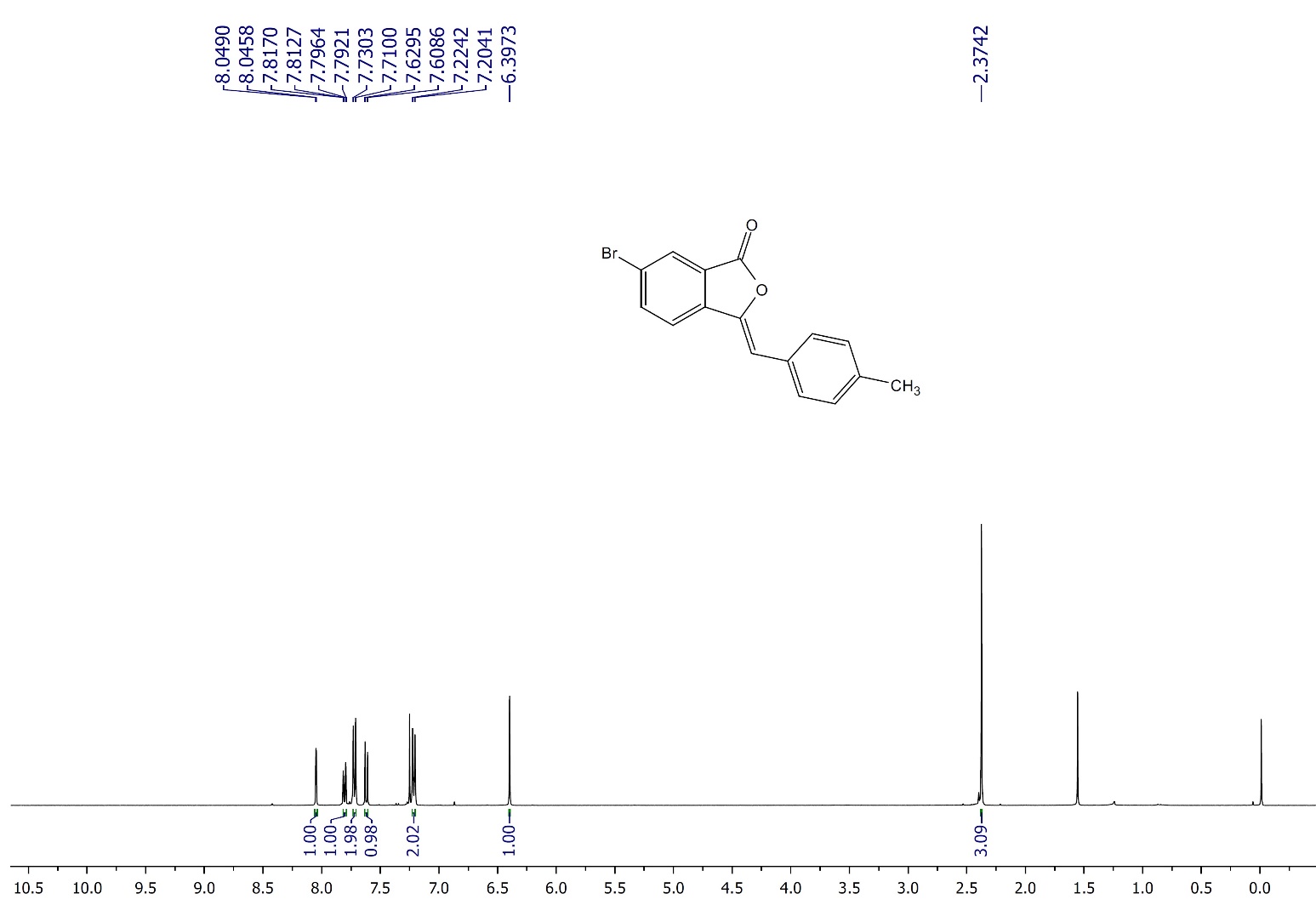


^1^H-NMR of **28h**.


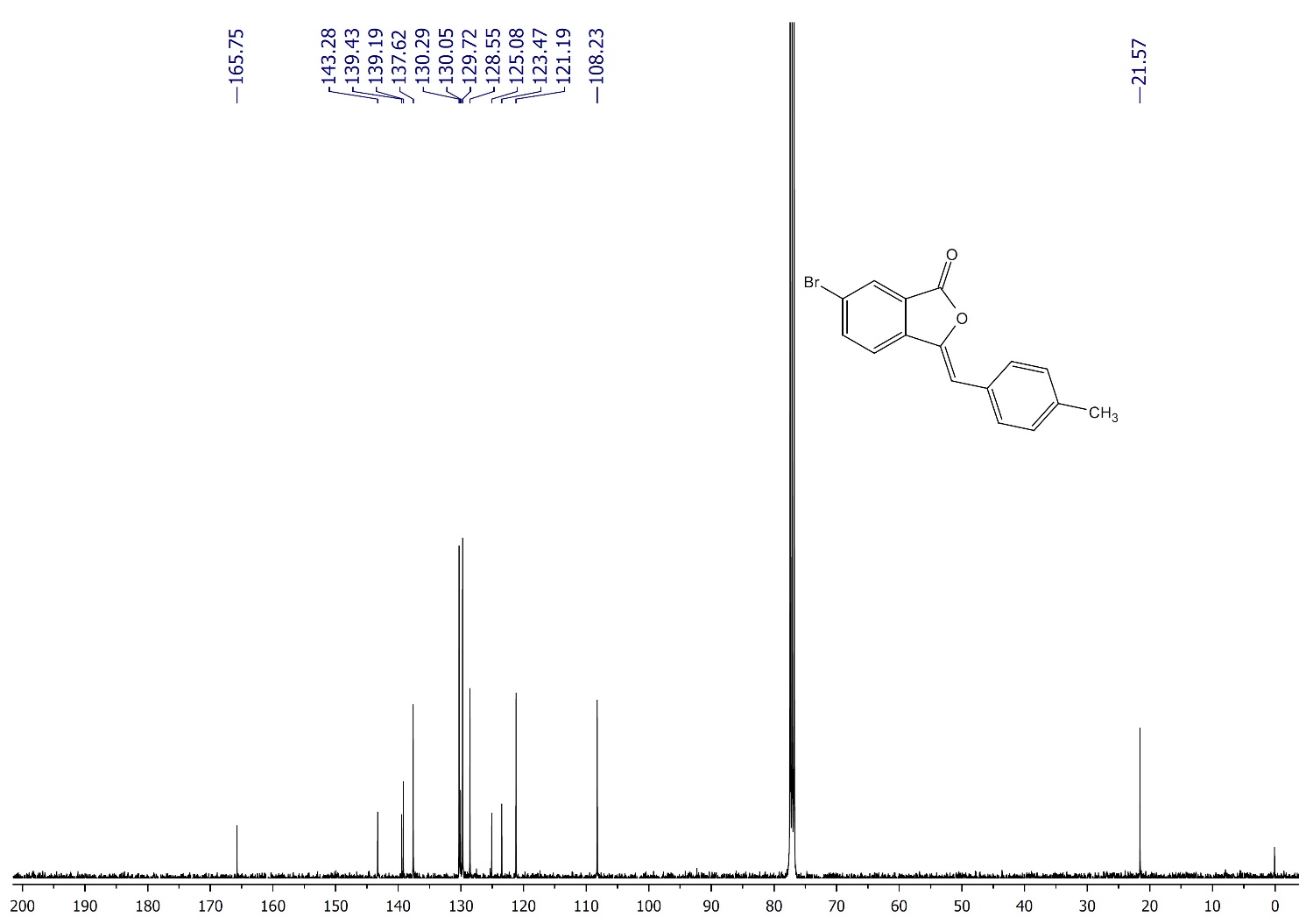


^13^C-NMR of **28h**.


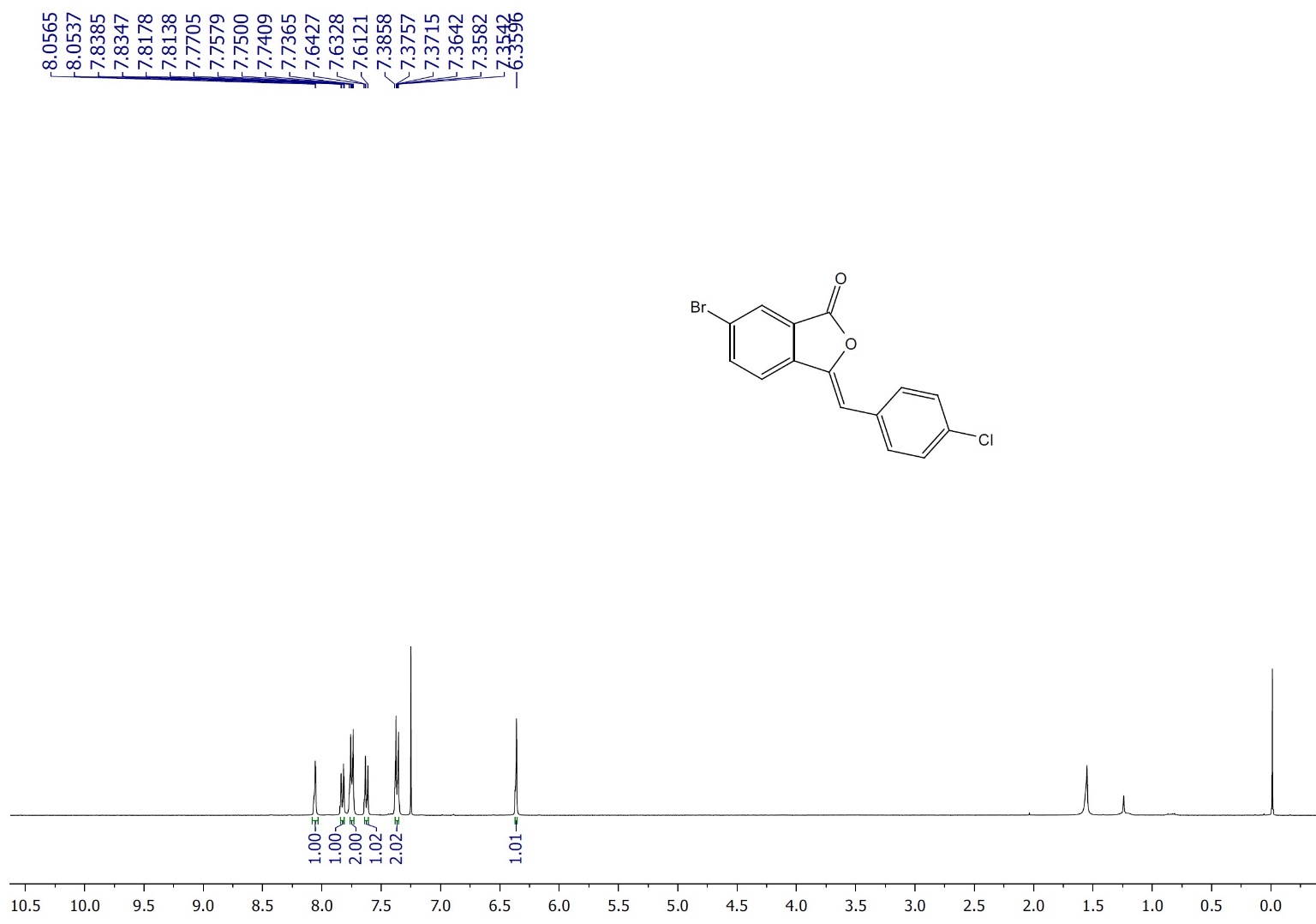


^1^H-NMR of **28i**.


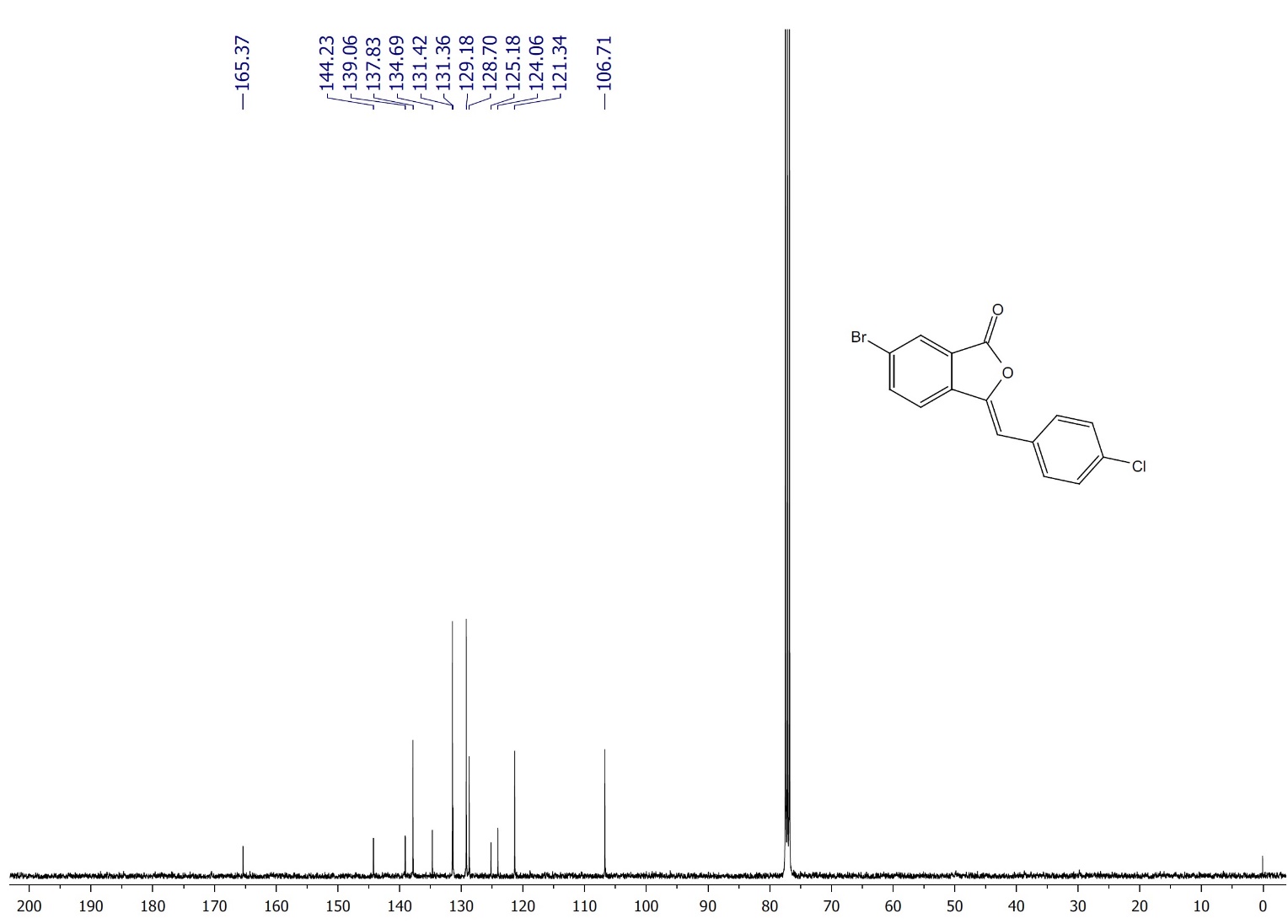


^13^C-NMR of **28i**.


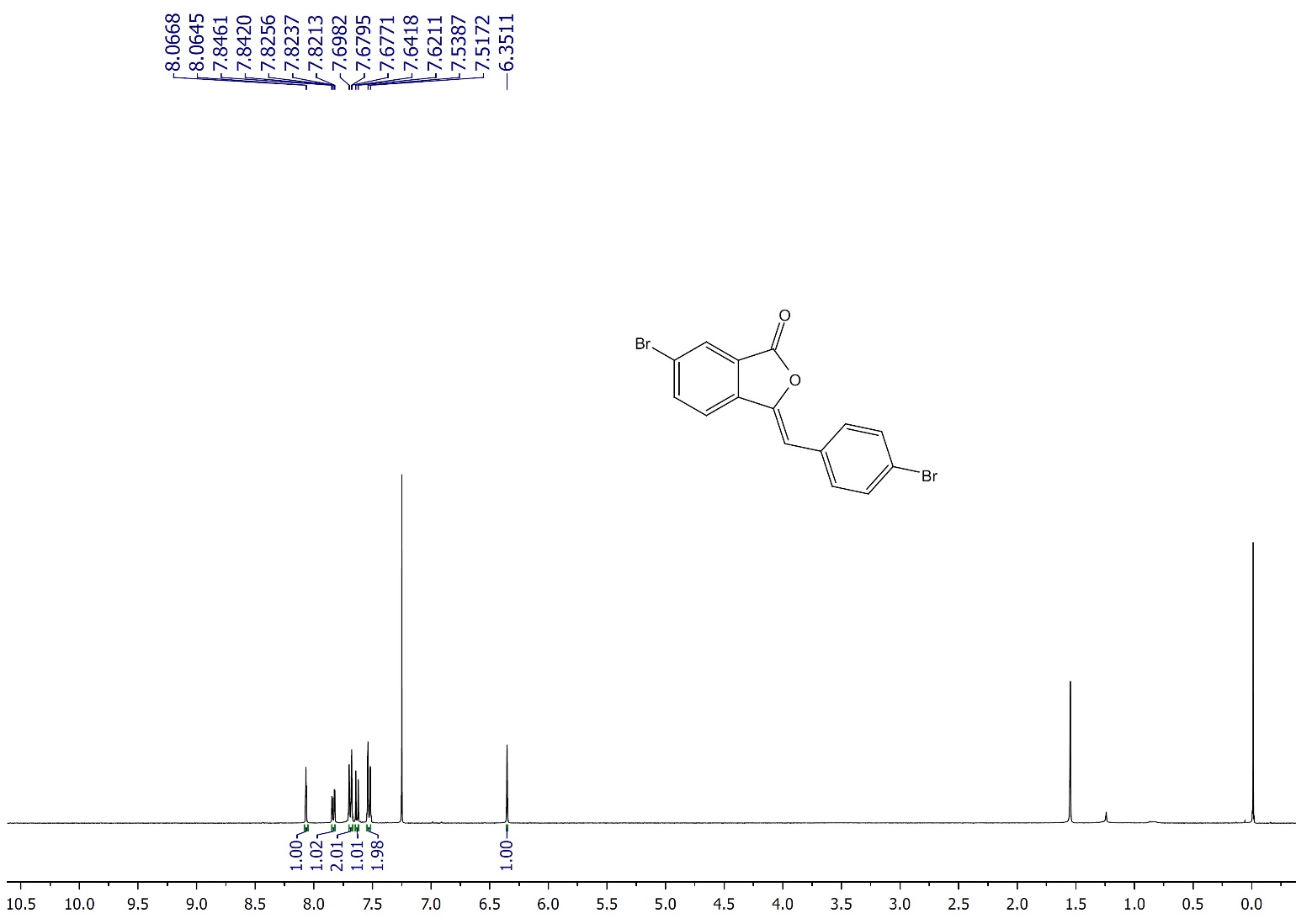


^1^H-NMR of **28j**.

**^
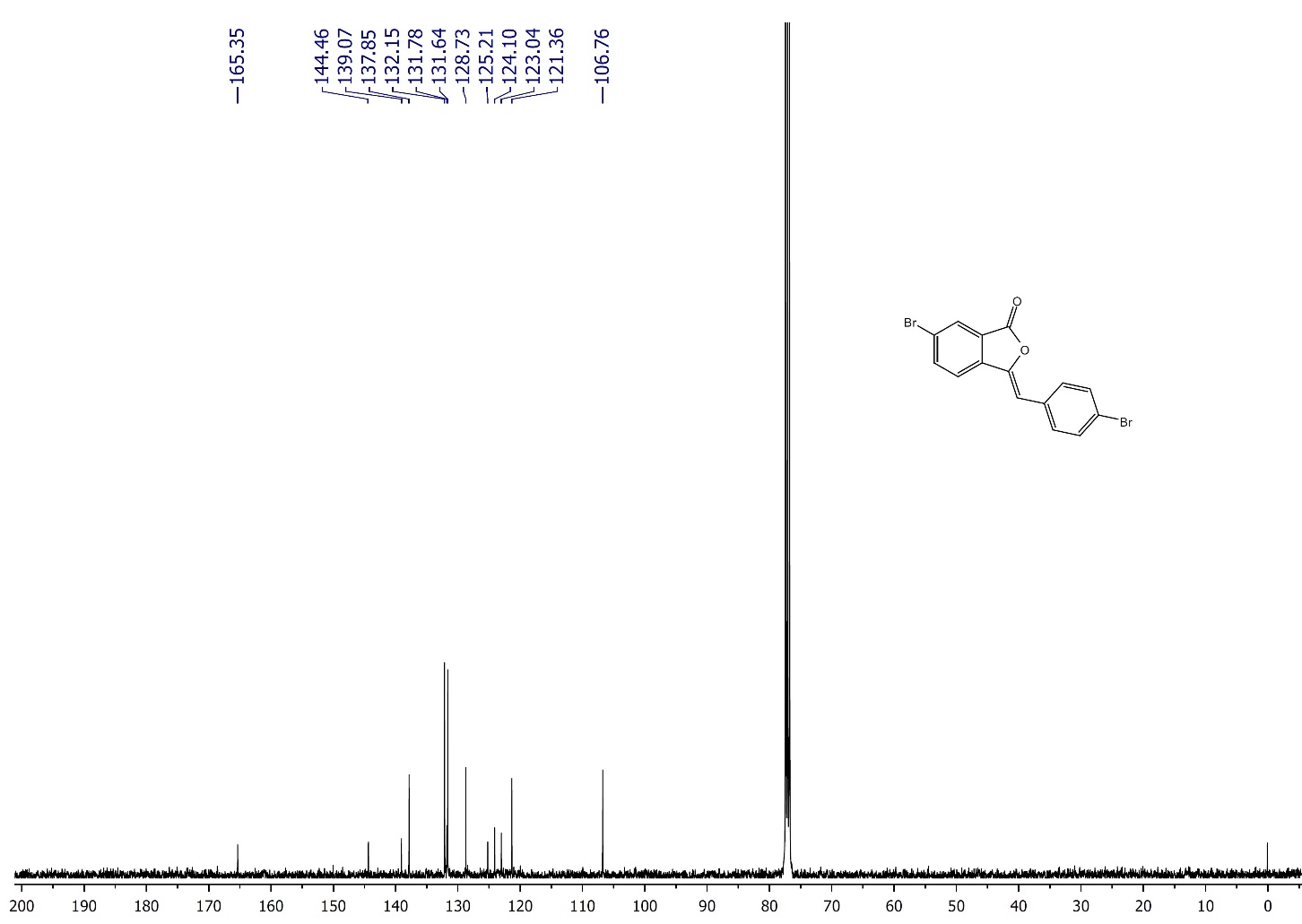
^**

^13^C-NMR of **28j**.


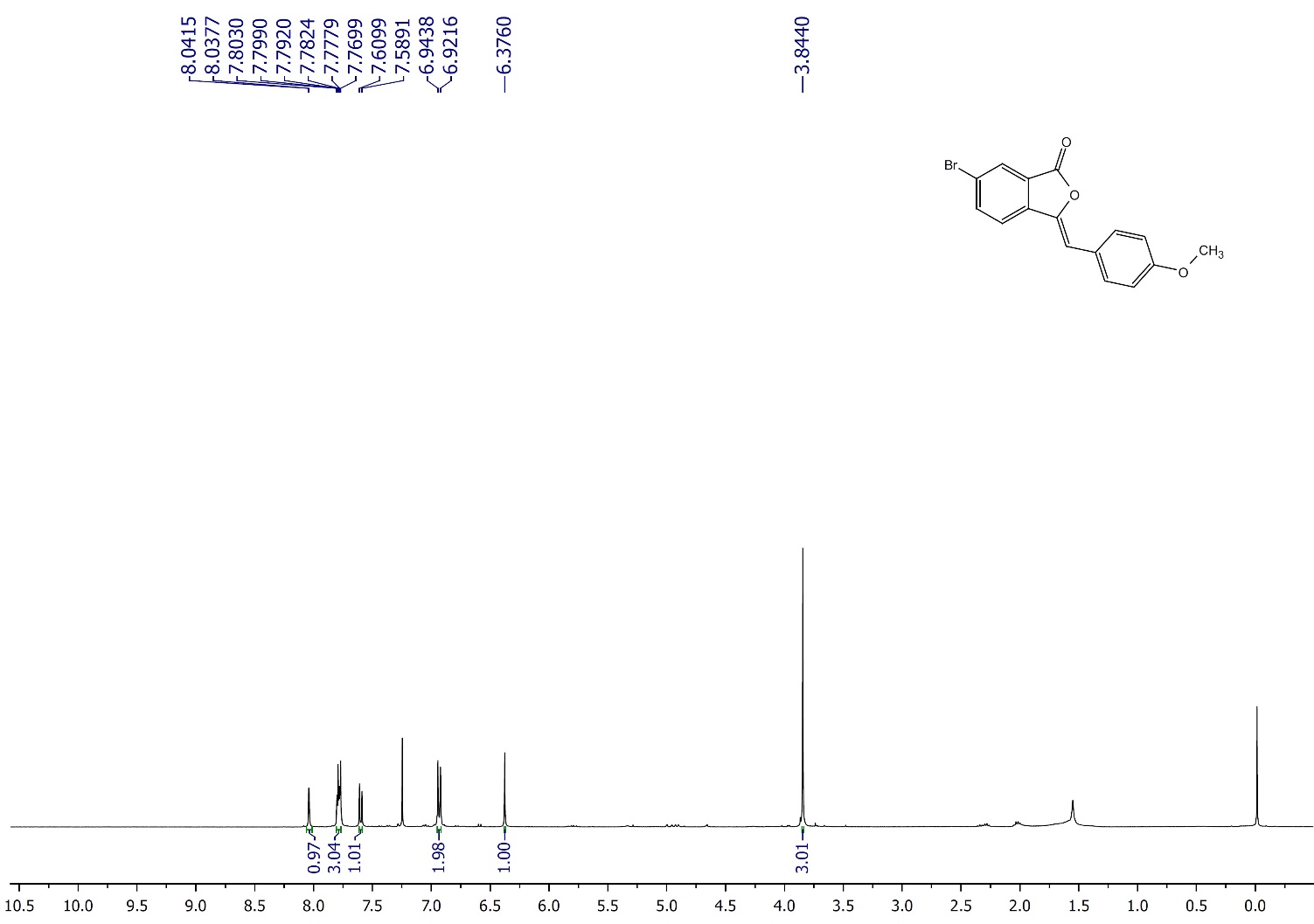


^1^H-NMR of **28k**.

**
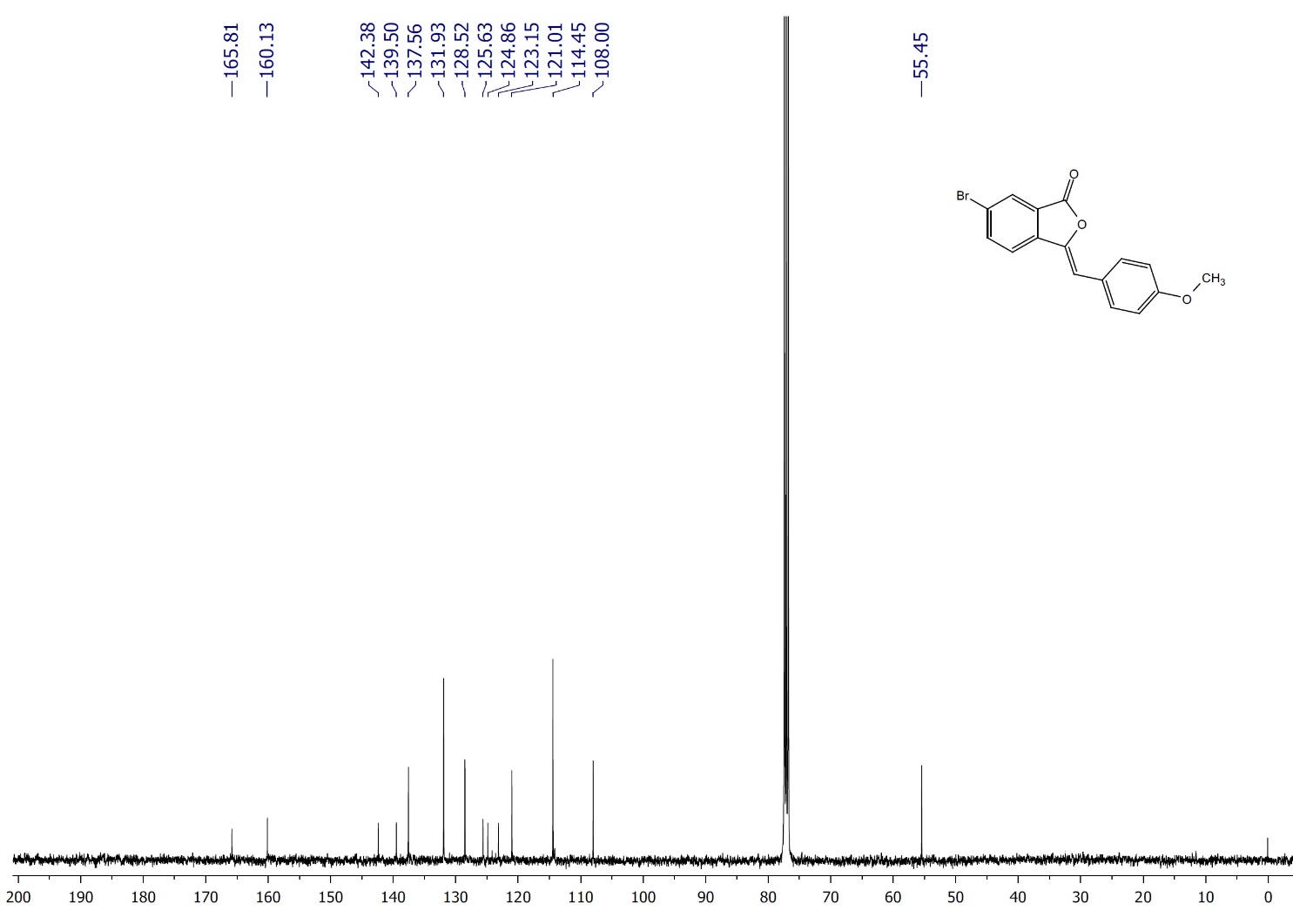
**

^13^C-NMR of **28k**.


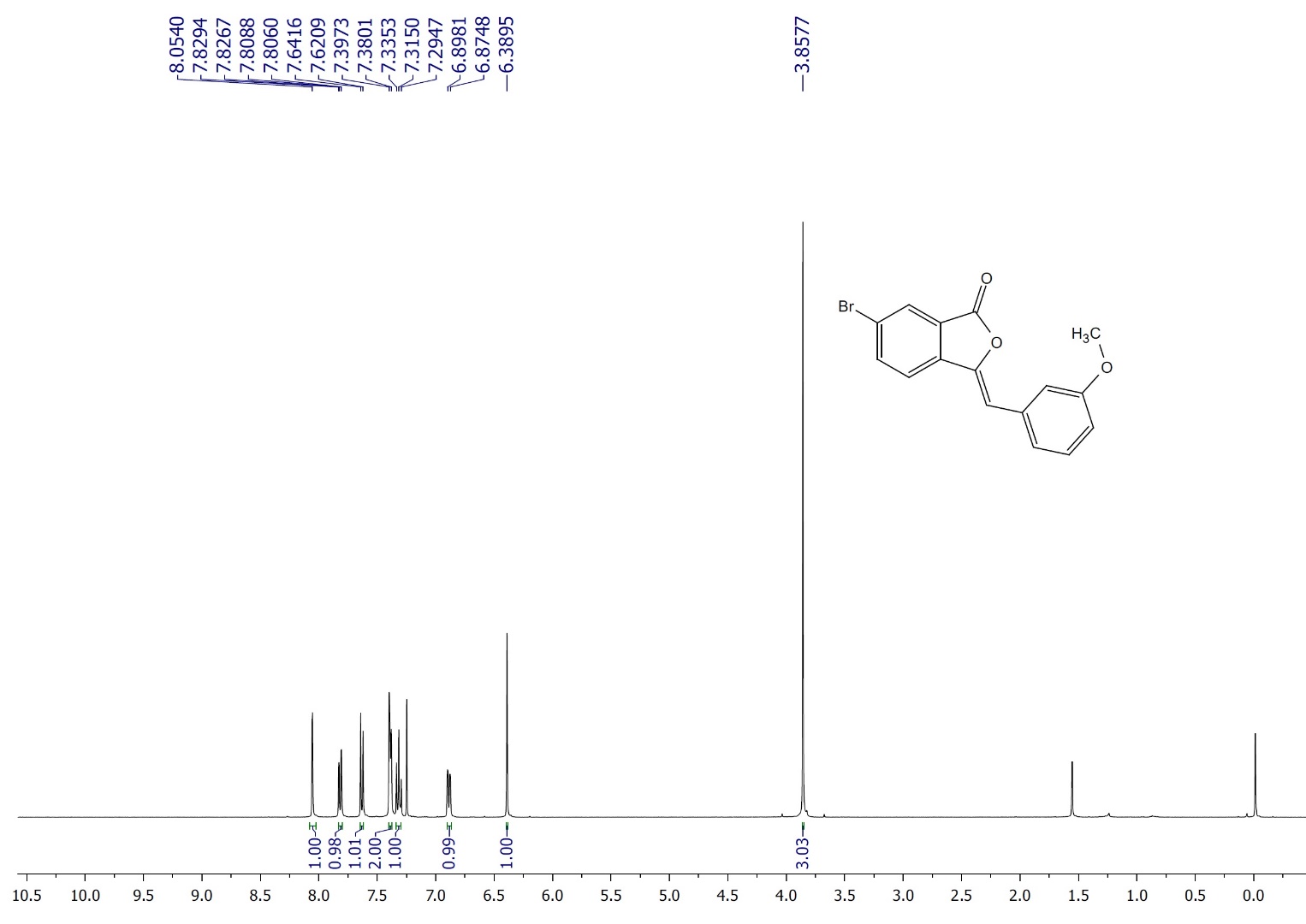


^1^H-NMR of **28l**.


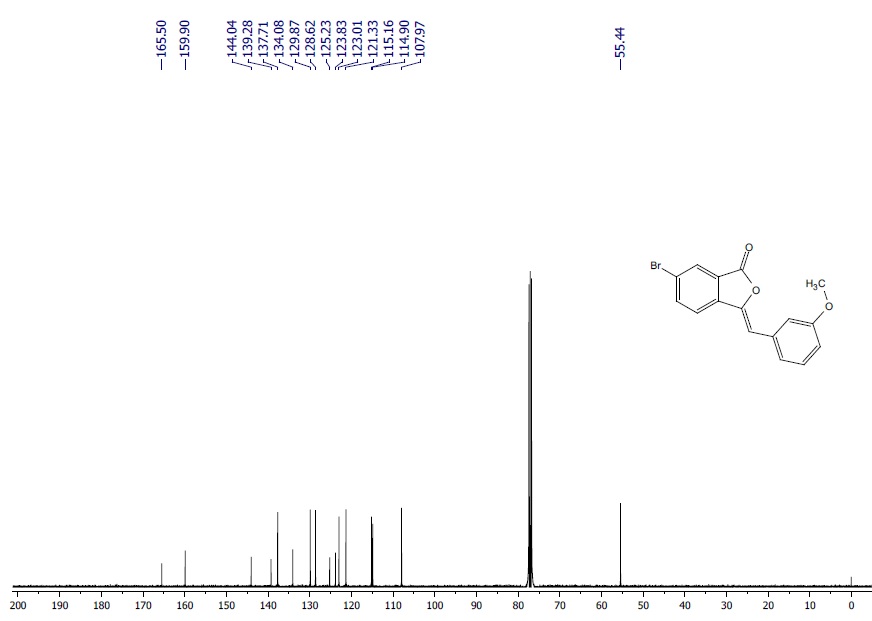


^13^C-NMR of **28l**.

**
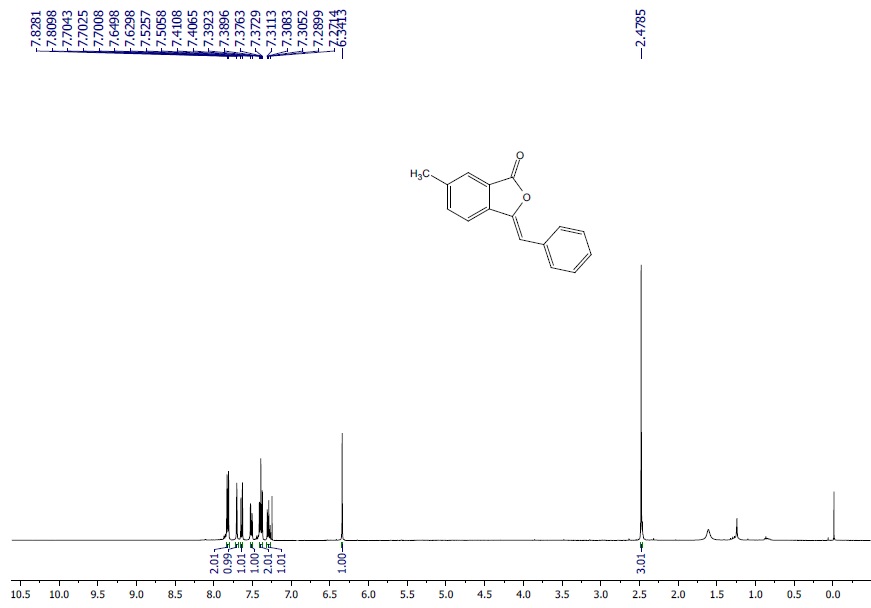
**

^1^H-NMR of **28m**.


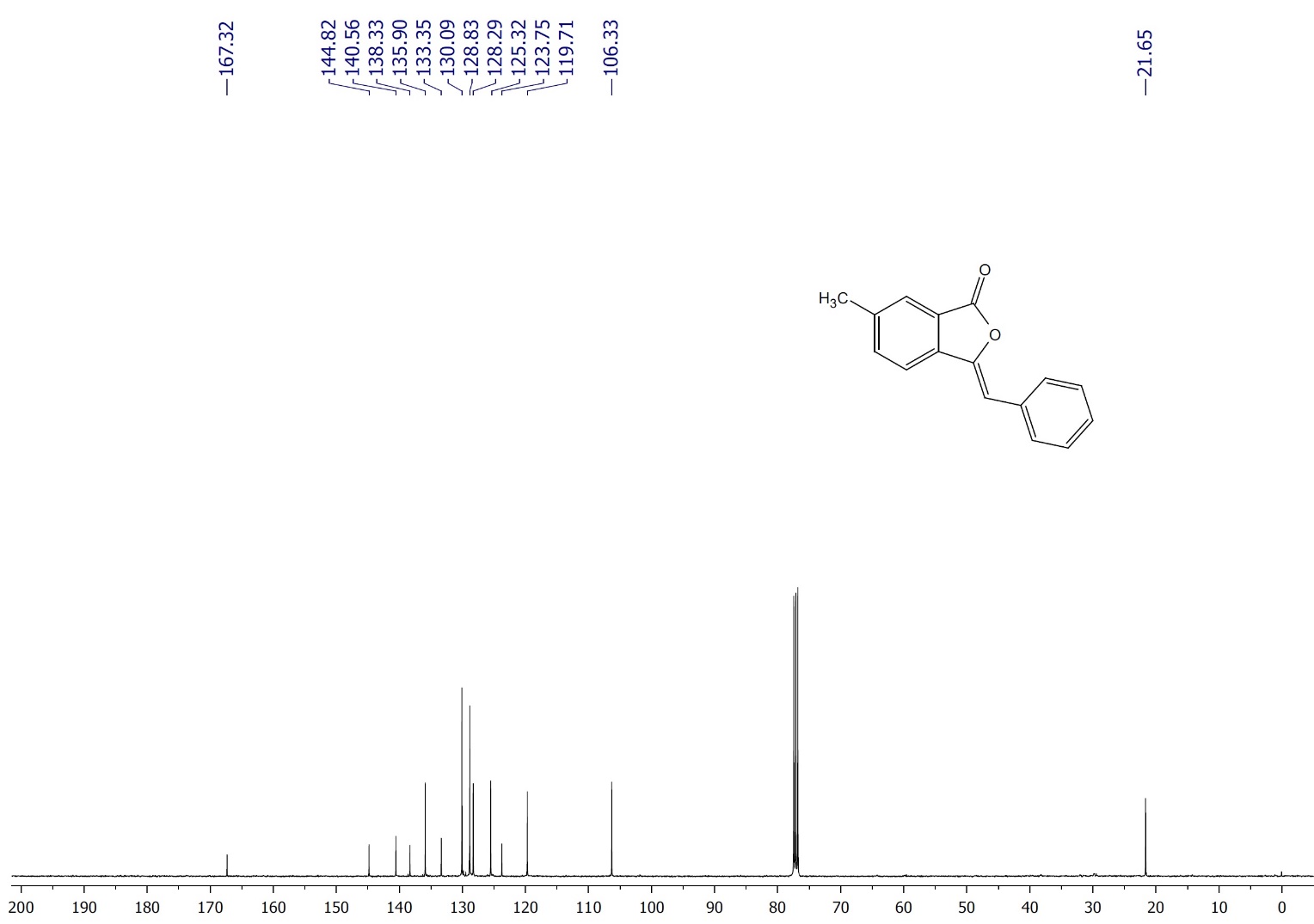


^13^C-NMR of **28m**.


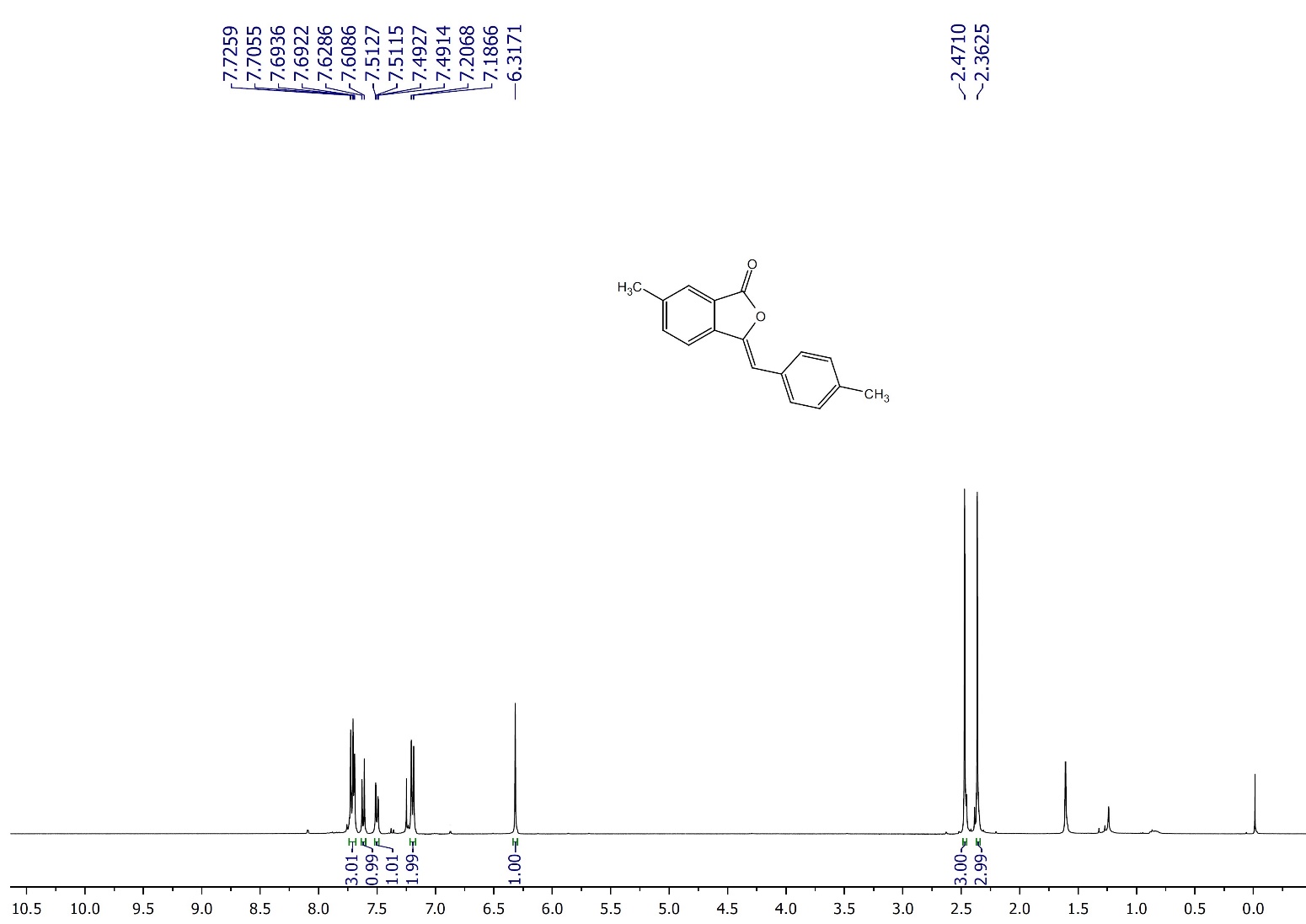


^1^H-NMR of **28n**.

**^
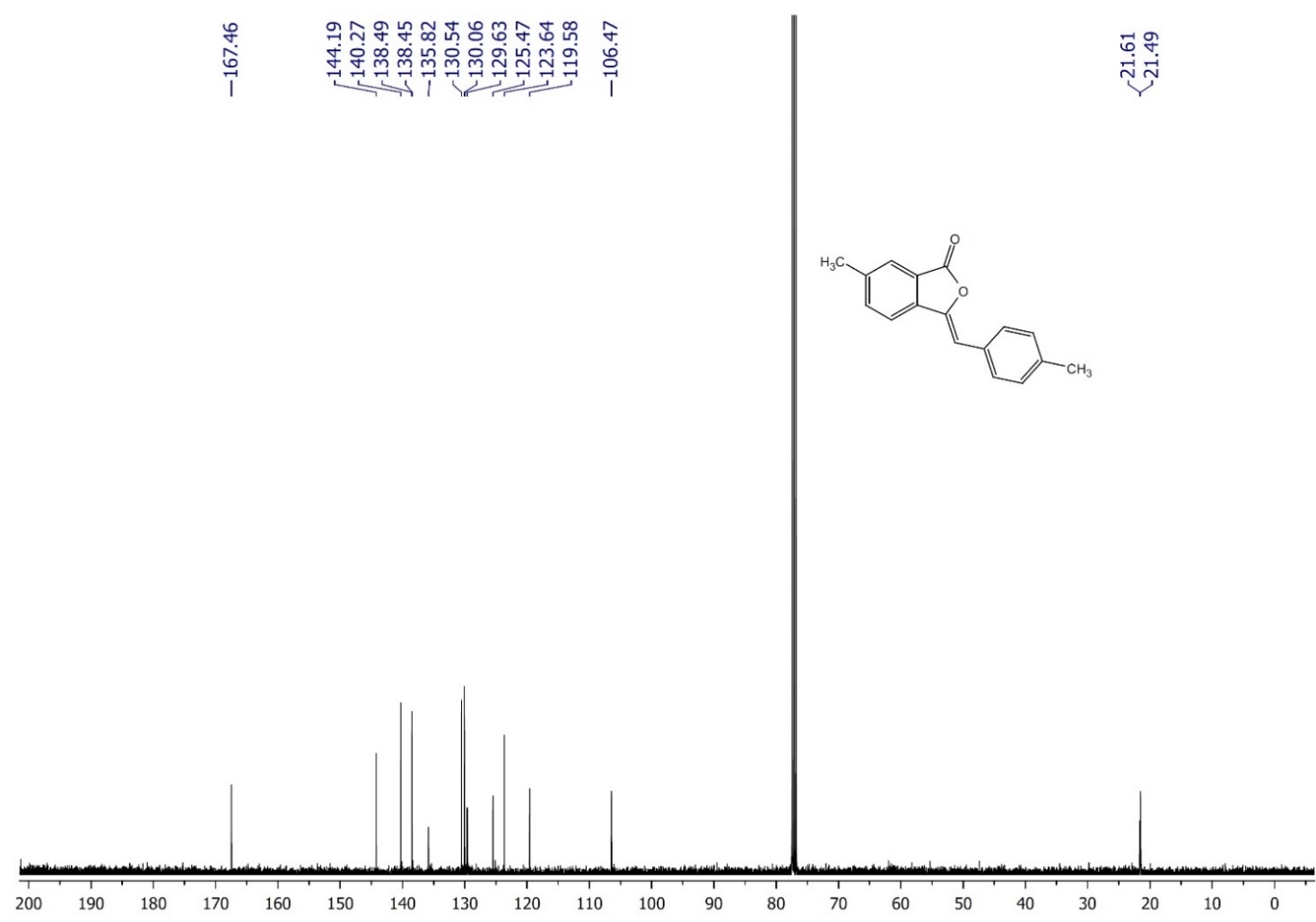
^**

^13^C-NMR of **28n**.


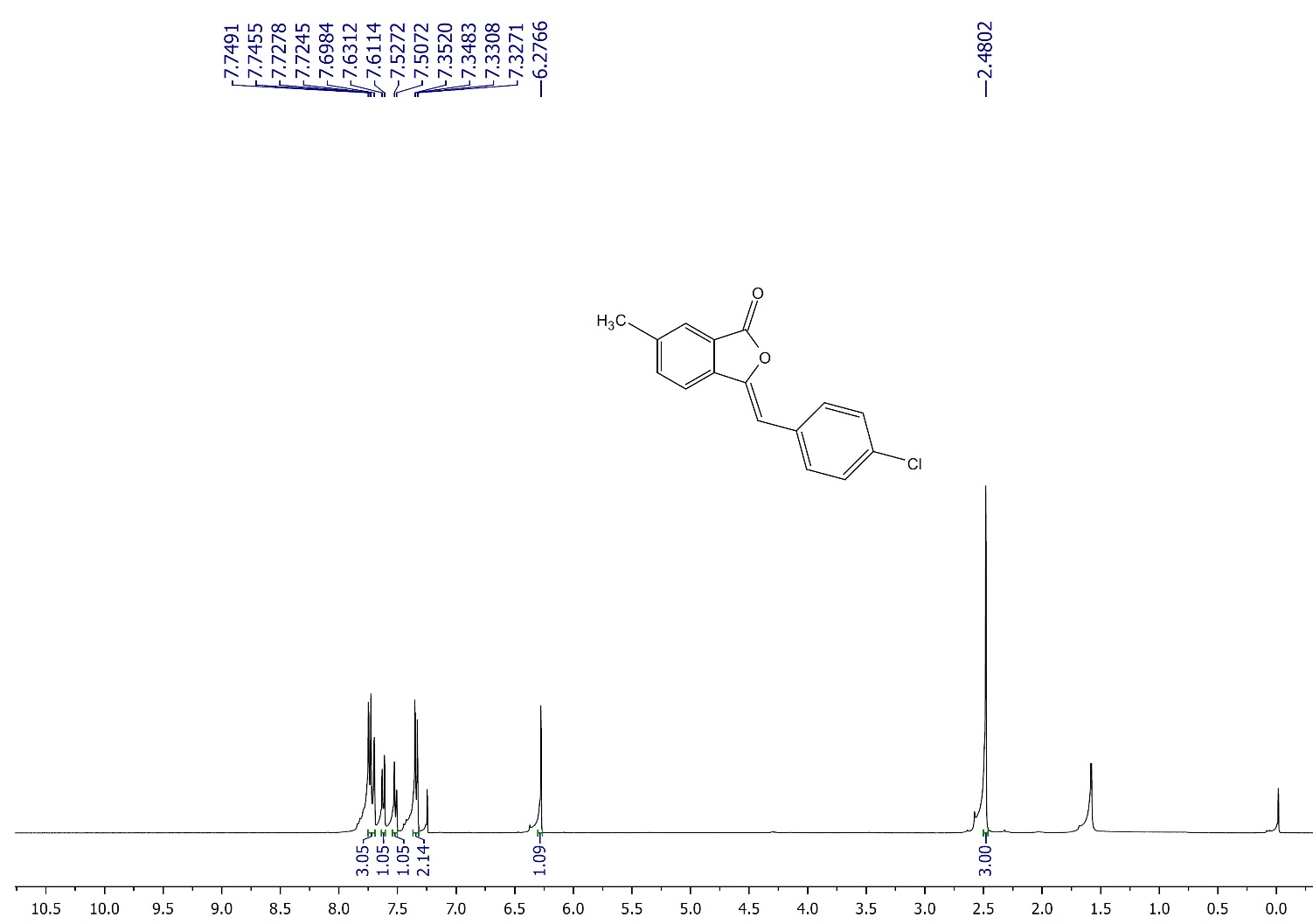


^1^H-NMR of **28o**.

**^
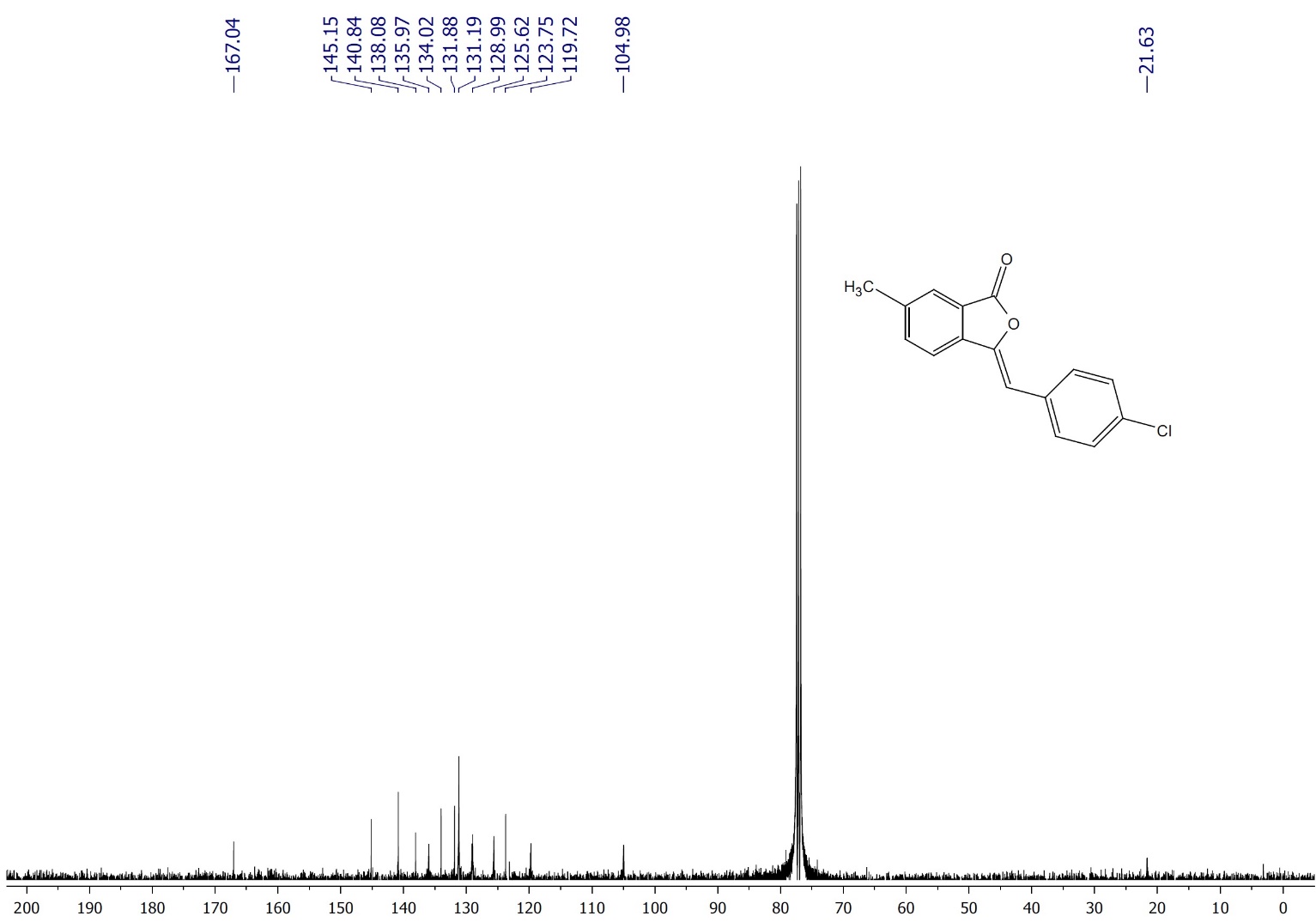
^**

^13^C-NMR of **28o**.


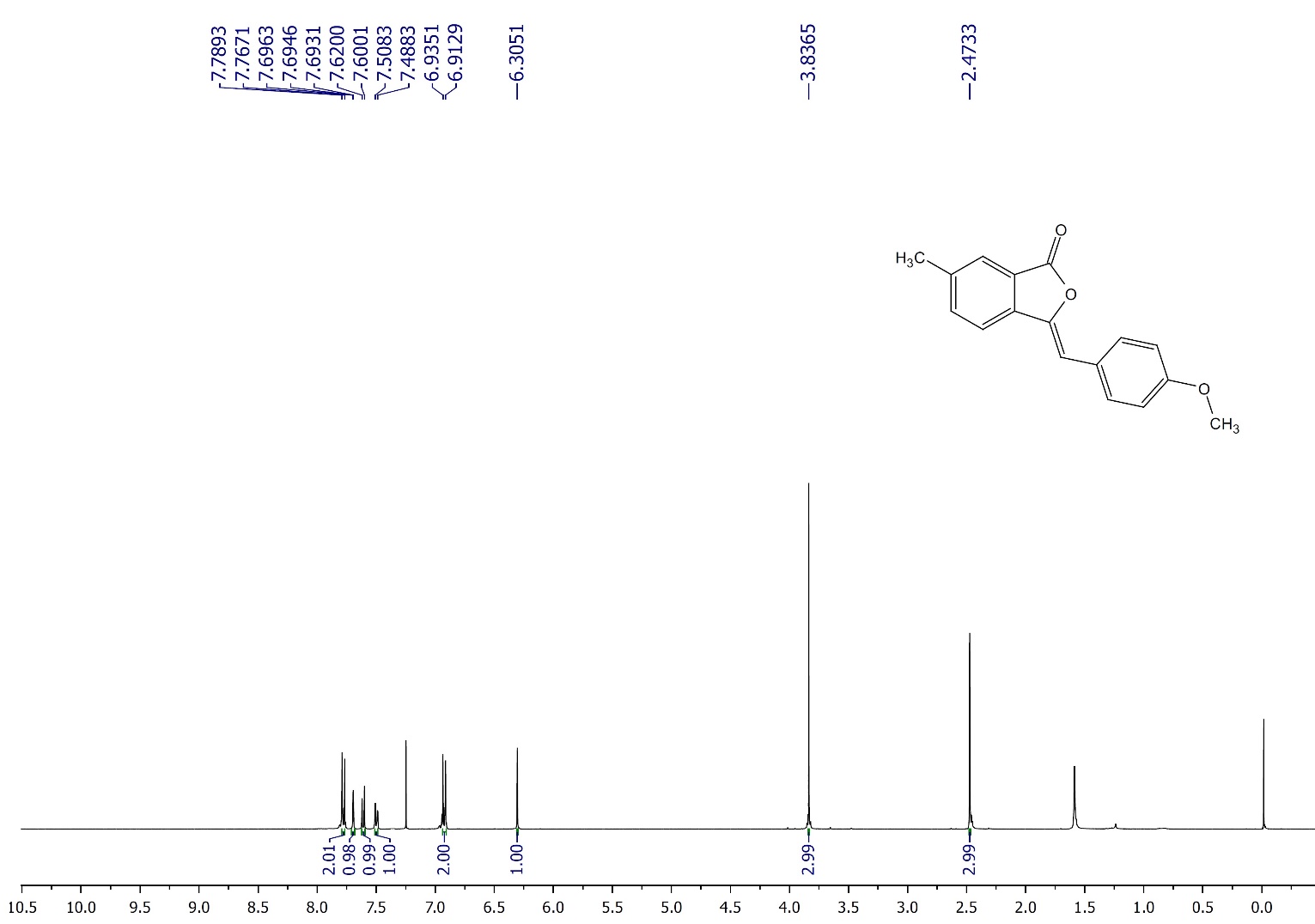


^1^H-NMR of **28p**.


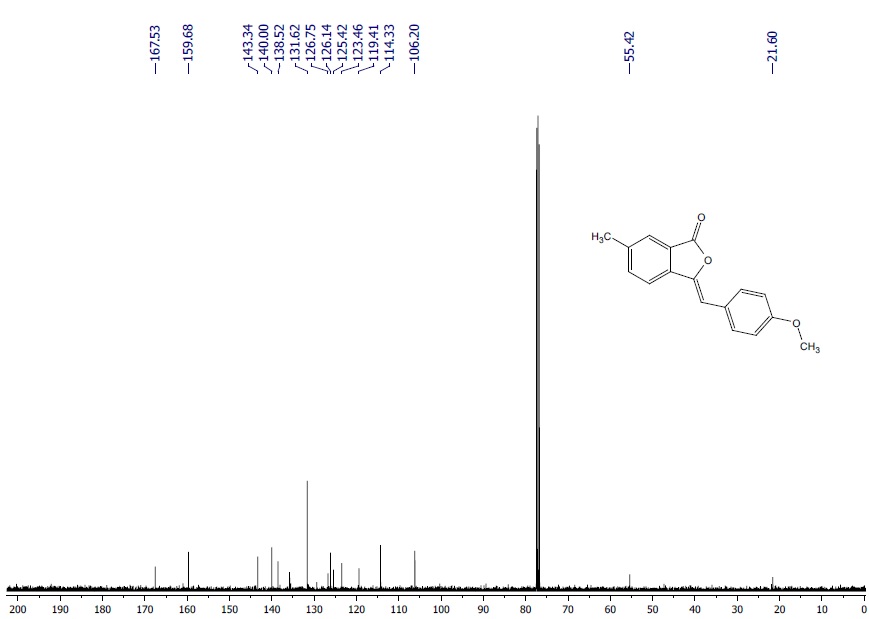


^13^C-NMR of **28p**.


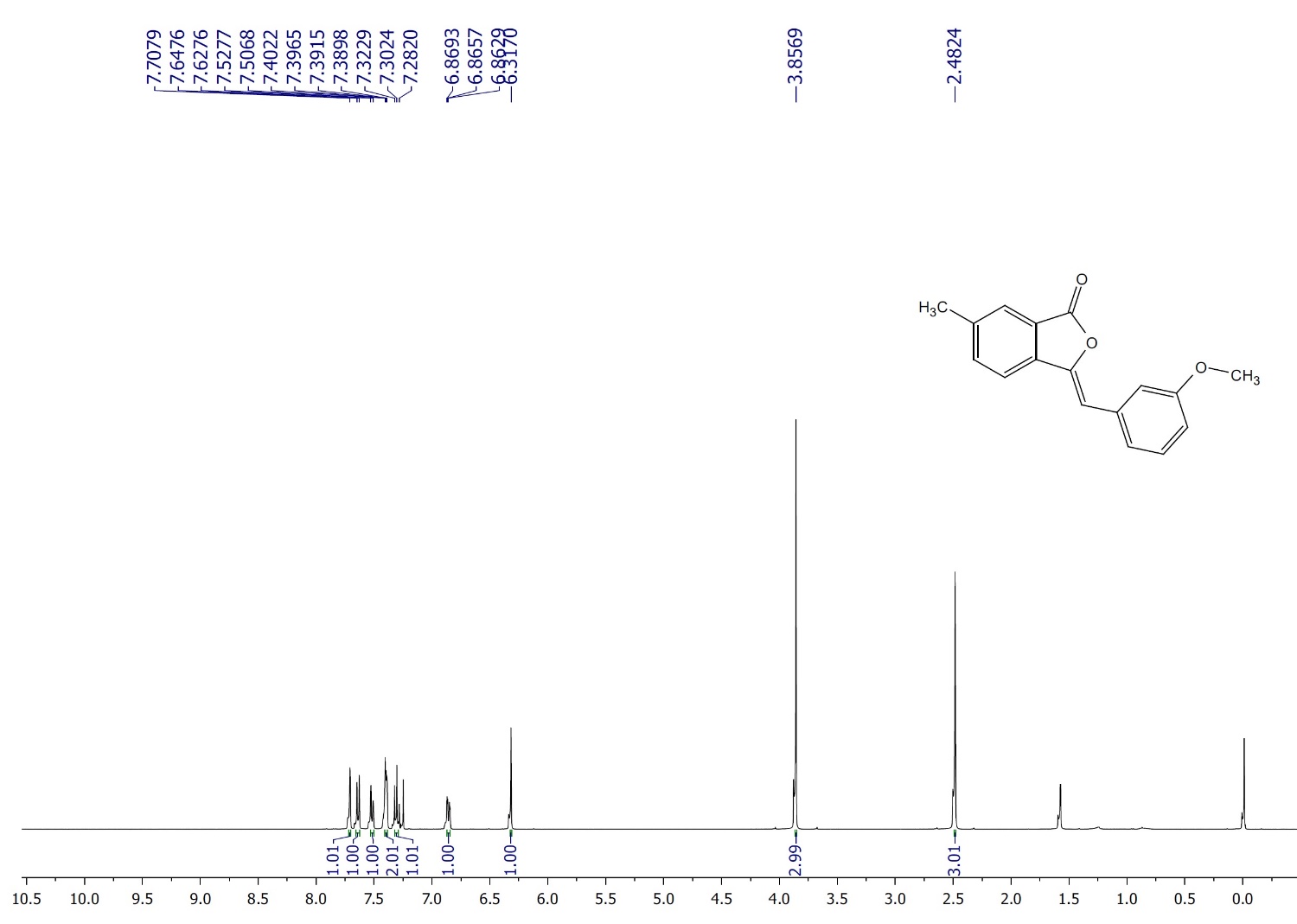


^1^H-NMR of **28q**.

**^
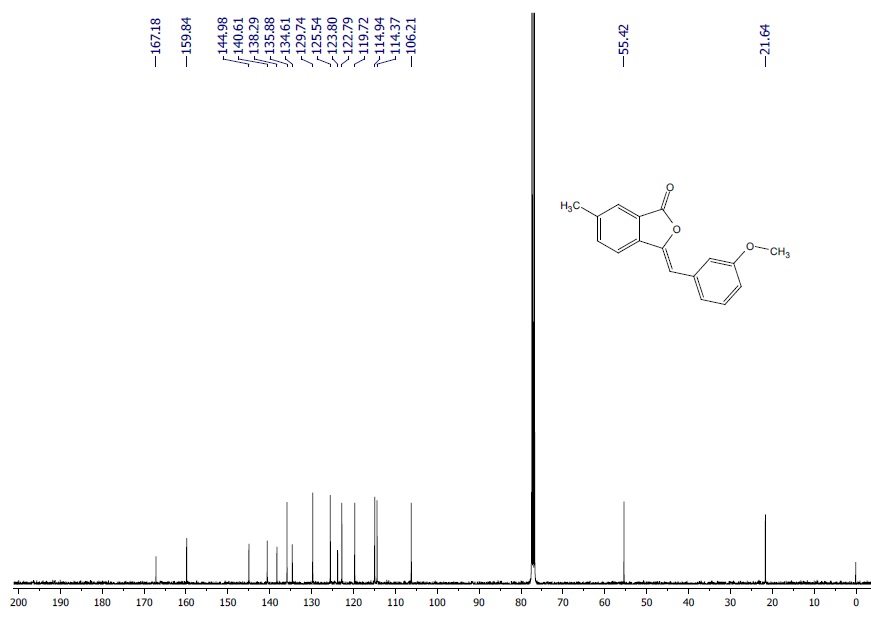
^**

^13^C-NMR of **28q**.


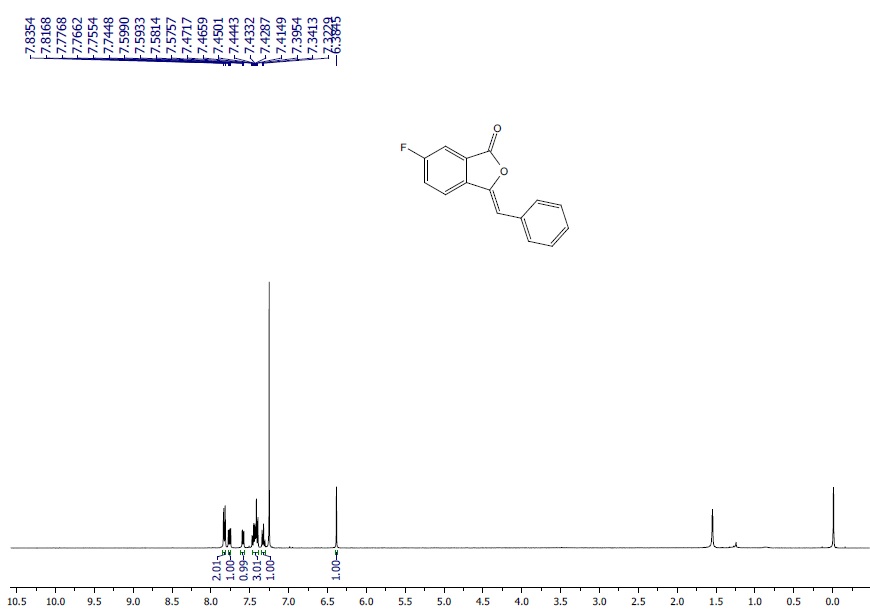


^1^H-NMR of **28r**.


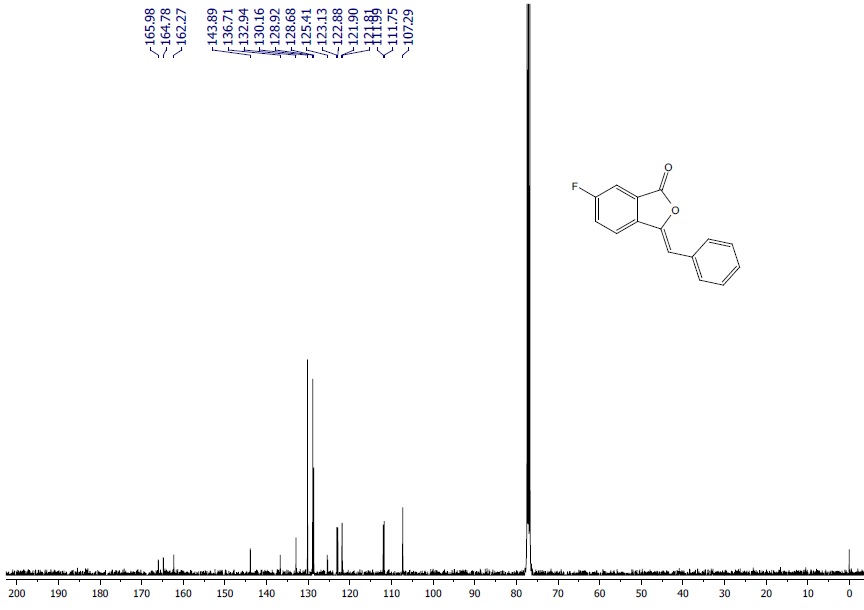


^13^C-NMR of **28r**.


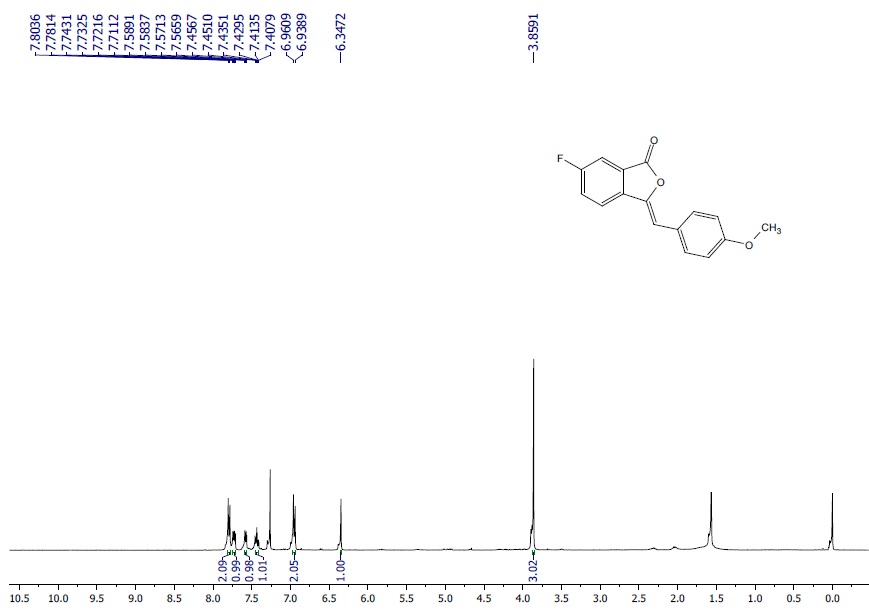


^1^H-NMR of **28s**.


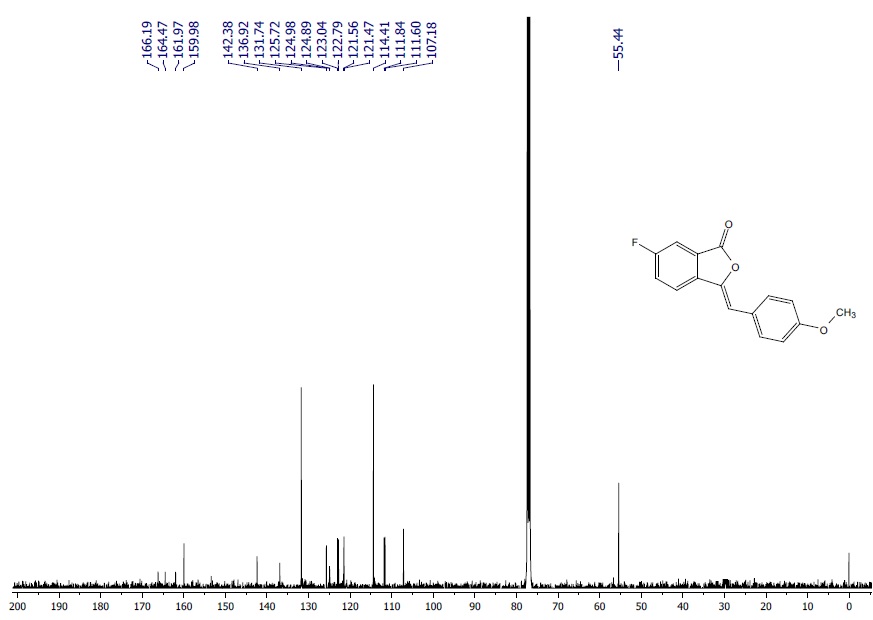


^13^C-NMR of **28s**.


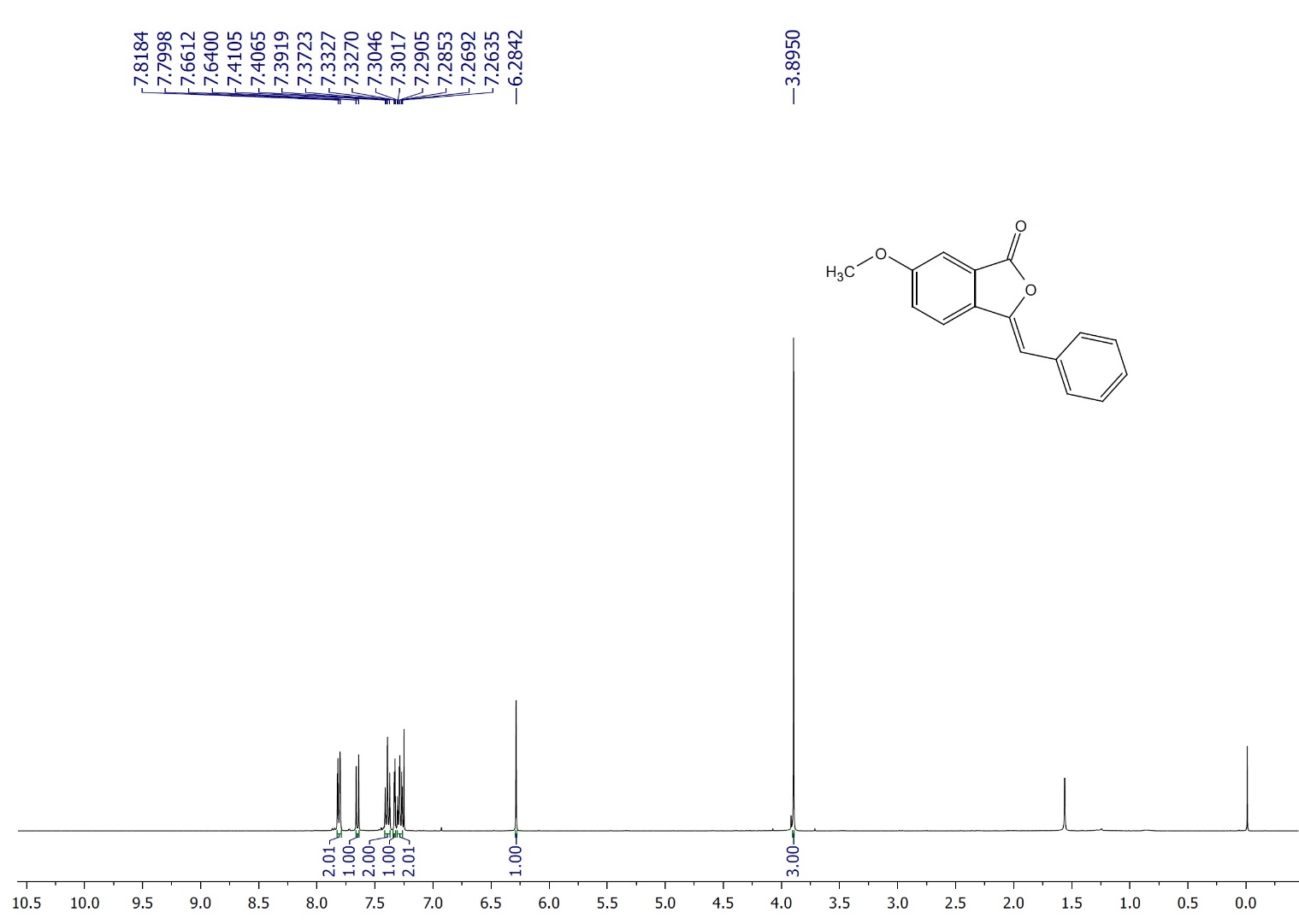


^1^H-NMR of **28t**.


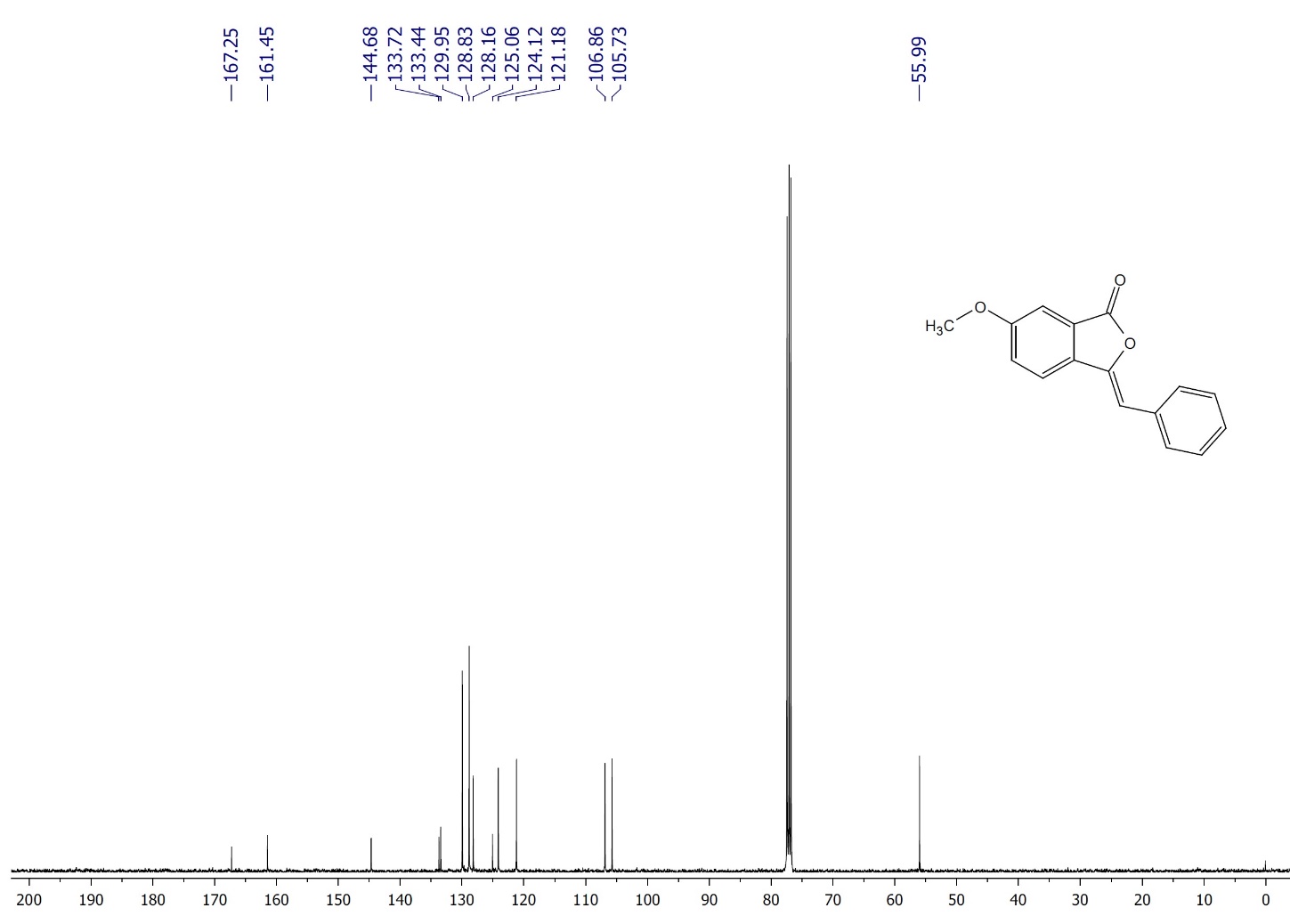


^13^C-NMR of **28t**.

3. Biological Methods

**3.1 *In vitro* antioxidant DPPH radical scavenging activity**^2^

In DPPH radical scavenging method the sample at different concentrations ranging from 10 to 100 μg mL^-1^ was mixed with 1.5 mL of a DPPH methanolic solution (20 mg L^-1^). Pure methanol was taken as control and ascorbic acid (vitamin C) was used as a reference compound. The percent of DPPH decolouration of the sample was calculated according to the formula.

The decolouration was plotted against the sample concentration and a logarithmic regression curve was established in order to calculate the IC_50_. The results are expressed as antiradical efficiency (AE), which is 1000-fold inverse of the IC_50_ value.

**3.2 Platelet aggregation inhibitory activity evaluation**^3,4^

All synthesized (*Z*)-3-benzylideneisobenzofuran-1(*3H*)-one analogues **(28a-t)** were dissolved in DMSO before testing. In order to eliminate the effects of the solvent on aggregation, the final concentration of DMSO was fixed at 0.5%. Arachidonic acid (AA), EDTA (disodium salt), bovine serum albumin and dimethyl sulfoxide (DMSO) were purchased from Sigma Chemical Co.

**Platelet Aggregation inhibitory bioassay**

Blood was collected from the rabbit marginal ear vein (several studies established that rabbit platelets are surrogate to human platelets both *in vitro* as well as *in vivo*)^5^ and was mixed with EDTA to a final concentration of 6 mM. It was centrifuged for 10 min at 90 g at room temperature, and the supernatant was obtained as platelet-rich plasma. The latter was further centrifuged at 500 g for 10 min. The platelet pellets were washed with Tyrode’s solution (Ca^+2^-free) containing 2mM EDTA, 0.1 mg/mL and 3.5 mg/mL bovine serum albumin, and centrifuged at 500 g for 10 min. Then, the pellets were washed with Tyrode’s solution without EDTA. After centrifugation under the same conditions, the platelet pellets were finally suspended in Tyrode’s solution of the following composition (mM): NaCl (136.8), KCl (2.8), NaHCO_3_ (11.9), MgCl_2_ (2.1), NaH_2_PO_4_ (0.33), CaCl_2_ (1.0), and glucose (11.2) containing bovine serum albumin (0.35%).

Aggregation was measured by a turbidimetric method using a Lumi-aggregometer (Chrono-Log Corp., Havertown, PA). All glassware was siliconized. Three minutes before the addition of the aggregation inducer, the platelet suspension was stirred at 1200 rpm. The percentage of aggregation was calculated as follows (abs. = absorbance):

Percent aggregation was expressed assuming the absorbance of platelet suspension as 0% aggregation and the absorbance of platelet-free Tyrode’s solution as 100% aggregation. For each compound IC_50_ values were calculated by SigmaPlot.

3.3 *Molecular docking studies:*

The molecular docking studies were performed using SYBYL-X 2.1.1 software (Tripos International)^6^. The crystal structure of antioxidant target (PDB ID: 3MNG)^7^ and antiplatelet target (PDB ID: 2OYE)^8^ co-crystallized with Dithiothreitol (Ligand ID: DID) and Indomethacin (Ligand ID: IM8), respectively, were used for molecular docking studies. The binding affinities were predicted by the Sybyl docking total score upon docking with the Surflex-Dock program (Sybyl X 2.0). Compounds were docked into the active site of the target peroxiredoxins 5 (Prxs) DTT complex (PDB ID: 3MNG) were taken from the Protein Data Bank (<http://www.rcsb.org/pdb>).^7,9^ Hydrogen bonds (H-bonds, with a donor-receptor distance of 3Å) between the ligand and amino acids in the binding site of the protein were used for the ranking of the compounds. The binding affinities were measured as 'total’ docking score. The mode of interaction of the co-crystallized ligand dithiothreitol (DTT) within the crystal structure of enzyme in complex was used as a reference binding model. The docking program parameter reliability was measured in terms of root-mean-square-deviation (RMSD) values in between the co-crystallized ligand and redocked poses. (see Table 1 in SI). Prior to molecular docking the protein structures were prepared using standard protocol of structure preparation tool of SYBYL-X suite that includes addition of hydrogen atoms, assignment of charges (Amber7FF99:Protein/Gasteiger-Marsili:Ligand),^10^ side chain optimization and minimization. The Surflex-Dock (SFCX) search algorithm[2](#_ENREF_2) was used to dock the co-crystallized ligand (ID: IM8, DID) and synthesized compounds. The ligands were prepared by ligand preparation module of SYBYL-X suite. During docking, protein was kept rigid while ligand was treated as fully flexible. A protomol-based method^10^ and empirically derived scoring function implemented in Surflex-Dock was used to calculate the interaction of the ligands and proteins. The scoring functions includes hydrophobic, polar, repulsive, entropic, solvation and crash terms. The Surflex-Dock scores are expressed in -Log10 (K d) units to represent binding affinities.

**4. Detailed Description for Antioxidant molecular docking for binding studies**

**Table 1.** Comparison of binding affinity of promising antioxidant active (*Z*)-3-benzylideneisobenzofuran-1(*3H*)-ones analogues, and standard drugs (ascorbic acid) against antioxidant target protein (PDB ID: 3MNG).

| Compound Name | Total Score | Amino acid involved in active pocket in 4 Ă | Involved group of Amino Acid | Length of H-bond Ă | No. of Hydrogen Bond |
| --- | --- | --- | --- | --- | --- |
| **28k-MNG** | 3.9321 | Lys--49, Pro-45, Thr-44, Cys-47, Arg-127, Gly-46, Pro-40, Thr-147, Leu-112, Ile-119, Leu-116, Phe-120 | Cys-47 (Backbone)  Arg-127 (Sidechain) | 1.9  2.3 | 2 |
| **28f-MNG** | 4.6899 | Lys-49, Gly46, Arg-127, Cys-47, Thr-44, Pro-40, Thr-147, Phe-120, Leu-116, Ile-119, Pro-45 | Gly-46 (Backbone)  Arg-127 (side chain)  Cys-47 (Backbone) | 2.1  2.3  1.8 | 3 |
| **28l-MNG** | 3.4080 | Ile-119, Ieu-116, Phe-120, Pro-140, Arg-127, Cys-47, Thr-44, Leu-149, Pro-45, Gly-46, Thr-147 | Thr-147 (sidechain)  Gly-46 (backbone) | 2.0  1.8 | 2 |
| **28d-MNG**  (inactive) | 2.9813 | Lys-49, Thr-50, Gly-46, Leu-149, Thr-147, Leu-116, Phe-12, Pro-40, Arg-127, Leu-112, Cys-47, Thr-44, Pro-45 | ---- | ---- | ---- |
| **Ascorbic acid-MNG** | 3.1764 | Gly-46, Cys-47, Leu-149, Arg-127, Thr-44, Pro-40, Phe-120, Leu-112, Leu-116, Thr-147, Pro-45 | Gly-46 (Backbone)  Arg-127 (Backbone)  Thr-44 (sidechain) | 1.8  1.8  2.0 | 3 |

The docking results for the standard reference **ascorbic acid** against antioxidant target showed a high binding affinity docking score (total score = **3.1764**) and forms three H-bond of length 2.0 and 1.8Å to the side chain and backbone of hydrophobic residue i.e., Threonine-147, Glycine-46 and Arginine-127. In docking pose, the chemical nature of binding site residues within a radius of 3Å from bound compound were basic (polar, hydrophobic and positive charged), that is, Leu-116, Leu-112, Leu-149 (leucine), Gly-46 (glycine), Arg-127 (arginine) and Pro-40, Pro-45 (proline); nucleophilic (polar, hydrophobic), that is, Thr-44, Thr-147 (threonine), Phe-120 (phenylalanine), Cys-47 (cysteine), thus, bound compound showed high binding affinity and hydrophobic interaction which may lead to more stability and activity (Fig. 4).

The docking results for compound **28k** against antioxidant target (PDB ID: 3MNG) showed a high binding affinity docking score indicated by a total score of **3.9321** and forms two H-bond of length 2.3 and 1.9 Å to the side chain and backbone hydrophobic residue that is, Arginine-127 and Cystein-47. In docking pose, the chemical nature of binding site residues within a radius of 3Å from bound compound were basic (polar, hydrophobic and positive charged), that is, Gly-46 (glycine), Lys-49 (lysine); Leu-112, Leu-116 (leucine), Ile-119 (isoleucine), Pro-40, Pro-45 (proline), Arg-127 (arginine); nucleophilic (polar, hydrophobic), that is, Cys-47 (cysteine), Phe-120 (phenylalanine) and Thr-44, Thr-147 (threonine), thus, high binding affinity and strong hydrophobic interaction has been displayed by **28k** which may lead to more stability and activity (Fig. 5a).

Likewise, for compound **28f** against anti-oxidant target, the docking results showed a high binding affinity docking score (total score - **4.6899)** and forms three H-bond of length 2.3, 2.1 and 1.8Å to the side chain and backbone of hydrophobic residue i. e, Arginine-127, Glycine-46 and Cystein-47. In docking pose, the chemical nature of binding site residues within a radius of 3Å from bound compound were basic (polar, hydrophobic and positive charged), that is, Lys-49 (lysine), Gly-46 (glycine), Arg-127(arginine), Pro-40, Pro-45 (proline) and Leu-116 (leucine), Ile-119 (Isoleucine); nucleophilic (polar, hydrophobic), that is, Cys-47 (cysteine), Thr-147, Thr-44 (threonine), Phe-120 (phenylalanine); thus, bound compound (**28f**) showed high binding affinity and hydrophobic interaction which may lead to more stability and activity (Fig. 5b).

Likewise, the docking results for compound **28l** against anti-oxidant target (PDB ID: 3MNG) showed a high binding affinity docking score indicated by a total score of **3.4080** and forms two H-bond of length 2.0 and 1.8Å to the side chain and backbone of hydrophobic residue i.e., Threonine-147 and Glycine-46. In docking pose, the chemical nature of binding site residues within a radius of 3Å from bound compound were nucleophilic (polar, hydrophobic), that is, Thr-147, Thr-44 (threonine), Phe-120 (phenylalanine), Cys-47 (cysteine); basic (polar, hydrophobic and positive charged), that is, Leu-116, Leu-149 (leucine), Ile-119 (Isoleucine), Gly-46 (glycine), Arg-127 (arginine) and Pro-40, Pro-45 (proline), thus, bound compound showed high binding affinity and strong hydrophobic interaction which may lead to more stability and activity (Fig. 5c).

In order to further analyze our *in vitro* and docking results; the inactive compound **28d** was subjected to docking against antioxidant target. However, it showed a total score of **2.9813** indicating low binding affinity. In docking pose, the chemical environment of binding site residues within a radius of 3Å from bound compound were nucleophilic (polar, hydrophobic), i.e., Cys-47 (cysteine), Thr-50, Thr-147, Thr-44 (threonine), Phe-12 (phenylalanine); basic (polar, hydrophobic and positive charged), that is, Lys-49 (lysine), Gly-46 (glycine), Arg-127(arginine), Pro-40, Pro-45 (proline) and Leu-112, Leu-149, Leu-116 (leucine), thus, bound compound showed low binding affinity and weak hydrophobic interaction which may be responsible for less stability and activity (Fig. 5d).

**5. Detailed Description for Antiplatelet molecular docking for binding studies**

**Table 2.** Comparison of binding affinity of active functionalized (*Z*)-3-benzylideneisobenzofuran-1(*3H*)-ones analogues **28k**, **28s**, **28f**, **28l**, and **28j**, and standard drugs reference (acetyl salicylic acid) against antiplatelet target protein (PDB ID: 2OYE).

| Compound Name | Total Score | Amino acid involved in active pocket in 4 Ă | Involved group of Amino Acid | Length of H-bond Ă | No. of Hydrogen Bond |
| --- | --- | --- | --- | --- | --- |
| **28k-2OYE** | 5.1953 | Leu-93, Ile-523, Pro-528, Arg-12, Glu-524, Ala-527, Phe-518, Trp-387, , Leu352, Tyr-348, Ser-530, Val-349, Leu-531, Leu-359, Tyr355, Val116, Ile-86 | Ser-530 (side chain) | 2.0 | 1 |
| **28s-2OYE** | 5.7131 | Gln-192, His-90, Ser-353, Ile--523, Tyr--355, Leu-359, Val-349, Leu-531, Ala-527, Ser-530, Gly-526, Phe-518, Leu-352, Ile-512, Ser-516, Asn-515, Thr-94, Gly-354 | Ser-516 (Sidechain) |  |  |
| **28f-2OYE** | 5.1010 | Glu-524, Pro-86, Ser-353, Ile-523, Phe-518, Leu-352, Val-349, Leu-531, Ser-530, Ala-527, Leu-359, Val-aa6, Tyr-355, Leu-93, Ile-89, Arg-120 | - | - | - |
| **28l-2OYE** | 5.1184 | Ile-89, Val-116, Leu-93, Tyr-355, Ile-523, Ser-353, Phe-518, Leu-352, Val-349, Trp-387, Tyr-348, Tyr-385, Ser-530, Ala-527, Leu-531, Leu-359, Pro-86, Glu-524, Arg-120 | - | - | - |
| **28j-2OYE**  (inactive) | 4.1714 | Pro-84, Pro86, Ser-85, Ile-89, Glu-524, Ala-527, Ile 523, Val-349, Leu-531, Tyr-355, Val-116, Leu-93, Val-119, Arg-120 | - | - | - |
| **Acetyl salicylic acid** | 4.4803 | Leu-352, Ile-523, Phe-518, Ala-527, Leu-531, Tyr-348, Val-349, Trp-387, Tyr-385, Ser-530, Ser-353 | Ser-530 (Sidechain) | 2.0 | 1 |

The docking results for acetyl salicylic acid against antiplatelet target showed low binding affinity docking score indicated by a total score of **4.4803** and forms a H-bond of length 2.0 Å to the side chain hydrophobic aliphatic residue that is, Serine-530. In docking pose, the chemical nature of binding site residues within a radius of 3Å from bound compound were hydrophobic, that is, Leu-531, Leu-352 (leucine), Ile-523 (isoleucine), Val-349 (valine) Ala-527 (alanine); aromatic (hydrophobic), that is, Phe-518 (phenylalanine), Tyr-348, Tyr-385 (tyrosine), Trp-387 (tryptophan) and nucleophilic (polar, hydrophobic), that is, Ser-353, Ser-530 (serine), thus, bound compound showed high binding afﬁnity and strong hydrophobic interaction which may lead to more stability and activity (Fig. 6f). Moreover, after analysing the chemical nature of binding site amino acid residues, it was revealed that, these compounds have propensity to makes hydrophobic interactions and multiple H-bonds with hydrophilic residues of target protein. Thus comparing the binding affinities of C-3 tethered isobenzofuran-1(*3H*)-one analogues with ascorbic acid showed these compounds have better antioxidant activity (Fig. 6a).

The docking results for **28k** against antiplatelet target (PDB ID: 2OYE) showed a high binding affinity docking score indicated by a total score of **5.1953** and forms a H-bond of length 2.1 Å to the side chain hydrophobic aliphatic residue i.e., Serine-530. In docking pose, the chemical nature of binding site residues within a radius of 4Å from bound compound were hydrophobic, that is, Ala-527 (alanine), Ile-86, Ile-523 (isoleucine), Leu-93, Leu-352, Leu-359, Leu-531 (leucine), Val-116, Val-349 (valine), Pro-528 (proline); basic (polar, hydrophobic and positive charged), that is, Arg-12 (arginine); nucleophilic (polar, hydrophobic), that is, Ser-530 (serine); aromatic (hydrophobic), that is, Phe-518 (phenylalanine), Tyr-355, Tyr-348 (tyrosine), Trp-387 (Tryptophan); and acidic (polar, negative charged), that is, Glu-524 (Glutamic acid); thus, bound compound showed high binding afﬁnity and strong hydrophobic interaction which may, consequently, lead to more stability and activity (Fig. 6b).

Likewise, the docking results for **28s** against antiplatelet target showed a high binding affinity docking score indicated by a total score of **5.7131** and forms a H-bond of length 2.0 Å to the side chain hydrophobic aliphatic residue that is, Serine-516. In docking pose, the chemical nature of binding site residues within a radius of 3Å from bound compound were aromatic (hydrophobic), that is, Phe-518 (phenylalanine), Tyr-355 (tyrosine); nucleophilic (polar, hydrophobic), that is, Ser-353, Ser-530, Ser-516 (serine); hydrophobic, that is, Leu-359, Leu-531, Leu-352 (leucine), Ile-512, Ile-523 (isoleucine), Val-349 (valine), Ala-527 (alanine); Gly-354, Gly-526 (glycine); polar amide, that is, Gln-192 (glutamine), Asn-515 (asparagine); nucleophilic (polar, hydrophobic), that is, Thr-94 (threonine) and basic (polar, hydrophobic and positive charged), that is, His-90 (histidine); thus, bound compound showed high binding affinity and strong hydrophobic interaction which may lead to more stability and activity (Fig. 6c).

Similarly, the docking results for **28f** against antiplatelet target (PDB ID: 2OYE) showed a high binding affinity docking score (total score = **5.1010**). In docking pose, the chemical nature of binding site residues within a radius of 3Å from bound compound were hydrophobic residue, that is, Ile-89, Ile-523 (isoleucine), Ala-527 (alanine), Leu-93, Leu-531, Leu-359 (leucine), Val-116, Val-349, (valine), Pro-86 (proline); aromatic (hydrophobic), that is, Phe-518 (phenylalanine), Tyr-355 (tyrosine); nucleophilic (polar, hydrophobic), that is, Ser-353, Ser-530 (serine); acidic (polar, negative charged), that is, Glu-524 (Glutamic acid) and basic (polar, hydrophobic and positive charged), that is, Arg-120 (arginine); thus, bound compound showed high binding affinity and strong hydrophobic interaction which may lead to more stability and activity (Fig. 6d).

Similarly, the docking results for **28l** against antiplatelet target (PDB ID: 2OYE) showed a high binding affinity docking score indicated by a total score of **5.1184**. In docking pose, the chemical nature of binding site residues within a radius of 3Å from bound compound were acidic (polar, negative charged), that is, Glu-524 (Glutamic acid); basic (polar, hydrophobic and positive charged), that is, Arg-120 (arginine); Ile-89, Ile-523 (isoleucine), Leu-93, Leu-352, Leu-531, Leu-359 (leucine), Val-116, Val-349 (valine), Ala-527 (alanine); nucleophilic (polar, hydrophobic), that is, Ser-353, Ser-530 (serine); aromatic (hydrophobic), that is, Phe-518 (phenylalanine), Tyr-355, Tyr-348, Tyr-385 (tyrosine), Trp-387 (tryptophan); thus, bound compound showed high binding affinity and strong hydrophobic interaction which may lead to more stability and activity (Fig. 6e).

As observed in the case of antioxidant compound **28d**; the docking results for inactive compound **28j** against antiplatelet target (PDB ID: 2OYE) showed a low binding affinity docking score indicated by a total score of **4.1714**. In docking pose, the chemical nature of binding site residues within a radius of 3Å from bound compound were nucleophilic (polar, hydrophobic), that is, Ser-85 (serine); basic (polar, hydrophobic and positive charged), that is, Arg-120 (arginine); and acidic (polar, negative charged), that is, Glu-524 (Glutamic acid); hydrophobic, that is, Ala-527 (alanine), Ile-89, Ile-523 (isoleucine), Leu-93, Leu-531 (leucine), Val-116, Val-119, Val-349, (valine), Pro-84, Pro-86 (proline); aromatic (hydrophobic), that is, Tyr-355 (tyrosine). Thus, the bound compound **28j** showed low binding afﬁnity and weak hydrophobic interaction which may lead to less stability and activity (Fig. 6f).

**6. References**:

1. Chaudhary, S., Shyamlal, B. R. K., Yadav, L., Tiwari, M. K., & Kumar, K. Ag_2_O nanoparticle-catalyzed substrate-controlled regioselectivities: Direct access to 3-ylidenephthalides and isocoumarins. *RSC Advances*, **8**, 23152–23162, doi: 10.1039/c8ra03926g (2018).

2. Hernández-Vázquez, E., Castañeda-Arriaga, R., Ramírez-Espinosa, J. J., Medina-Campos, O. N., Hernández-Luis, F., Chaverri, J. P., & Estrada-Soto, S. 1,5-Diarylpyrazole and vanillin hybrids: Synthesis, biological activity and DFT studies. *European Journal of Medicinal Chemistry*, **100**, 106–118, doi:10.1016/j.ejmech.2015.06.010 (2015).

3. Che-Ming Teng, Wen-Ying Chen, Wun-Chang Ko, & Ouyang, C. Antiplatelet effect of butylidenephthalide. *Biochimica et Biophysica Acta (BBA) - General Subjects*, **924**, 375–382. doi: 10.1016/0304-4165(87)90151-6 (1987).

4. Chen, K.-S., Ko, F.-N., Teng, C.-M., & Wu, Y.-C. Antiplatelet and vasorelaxing actions of some benzylisoquinoline and phenanthrene alkaloids. *Journal of Natural Products*, **59**, 531–534, doi: 10.1021/np960354x (1996).

5. Packham, M., Rand, M., & Kinlough-Rathbone, R. Similarities and differences between rabbit and human platelet characteristics and functions. *Comparative Biochemistry and Physiology Part A: Physiology*, **103**, 35–54, doi: 10.1016/0300-9629(92)90239-m (1992).

6. Kumar, S., Yadav, D. K., Choi, E.-H., & Kim, M.-H. Insight from molecular dynamic simulation of reactive oxygen species in oxidized skin membrane. *Scientific Reports*, **8**, doi: 10.1038/s41598-018-31609-w (2018).

7. Perkins, A., Gretes, M. C., Nelson, K. J., Poole, L. B., & Karplus, P. A. Mapping the active site helix-to-strand conversion of CxxxxC Peroxiredoxin Q enzymes. *Biochemistry*, **51**, 7638–7650, doi: 10.1021/bi301017s (2012).

8. Harman, C. A., Turman, M. V., Kozak, K. R., Marnett, L. J., Smith, W. L., & Garavito, R. M. Structural basis of enantioselective inhibition of Cyclooxygenase-1 by S-α-substituted Indomethacin Ethanolamides. *Journal of Biological Chemistry*, **282**, 28096–28105, doi:10.1074/jbc.m701335200 (2007).

9. Portillo-Ledesma, S., Sardi, F., Manta, B., Tourn, M. V., Clippe, A., Knoops, B., Ferrer-Sueta, G. Deconstructing the catalytic efficiency of Peroxiredoxin-5 Peroxidatic Cysteine. *Biochemistry*, **53**, 6113–6125, doi: 10.1021/bi500389m (2014).

10. Yadav, D. K., Kumar, S., Choi, E.-H., Sharma, P., Misra, S., & Kim, M.-H. Insight into the molecular dynamic simulation studies of reactive oxygen species in native skin membrane. *Frontiers in Pharmacology*, **9**, doi:10.3389/fphar.2018.00644 (2018).
